# Unique Initiation and Termination Mechanisms Involved in the Biosynthesis of a Hybrid Polyketide-Nonribosomal Peptide Lyngbyapeptin B Produced by the Marine Cyanobacterium *Moorena bouillonii*

**DOI:** 10.1021/acschembio.3c00011

**Published:** 2023-03-15

**Authors:** Fumitaka Kudo, Takuji Chikuma, Mizuki Nambu, Taichi Chisuga, Shimpei Sumimoto, Arihiro Iwasaki, Kiyotake Suenaga, Akimasa Miyanaga, Tadashi Eguchi

**Affiliations:** † Department of Chemistry, Tokyo Institute of Technology, 2-12-1 O-okayama, Tokyo 152-8551, Japan; ‡ Department of Chemistry, Faculty of Science and Technology, Keio University, 3-14-1 Hiyoshi, Kohoku-ku, Yokohama, Kanagawa 223-8522, Japan

## Abstract

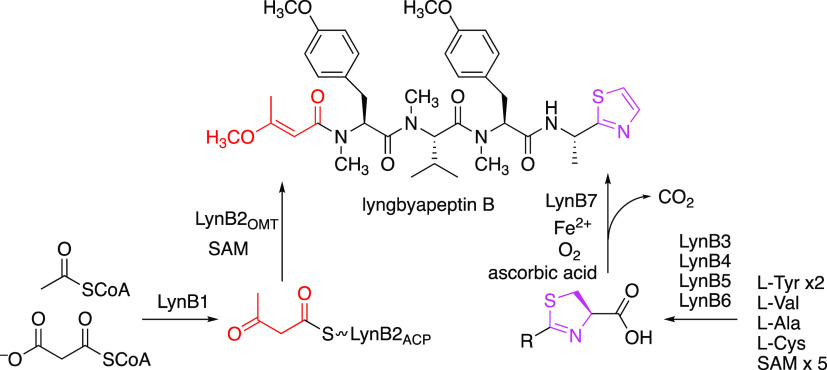

Lyngbyapeptin B is a hybrid polyketide-nonribosomal peptide isolated from particular marine cyanobacteria. In this report, we carried out genome sequence analysis of a producer cyanobacterium *Moorena bouillonii* to understand the biosynthetic mechanisms that generate the unique structural features of lyngbyapeptin B, including the (*E*)-3-methoxy-2-butenoyl starter unit and the C-terminal thiazole moiety. We identified a putative lyngbyapeptin B biosynthetic (*lynB*) gene cluster comprising nine open reading frames that include two polyketide synthases (PKSs: LynB1 and LynB2), four nonribosomal peptide synthetases (NRPSs: LynB3, LynB4, LynB5, and LynB6), a putative nonheme diiron oxygenase (LynB7), a type II thioesterase (LynB8), and a hypothetical protein (LynB9). In vitro enzymatic analysis of LynB2 with methyltransferase (MT) and acyl carrier protein (ACP) domains revealed that the LynB2 MT domain (LynB2-MT) catalyzes O-methylation of the acetoacetyl-LynB2 ACP domain (LynB2-ACP) to yield (*E*)-3-methoxy-2-butenoyl-LynB2-ACP. In addition, in vitro enzymatic analysis of LynB7 revealed that LynB7 catalyzes the oxidative decarboxylation of (4*R*)-2-methyl-2-thiazoline-4-carboxylic acid to yield 2-methylthiazole in the presence of Fe^2+^ and molecular oxygen. This result indicates that LynB7 is responsible for the last post-NRPS modification to give the C-terminal thiazole moiety in lyngbyapeptin B biosynthesis. Overall, we identified and characterized a new marine cyanobacterial hybrid PKS-NRPS biosynthetic gene cluster for lyngbyapeptin B production, revealing two unique enzymatic logics.

## Introduction

Marine cyanobacteria produce a variety of nonribosomal peptides (NRPs) and hybrid nonribosomal peptide-polyketides (NRP-PKs) with significant biological activity.^1−5^ A continuous exploration of natural products derived from marine cyanobacteria is thus expected to facilitate the discovery of new drug candidates. However, culturing marine cyanobacteria under laboratory conditions is often challenging, resulting in poor reproducibility of re-isolating marine cyanobacterial natural products. Therefore, total syntheses of natural products derived from marine cyanobacteria have been carried out extensively.^6−8^ In addition, performing biosynthetic studies, such as feeding experiments and biosynthetic gene knockout experiments, with marine cyanobacteria is difficult. Fortunately, genome sequence analysis with a small amount of genomic DNA derived from a single cell can provide information on biosynthetic genes coding for enzymes involved in target natural product synthesis.^9^ Because bacterial secondary metabolic genes are often clustered at a specific region on chromosomal DNA, the corresponding biosynthetic gene clusters (BGCs) can be easily identified using appropriate enzyme queries.^10^ In general, modular type I nonribosomal peptide synthetases (NRPSs) and polyketide synthases (PKSs) are responsible for the biosynthesis of various bacterial NRPs and hybrid NRP-PKs, which are readily identified by functional characterization of NRPS and PKS sequences as queries. In contrast, unique structural features of cyanobacterial NRPs and hybrid NRP-PKs are presumably introduced by unique enzymatic logics in addition to canonical NRPS and PKS logics.^10−13^ Unique catalytic domains are often inserted into NRPS and hybrid NRPS-PKS modules to modify building blocks for the extension of the peptide-polyketide chain or associated with NRPS-PKS BGCs to modify the peptide-polyketide backbone.

**Figure 1:**
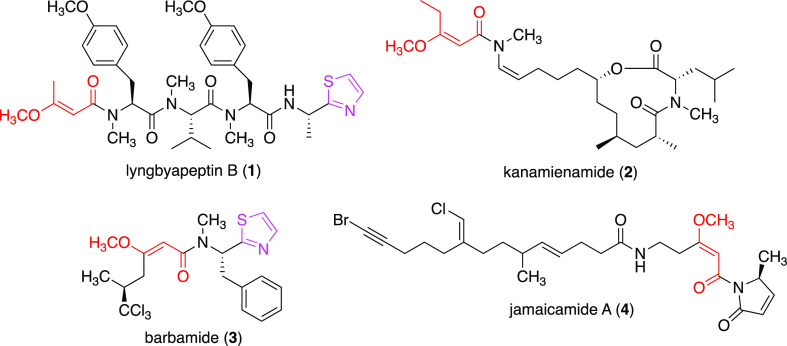
Structures of lyngbyapeptin B (**1**), kanamienamide (**2**), barbamide (**3**), and jamaicamide A (**4**). The methyl vinyl ether moieties are shown in red. The C-terminal thiazole moieties are shown in magenta.

In the present study, we focused on the formation of the methyl vinyl ether moiety in hybrid NRP-PK structures found in lyngbyapeptins **1**,^14−18^ kanamienamide **2**,^19^ barbamide **3**,^20^ and jamaicamides **4**(21) (Figure 1). The BGCs of barbamide^22^ and jamaicamide^21^ contain a “split” PKS module, which is separated between a β-ketoacyl:acyl carrier protein (ACP) synthase (KS)-acyltransferase (AT) didomain and a methyltransferase (MT)-ACP didomain that is postulated to be involved in O-methylation of the 3-oxoacyl-ACP intermediate. Here, we carried out genome mining of the biosynthetic gene cluster for lyngbyapeptin B and kanamienamide, which contain a methyl vinyl ether moiety at the starter unit of hybrid PK-NRP structures. We anticipated that the functional analysis of the enzymes responsible for the starter unit formation in lyngbyapeptin B and kanamienamide biosyntheses would be straightforward using 3-oxobutyryl-ACP or 3-oxopentanoyl-ACP as the substrate. Additionally, we have previously identified a marine cyanobacterium *Moorena bouillonii* (previously known as *Moorea bouillonii*) that produces lyngbyapeptin B^16^ and kanamienamide^19^ based on our natural product isolation studies. Consequently, we identified the lyngbyapeptin B BGC and characterized an O-methyltransferase, which is responsible for the (*E*)-3-methoxy-2-butenoyl starter unit formation in lyngbyapeptin B biosynthesis. Further, a unique oxidative decarboxylase responsible for C-terminal thiazole formation was enzymatically characterized. The combination of the methyl vinyl ether starter and the C-terminal thiazole ring is a common structural feature of lyngbyapeptins.^23^

## Results and Discussion

### Identification of *lynB* BGC

**Figure 2:**
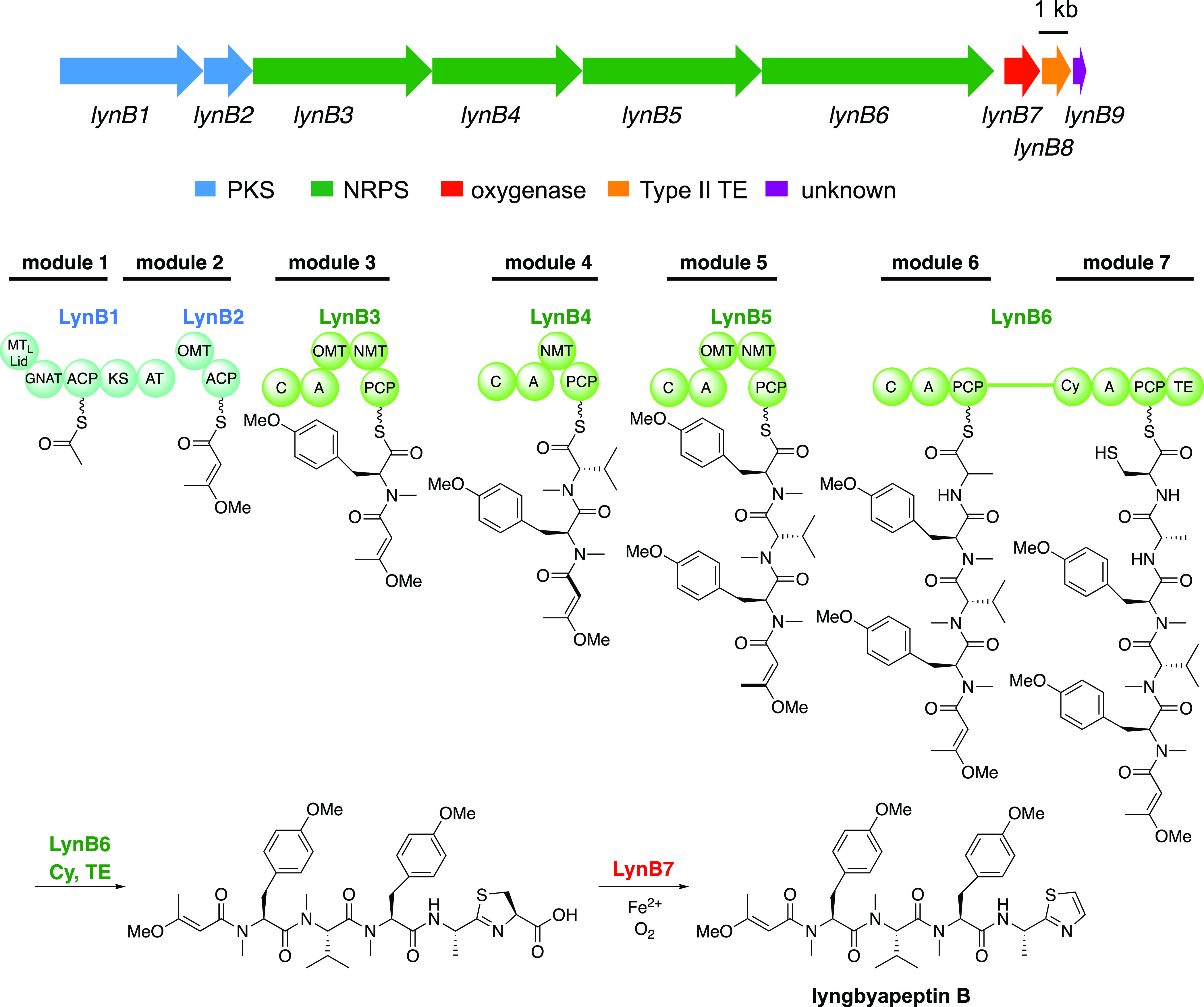
Biosynthetic gene cluster of lyngbyapeptin B (*lynB* BGC) and a proposed biosynthetic pathway. PKS, polyketide synthase; NRPS, nonribosomal peptide synthetase; TE, thioesterase; MT_L__Lid, minimal lid region of loading methyltransferase; GNAT, GCN5-related N-acetyltransferase; ACP, acyl carrier protein; KS, β-ketoacyl-ACP synthase; AT, acyltransferase; OMT, O-methyltransferase; C, condensation domain; A, adenylation domain; NMT, backbone N-methyltransferase; PCP, peptidyl carrier protein; Cy, cyclization domain.

Genome sequence analysis of the lyngbyapeptin B and kanamienamide-producing marine cyanobacterium *M. bouillonii* strain 1509-15, which was collected at the shore of Minna Island, Okinawa, Japan on 29 September 2015,^19^ was carried out by isolating single filaments using the pipet-washing method^24^ under a microscope, and whole genomic DNA was amplified using a filament as the template. The genome sequence analysis by PacBio RS II and subsequent assembly by SMRT Analysis ver. 2.3 with RS_HGAP_Assembly.3 gave a draft genome sequence of cyanobacterium *M. bouillonii* (Table S1). The 16S rRNA sequence in the draft genome sequence confirmed that the stain 1509-15 is *M. bouillonii*. More than 40% of marine cyanobacterial natural products have been isolated from the genus *Moorena* (previously known as *Moorea*).^25^ The GC content of *M. bouillonii* 1509-15 (43.5%) is similar to those of related cyanobacteria *M. producens* 3L (GC 43.7%), JHB (GC 43.7%), and *M. bouillonii* PNG (GC 43.7%).^26^ Among many fragmented NRPS, PKS, and hybrid NRPS-PKS genes, we found a hybrid NRPS-PKS gene cluster consisting of 33.5 kb, which was hypothesized to be responsible for lyngbyapeptin B biosynthesis based on the domain structure of the hybrid NRPS-PKS and was named the lyngbyapeptin B (*lynB*) BGC (accession number: LC514336; Figure 2 and Table S2).

**Scheme 1:**
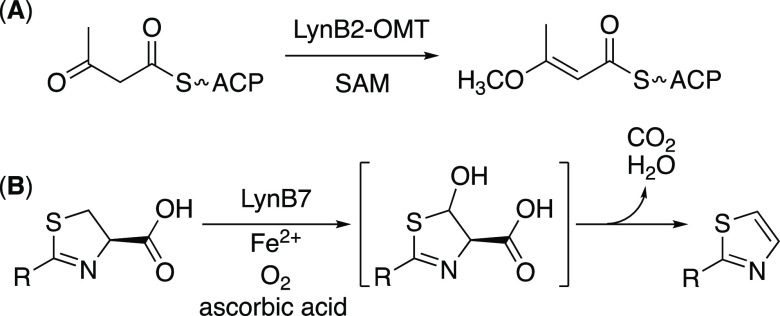
Characterized Enzymes in This Study (A) Formation of the methyl vinyl ether from acetoacetyl-ACP by an O-methyltransferase (OMT) domain, LynB2-OMT. (B) Proposed mechanism of C-terminal thiazole formation from 2-thiazoline-4-carboxylic acid via hydroxylation by nonheme diiron oxygenases, including LynB7 and BarH.

The putative starter PKS LynB1 consists of a minimal lid region of a loading methyltransferase^27^ (MT_L__Lid, formerly known as an adaptor region) domain, a GCN5-related N-acetyltransferase (GNAT) domain,^28^ and an ACP domain as module 1, which is linked to module 2 that consists of a KS domain and an AT domain. LynB1 likely interacts with LynB2, a split module comprising an MT-ACP didomain, which has the same domain structure as BarF (60.6% identity) in the barbamide BGC^22^ and JamN (66.5% identity) in the jamaicamide BGC.^21^ The AT domain of LynB1 (LynB1-AT) most likely recognizes malonyl-CoA, as predicted from its amino acid sequence,^29,30^ and transfers the malonyl group to both ACP domains of LynB1 (LynB1-ACP) and LynB2 (LynB2-ACP) to yield malonyl-LynB1-ACP and malonyl-LynB2-ACP, respectively. The GNAT domain of LynB1 (LynB1-GNAT) probably catalyzes the decarboxylation of malonyl-LynB1-ACP, as observed for other homologous GNAT domains, such as CurA in the curacin A BGC^31^ and GphF in the gephyronic acid BGC,^32^ to yield acetyl-LynB1-ACP.^27^ The formed acetyl-LynB1-ACP and malonyl-LynB2-ACP are then recognized by the KS domain of LynB1 (LynB1-KS) and condensed to yield acetoacetyl-LynB2-ACP. The MT domain on LynB2 (LynB2-OMT) is postulated to catalyze O-methylation of acetoacetyl-LynB2-ACP in the presence of *S*-adenosyl-l-methionine (SAM) to yield 3-methoxy-2-butenoyl-LynB2-ACP (Scheme 1A), which is then recognized by the condensation (C) domain of LynB3 (LynB3-C). The putative substrate specificity of adenylation (A) domains of NRPS modules LynB3, LynB4, LynB5, and LynB6 based on the 10-letter nonribosomal code^30,33,34^ agree well with the peptide structure of lyngbyapeptin B (Table S3). Thus, the A domain of LynB3 (LynB3-A) most likely activates L-Tyr and transfers this amino acid to the peptidyl carrier protein (PCP) domains of LynB3 (LynB3-PCP) to give L-Tyr-LynB3-PCP (Figure 2).

LynB3 also contains two methyltransferase (MT) domains which are postulated to O-methylate the phenol moiety of L-Tyr and N-methylate L-Tyr, respectively. These MT domains are located within the adenylation domain, like TioS^35^ in thiocoraline biosynthesis, PchF^36^ in pyochelin biosynthesis, and ColG^37^ in columbamide biosynthesis. Although the order of O-methylation and N-methylation is currently difficult to predict, both methylations appear to occur after condensation with the extended peptide intermediates, as observed for the methylation of the mature thiazolidine intermediate in the biosynthesis of pyochelin.^36^ However, LynB3 may catalyze N,O-dimethylation of l-tyrosyl-PCP before condensation because ColG that has two MT domains in the interrupted adenylation domain catalyzes N,O-dimethylation of L-seryl-PCP.^37^ The former MT domain in LynB3 (LynB3-OMT) is presumed to be O-methyltransferases of L-Tyr-OH because the MT domain shows a similarity to N-methyltransferases that methylate the side chain of l-arginine residues (approximately 30% similarities to arginine methyltransferases).^38−40^ The latter MT domain in LynB3 (LynB3-NMT) is similar to other backbone N-methyltransferases, including the MT domain of TioS (28% identity).^35^ LynB3-NMT is also similar to the MT domain of LynB4 (LynB4-NMT, 93% identity), which is presumably responsible for the N-methylation of the l-valine residue backbone. LynB5 comprises the same domain organization as that of LynB3 and likely uses the same mechanism. After the formation of 3-methoxy-2-butenoyl-*N*,*O*-diMe-L-Tyr-*N*-Me-L-Val-*N*,*O*-diMe-L-Tyr-LynB5-PCP, the last NRPS, LynB6, which contains two modules consisting of C-A(Ala)-PCP and Cy-A(Cys)-PCP-thioesterase (TE), attaches L-Ala and L-Cys. The C-terminal Cys residue of the elongated peptide intermediate is then cyclized to thiazoline by the cyclization (Cy) domain.^41−43^ Finally, the TE domain in LynB6 (LynB6-TE) hydrolyzes the thioester to yield a putative 2-thiazoline-4-carboxylic acid as the intermediate (Figure 2).

LynB7 shows similarity to the putative metallo-amidohydrolase BarH (77.1% identity) in barbamide biosynthesis^22^ and PtmU3 (26.9% identity) in platensimycin/platencin biosynthesis.^44^ PtmU3 has been characterized as a nonheme diiron monooxygenase that catalyzes the stereoselective hydroxylation at C-5 of diterpenoid-CoA intermediates using molecular oxygen and two divalent Fe cations at the active site.^44^ Therefore, LynB7 is hypothesized to be involved in the hydroxylation of the biosynthetic intermediate. One of the hypothesized functions of LynB7 would be a hydroxylase at C-5 of the thiazoline ring of the putative 2-thiazoline-4-carboxylic acid intermediate that is released by LynB6 (Scheme 1B). Subsequent dehydration and decarboxylation yield the thiazole ring to complete the biosynthesis of lyngbyapeptin B. Lyngbyapeptins and barbamides contain the C-terminal thiazole ring as a common structural feature. Therefore, the LynB7/BarH family of enzymes is considered responsible for putative oxidative decarboxylation.^22^ LynB8 may be a type II thioesterase that removes the undesired acyl and peptidyl intermediates from acyl-ACPs and/or peptidyl-PCPs for efficient PKS-NRPS elongation.^45^ LynB9 is a hypothetical protein containing DUF433, which is often found in cyanobacterial genomes and may be related to gene transfer.^46,47^ Consequently, the identified *lynB* biosynthetic gene cluster is probably responsible for the biosynthesis of lyngbyapeptin B in *M. bouillonii*. Unfortunately, identifying a likely kanamienamide BGC was not possible, presumably because the expected type I PKS gene-rich kanamienamide BGC was fragmented in genome sequence analysis.

### Characterization of LynB2 (OMT-ACP Didomain)

**Figure 3:**
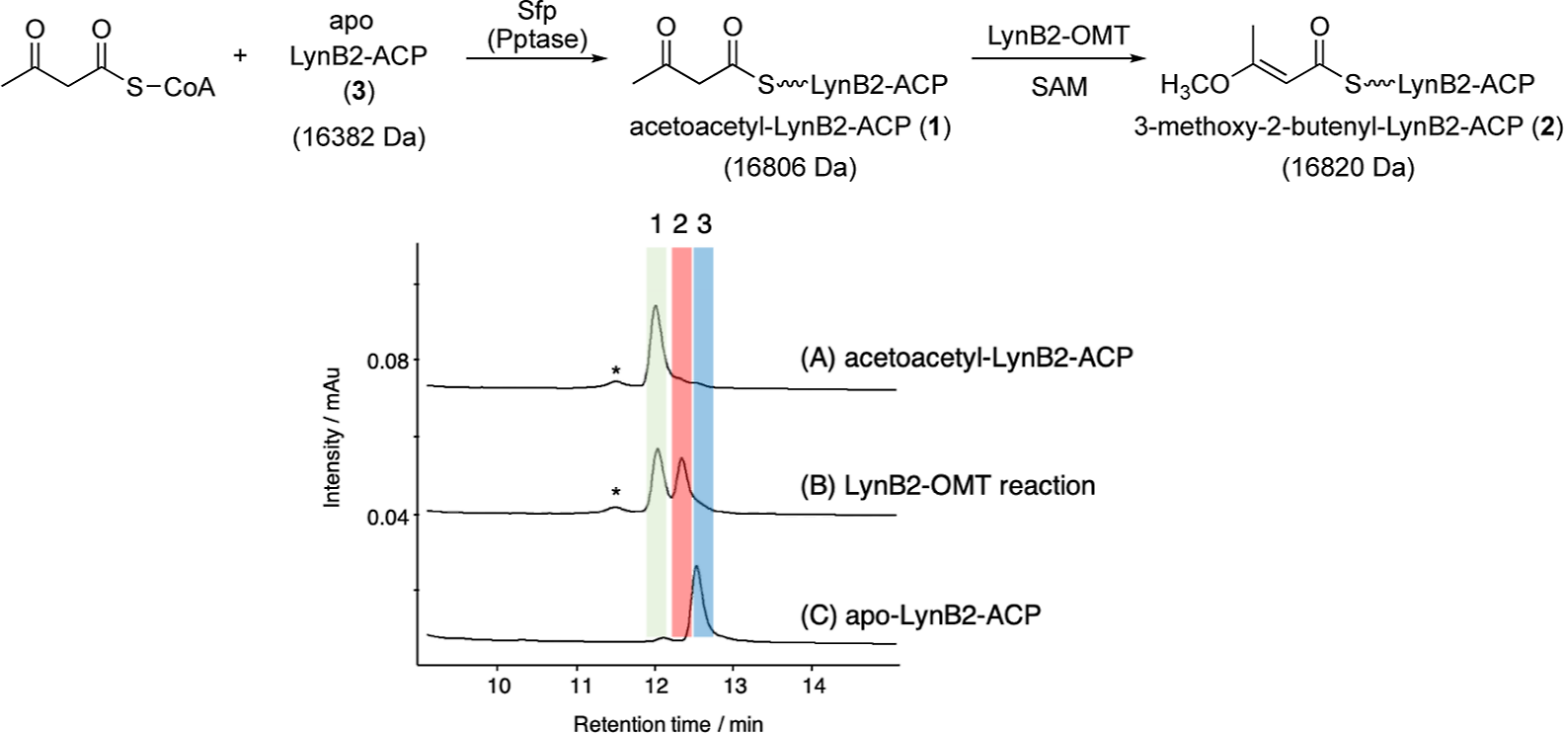
HPLC analysis of the LynB2-OMT reaction. (A) 100 μM LynB2-ACP, 500 μM acetoacetyl-CoA, and 2.5 μM Sfp, 1 h. (A,B) +50 μM LynB2-OMT + 2 mM SAM, 3 h. (C) 100 μM LynB2-ACP. Peak 1, acetoacetyl-LynB2-ACP; peak 2, 3-methoxy-2-butenyl-LynB2-ACP (LynB2-OMT reaction product); and peak 3, LynB2-ACP. The peaks with * are derived from Sfp.

The functional analysis of LynB2 consisting of the MT-ACP didomain was initially conducted to assess our biosynthetic hypothesis of lyngbyapeptin B (Scheme 1). In the present study, the MT domain of LynB2-OMT (1–362 of LynB2, 362 aa) and the ACP domain of LynB2-ACP (368–485 of LynB2, 118 aa) were expressed separately for enzymatic analysis (Figure S1). We used a codon-optimized artificial gene of the *lynB2* gene for expression in *Escherichia coli* (Table S4). Acetoacetyl-LynB2-ACP was prepared as a putative substrate from the apo form of LynB2-ACP with acetoacetyl-CoA using the phosphopantetheinyl transferase Sfp.^48^ After incubation with acetoacetyl-LynB2-ACP in the presence of SAM and recombinant LynB2-OMT, the resulting product was analyzed by HPLC and LC-ESI-MS. The expected methylated acetoacetyl-LynB2-ACP was detected (Figures 3 and S2). In contrast, neither acetoacetyl-CoA nor the N-acetylcysteamine thioester^49^ were methylated by LynB2-OMT under the same conditions (Figure S3). Therefore, the LynB2-ACP domain appears to be critical for the methylation activity of LynB2-OMT.

The methylated product from acetoacetyl-LynB2-ACP was hydrolyzed under basic conditions to off-load 3-methoxy-2-butenoic acid, which was then esterified with TMS-diazomethane. The obtained methyl ester was compared with authentic methyl (*E*)- and (*Z*)-3-methoxy-2-butenoate, which were chemically synthesized (Figures S4–S6).^50,51^ NMR data of the synthesized methyl (*E*)- and (*Z*)-3-methoxy-2-butenoate agreed well with the literature data.^52^ As a result of HPLC and LC-ESI-MS analysis, we found that the methylated product by LynB2-OMT was the expected (*E*)-3-methoxy-2-butenoate (Figure S7). We were concerned that the photoisomerization of the (Z)-isomer to the (E)-isomer may occur under visible light at room temperature during the enzymatic analysis because the (E)-isomer of 3-methoxy-2-butenoate is thermodynamically more stable than the (Z)-isomer, i.e., 9.2 kJ/mol for the (E)-isomer and 21.5 kJ/mol for the (Z)-isomer. Methyl (*Z*)-3-methoxy-2-butenoate has been prepared previously from the (E)-isomer by irradiating at 254 nm.^51^ However, we found that (*Z*)-methyl 3-methoxy-2-butenoate is not easily isomerized to the (E)-isomer by visible light at room temperature (Figure S6). Therefore, although LynB2-OMT may recognize the (*Z*)-enol form of 3-oxobutanoate and catalyze O-methylation to yield (*Z*)-3-methoxy-2-butenoate, photoisomerization of (*Z*)-3-methoxy-2-butenoate to yield the (E)-isomer is unlikely. Overall, we clarified that LynB2-OMT recognizes acetoacetyl-LynB2-ACP and catalyzes O-methylation to yield (*E*)-3-methoxy-2-butenyl-LynB2-ACP (Scheme 1A). The C domain of LynB3-C likely recognizes (*E*)-3-methoxy-2-butenoate and excludes 3-oxobutanoate as a starter unit for the subsequent NRPS reaction. Thus, the C domain of LynB3 functions as a gatekeeper to select an appropriate polyketide starter unit to initiate the hybrid PKS-NRPS assembly line. However, previous heterologous expression of the barbamide BGC in *Streptomyces venezuelae* has yielded 4-*O*-demethylbarbamide, indicating that the adjacent C domain accepts the 3-oxoacyl-ACP intermediate without O-methylation.^53^ It is unclear why the MT domain of BarF does not function in *S. venezuelae*.

A model structure of LynB2-OMT (Figure S8) was constructed by AlphaFold2^54^ and compared with the crystal structures of StiE-MT (PDB entry: 6ecx, 33% identity) and StiD-MT (PDB entry: 6ecu, 30% identity), which catalyze O-methylation of (*S*)- and (*R*)-β-hydroxy groups of polyketide intermediates, respectively, in stigmatellin biosynthesis by myxobacterium *Stigmatella aurantiaca*.^55^ The overall structure and the active site architecture of LynB2-OMT are similar to those of StiE-MT and StiD-MT (Figures S8 and S9). Residues Y45, Y46, L49, F62, Y121, H190, and Y306 of LynB2-OMT form a binding pocket for SAM and substrate. A catalytically important Glu residue in StiE-MT and StiD-MT is replaced with a Gln residue in the LynB2/BarF/JamN protein family that catalyzes O-methylation of the 3-oxoacyl-ACP intermediate. MtaF in myxothiazole A biosynthesis^56^ and CtaF in cystothiazole A biosynthesis^57^ are typical multi-domain type I PKSs comprising KS-AT-MT-ACP and MT domains (MtaF-MT and CtaF-MT) and are postulated to be responsible for O-methylation of 3-oxoacyl-ACP intermediates to yield methyl vinyl ether intermediates. MtaF-MT and CtaF-MT show similarity to proteins in the LynB2/BarF/JamN family (approximately 40% identities), including the Gln residue (Figure S9). MtaE in myxothiazole A biosynthesis and CtaE in cystothiazole A biosynthesis are type I PKSs composed of KS-AT-MT-β-ketoreductase (KR)-ACP. The MT domains (MtaE-MT and CtaE-MT) are postulated to *O*-methylate 3-hydroxyacyl-ACP intermediates and show similarity to proteins of the StiD-MT/StiE-MT family (40–50% identities), including the catalytically important Glu residue. The catalytic Glu residue in StiE-MT and StiD-MT likely functions as a base for deprotonation of the β-hydroxy groups of substrates. In contrast, O-methylation of the β-oxo group in 3-oxoacyl-ACP intermediates does not require such a deprotonation step. Thus, the Gln186 residue of LynB2-OMT may adjust the direction of the β-oxo group of substrates toward the electrophilic methyl group of SAM (Figure S10). Structural analysis of LynB2-OMT with appropriate ligands, along with mutational analysis, represents the next research goal in characterizing the substrate recognition and reaction mechanism of this enzyme. The stand-alone-type methyltransferases, LymB^58^ and Str2,^59^ which are responsible for the O-methylation of aldehyde intermediates to yield methyl vinyl ethers in lymphostin and strobilurin A biosynthesis, show low similarity to the MT domains of PKSs, suggesting that the reaction mechanism and substrate recognition mechanism differ.

### Characterization of LynB7 (Nonheme Diiron Oxygenase/Decarboxylase)

**Figure 4:**
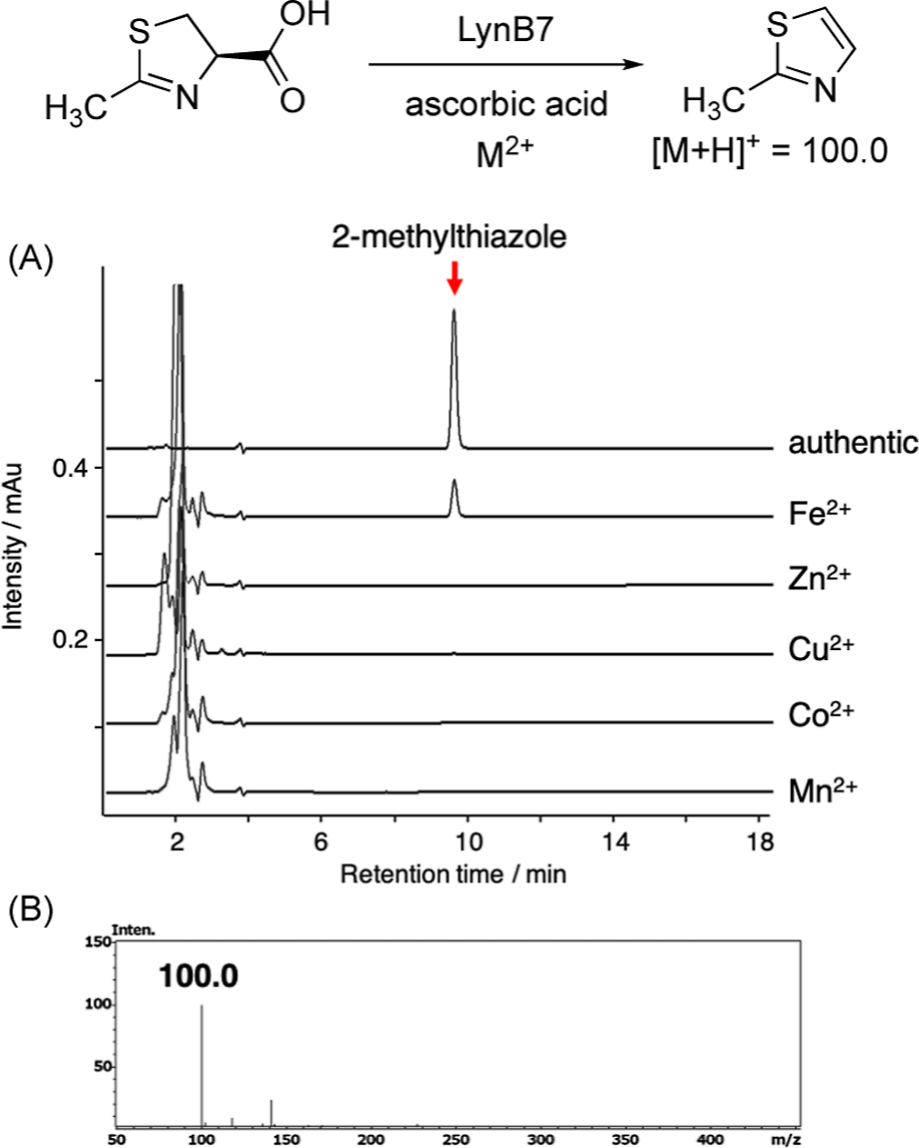
HPLC analysis of the LynB7 reaction with (4*R*)-2-methyl-2-thiazoline-4-carboxylic acid. (A) LynB7 reaction with 2 mM (4*R*)-2-methyl-2-thiazoline-4-carboxylic acid and 50 μM LynB7 in the presence of 1 mM Fe^2+^, Zn^2+^, Cu^2+^, Co^2+^, or Mn^2+^. (B) Mass spectrum of 2-methylthiazole produced by LynB7.

Next, to investigate the hypothesized function of LynB7, the codon-optimized *lynB7* gene was expressed in *E. coli*, and the recombinant LynB7 protein was purified for enzymatic analysis (Figure S1). In this study, (4*R*)-2-methyl-2-thiazoline-4-carboxylic acids were prepared from l-cysteine with acetonitrile to mimic the putative biosynthetic intermediate (Figures 4 and S11).^60^ Several divalent cations including Fe^2+^, Zn^2+^, Cu^2+^, Co^2+^, or Mn^2+^ were added to the enzymatic solution because LynB7 appears to require certain metal cations for enzymatic activity. After the enzymatic reaction, the product was extracted with ethyl acetate and analyzed by HPLC and LC-ESI-MS. In the presence of Fe^2+^, the LynB7 reaction afforded 2-methylthiazole from (4*R*)-2-methyl-2-thiazoline-4-carboxylic acid (Figure 4), whereas a small amount of 2-methylthiazole was generated from (4*S*)-2-methyl-2-thiazoline-4-carboxylic acid (Figure S12). The *K*_m_ values for (4*R*)- and (4*S*)-2-methyl-2-thiazoline-4-carboxylic acids were 2.1 ± 0.2 mM and 3.6 ± 0.7 mM, respectively (Figure S13). The *k*_cat_ values for (4*R*)- and (4*S*)-2-methyl-2-thiazoline-4-carboxylic acids were 17 ± 0.7 and 1.3 ± 0.1 s^–1^, respectively. Stereochemistry at the C4 position affects the catalytic efficiency rather than recognition of the substrates. Therefore, putative stereoselective hydroxylation at C5 of the substrate may be critical for efficient dehydration and subsequent decarboxylation to yield thiazole (Scheme 1B).

Testing (4*R*)-2-phenyl-2-thiazoline-4-carboxylic acid as a substrate of LynB7 gave oxidative decarboxylation to yield 2-phenylthiazole (Figures S14 and S15). The kinetic parameters for (4*R*)-2-phenyl-2-thiazoline-4-carboxylic acid were estimated to be *K*_m_ = 1.5 ± 0.3 mM and *k*_cat_ = 11 ± 0.5 s^–1^ (Figure S13). Therefore, LynB7 has tolerant substrate specificity toward substituents at the C2 position of 2-thiazoline-4-carboxylic acid substrates. In contrast, LynB7 did not afford any new product with (4*R*)-2-phenyl-2-thiazoline-4-carboxamide (Figures S16 and S17). Therefore, the 4-carboxylic acid moiety of 2-thiazoline-4-carboxylic acid substrates is critical for the reaction. In addition, 2-phenyl-2-thiazole-4-carboxylic acid was unchanged by LynB7 (Figure S17), indicating that the methylene moiety of 2-alkyl-2-thiazoline-4-carboxylic acid substrates is critical for LynB7 enzymatic activity. We then conducted the LynB7 reaction with (4*R*)-[5,5-^2^H_2_]-2-phenyl-2-thiazoline-4-carboxylic acid and compared the initial velocity with that of the nondeuterated substrate (Figures S18 and S19). As a result, the primary deuterium kinetic isotope effect (KIE) in the LynB7-catalyzed reaction was estimated to be 5.9 based on the production rate of 2-phenylthiazole. The observed KIE with (4*R*)-[5,5-^2^H_2_]-2-phenyl-2-thiazoline-4-carboxylic acid indicates that one of the pro-chiral hydrogen atoms at C5 of the 2-thiazoline-4-carboxylic acid is selectively abstracted to yield a radical intermediate, which then reacts with putative iron-oxo species at the active site to afford the hydroxylated product with the retention of the stereochemistry (Figure S20A). The trans orientation of the hydroxy and carboxylate groups on the thiazoline ring should favor efficient β-elimination, which is assisted by decarboxylation. The iron atom at the active site of LynB7 may also assist dehydration as a Lewis acid, whereas the generated radical at C5 of the substrate may trigger β-cleavage to yield the thiazole product without hydroxylation (Figure S20B). Detailed biochemical analysis of this unique oxidative decarboxylase is needed to understand the precise reaction mechanism.

The model structure of LynB7 (Figure S21) was constructed by AlphaFold2^54^ and compared with the crystal structures of PtmU3 (PDB entries: 6omp and 6omr).^44^ The overall structure and the active site architecture of LynB7 are similar to those of PtmU3. Three conserved His residues (H13, H197, and H323), two Asp residues (D11 and D320), and one Glu residue (E252) in LynB7 coordinate with two divalent metal cations at the active site (Figures S21 and S22). E313 in PtmU3 is replaced with Asp (D325) in proteins of the LynB7/BarH family. D325 in LynB7 may coordinate with the metal cation in the resting state of LynB7 to maintain the protein conformation. Nonheme diiron monooxygenases are an emerging family of oxygenases, one of which catalyzes hydroxylation and N-oxygenation in chloramphenicol biosynthesis.^61^ Mechanisms of dioxygen activation by nonheme diiron oxygenase have been studied extensively,^62^ whereas the nonheme iron oxygenase reaction mechanisms of proteins belonging to the LynB7/BarH family remain unresolved. The characterized function of LynB7 herein as an oxidative decarboxylase paves the way for future mechanistic analysis. Protein structural analysis of LynB7 with appropriate ligands and mutational analysis will be carried out to understand the substrate recognition and reaction mechanism of this unique family of diiron oxidative decarboxylases.

## Conclusions

We identified the putative lyngbyapeptin B biosynthetic (*lynB*) gene cluster from the genome of the cyanobacterium *M. bouillonii* and characterized the O-methyltransferase domain in the split PKS module LynB2 to yield (*E*)-3-methoxybut-2-enoate as a unique polyketide starter that is linked to the NRPS assembly line. It remains unclear why this type of PKS module is often split between the AT and MT domains. Presumably, after Claisen condensation by the KS domain, the 3-oxobutyryl-MT-ACP didomain may dissociate from the KS domain to facilitate efficient O-methylation by the MT domain. Investigating how these catalytic PKS domains interact with the acyl-ACP domain represents a future topic to address. An appropriate protein–protein interaction between PKS-NRPS modules is important for rational PKS-NRPS engineering.^63^ Combinational protein–protein interactions between docking domains of PKS/NRPSs have been reported in the combinatorial biosynthesis of vatiamides.^64^ Although the putative docking motifs of LynB PKS/NRPSs are shown in Figure S23, it is unclear how these putative docking domains interact with each other for efficient acyl transfers. Carrying out functional and structural analysis of the putative docking domains of the hybrid PKS-NRPSs of LynB proteins represents a potential future project. In this report, we have further characterized a unique nonheme diiron oxygenase LynB7, which catalyzes the oxidative decarboxylation of 2-thiazoline-4-carboxylic acid to yield 2-thiazole. Notably, LynB7 differs from well-characterized thiazole-forming enzymes, including the flavin mononucleotide (FMN)-dependent dehydrogenase McbC in microcin 17 biosynthesis,^65^ FMN-dependent oxidase domain of EpoB in epothilone biosynthesis,^41^ FMN-dependent oxidase domain of BlmIII in bleomycin biosynthesis,^66^ and cytochrome P450 enzyme BtmJ in bottromycin A_2_ biosynthesis.^67^ Moreover, LynB7 shows low similarity to the amidohydrolase PurAH (14% identity), which is a thiazoline-specific amidohydrolase in the presence of Co^2+^ and Zn^2+^ in bottromycin biosynthesis.^68^ Therefore, the currently characterized enzymes involved in a marine cyanobacterial hybrid PK-NRP biosynthesis have provided new unique enzymatic tools in the initiation and termination reactions. Future structural analysis of these enzymes will enhance our knowledge and enable the identification of new natural product BGCs, leading to the discovery of new natural products.

## Experimental Section

For experimental details, see the Supporting Information.

## Supporting Information Available

Experimental procedures, summary of draft genome sequence analysis, table of lyngbyapeptin B (*lynB*) biosynthetic genes, table of nonribosomal codes of adenylation enzymes, sequences of artificial genes for *lynB2* and *lynB7*, SDS-PAGE analysis of the expressed proteins, NMR spectra of synthetic compounds, LC-ESI-MS analysis of the enzyme reaction products, kinetic analysis of LynB7, model structures of LynB2-OMT and LynB7, sequence alignments of the LynB2-OMT and LynB7 family proteins, and proposed reaction mechanisms of LynB2-OMT and LynB7 (PDF)

## Accession Codes

lynB BGC (LC514336).

## Abbreviations

A: adenylation domain
ACP: acyl carrier protein
AR: adaptor domain
AT: acyltransferase
BGC: biosynthetic gene cluster
C: condensation domain
CoA: coenzyme A
Cy: cyclization domain
GNAT: GCN5-related *N*-acetyltransferase
HPLC: high-performance liquid chromatography
KIE: kinetic isotope effect
KS: β-ketoacyl:acyl carrier protein synthase
LC-ESI-MS: liquid chromatography-electrospray ionization-mass spectrograph
M: *Moorena*
MT: methyltransferase
NMT: N-methyltransferase
NRP: nonribosomal peptide
NRPS: nonribosomal peptide synthetase
NRP-PK: nonribosomal peptide-polyketide
OMT: O-methyltransferase
PCP: peptidyl carrier protein
PKS: polyketide synthase
SAM: *S*-adenosyl-l-methionine
TE: thioesterase
