# Supplementary material for: Unique Initiation and Termination Mechanisms Involved in the Biosynthesis of a Hybrid Polyketide-Nonribosomal Peptide Lyngbyapeptin B Produced by the Marine Cyanobacterium Moorena bouillonii
Source: ACS Chem Biol. 2023 Mar 15;18(4):875–83. doi: 10.1021/acschembio.3c00011 (PMC10127204; doi:10.1021/acschembio.3c00011)
Supplement: Supplementary file 1 — cb3c00011_si_001.pdf [file cb3c00011_si_001.pdf]

Supporting Information

Unique Initiation and Termination Mechanisms Involved in the Biosynthesis of a Hybrid Polyketide-nonribosomal Peptide Lyngbyapeptin B Produced by the Marine Cyanobacterium *Moorena bouillonii*

Fumitaka Kudo*†, Takuji Chikuma†, Mizuki Nambu†, Taichi Chisuga†, Shimpei Sumimoto‡, Arihiro Iwasaki‡, Kiyotake Suenaga‡, Akimasa Miyanaga†, Tadashi Eguchi*†

†Department of Chemistry, Tokyo Institute of Technology, 2-12-1 O-okayama, Tokyo 152-8551, Japan.
‡Department of Chemistry, Faculty of Science and Technology, Keio University, 3-14-1 Hiyoshi, Kohoku-ku, Yokohama, Kanagawa 223-8522, Japan.

Email: fkudo@chem.titech.ac.jp, eguchi@chem.titech.ac.jp

Experimental

Collection and isolation of marine cyanobacterium

The marine cyanobacterium *Moorena bouillonii* strain 1509-15 was collected at shore of Minna Island in Okinawa Prefecture, Japan, in September 2015. Fresh sample was used to whole genome amplification. To remove the cyanobacterial sheaths and heterotrophic contaminant bacteria, small pieces of collected *M. bouillonii* were added to 500 µL of 0.1% (w/v) sodium *N*-lauroylsarcosinate (Nacalai Tesque Inc., Japan) in TES buffer [10 mM Tris (pH 8.0), 50 mM EDTA and 50 mM NaCl in distilled water]. The suspension was mixed and incubated for 10 min at room temperature. Supernatant of the solution was transferred to a 1.5 mL plastic microtube and centrifuged at 13,000 rpm for 30 sec. The supernatant was removed, and the pellets were suspended in 100 µL of TES buffer by pipetting. After centrifuged at 13,000 rpm for 30 sec, the supernatant was removed and the pellets were suspended in 100 µL of TES buffer, which contained 10 µg/mL of Proteinase K and 0.1% (w/v) of SDS. The suspension was incubated for 2 h at 57 ˚C. Then, single filament of *M. bouillonii* was separated by pipette-washing method[1] under a light microscope using a sterilized slide glass. The solution containing the aggregated filaments of *M. bouillonii* (ca. 10 µL) was added to one droplet of sterilized seawater placed on a slide glass. Then, single filament of *M. bouillonii* was picked up and transferred to another droplet using a micropipette prepared from Pasteur pipette. This process was repeated until bacterial contamination was eliminated to obtain pure single filament.

Whole genome amplification

All 1.5 mL plastic microtubes were sterilized and exposed UV for over-night before using for whole genome amplification. The isolated single filament was crushed with a pipette on a slide glass under a light microscope.

The crushed filament was dissolved in 1.5 μL of sterilized TE buffer and transferred to a 0.2 mL plastic microtube. The sterilized 1.5 μL TE buffer containing the broken single filament was used as DNA template for whole genome amplification (Repli-g UltraFast Mini Kit, Qiagen), according to the kit manufacturer's protocol. Whole genome amplification was performed in three tubes separately. Amplified three g-DNA samples were combined to yield approximately 7 μg and used for genome sequencing.

Draft genome sequence analysis of *Moorena bouillonii* strain 1509-15

The genome sequence of *Moorena bouillonii* strain 1509-15 was analyzed with PacBio RS II by Takara Bio. The amplified g-DNA was used to prepare a template with PacBio DNA Template Prep Kit 1.0, PacBio DNA/Polymerase Binding Kit P6 v2, and PacBio MagBead Kit using PacBio Binding Calculator Version 2.3.1.1. The small fragments lower than 20 kb of SMRTbell template was removed using Blue Pippin (Sage Science). PacBio RS II sequencing was conducted with PacBio DNA Sequencing Reagent 4.0, PacBio SMRT Cell 8 Pac V3, and PacBio MagBead OneCellPerWell. The raw data were treated with SMRT Analysis v2.3.0 to give subreads; the longer reads were used as seed reads and the shorter reads were mapped onto the seed reads to obtain pre-assembled reads. Pre-assembled reads were assembled with Celera Assembler in HGAP (Hierarchical Genome Assembly Process) to obtain a draft contig. Subreads were mapped again toward the draft contigs with Quiver in SMRT Analysis to yield a draft genome sequence (**Table S1**). The resulting genome sequence was analyzed with the MiGAP annotation tool provided by DNA Data Bank of Japan (DDBJ) and AntiSMASH. Furthermore, the putative lyngbyapeptin B (lynB) BGC was manually checked and annotated by comparison with homologous proteins using BLAST analysis. The resultant lynB BGC (33,481 bps) was deposited in the DDBJ database under accession number LC514336 (**Table S2**).

Expression and purification of LynB2-OMT, LynB2-ACP, and LynB7 (Figure S1)

The codon-optimized artificial gene (**Table S4**) for LynB2 to express in *Escherichia coli* was designed and synthesized by Eurofin Genetics (Japan). The synthesized *lynB2* gene in a plasmid pEX-A2J1 (*lynB2*/pEX-A2J1) was digested with *Nde*I and *Hin*dIII. The resultant DNA fragment of the *lynB2* gene was inserted into the corresponding restriction site of pET30a to yield *lynB2*/pET30a. The *lynB2-MT* and *lynB2-ACP* genes were amplified with the *lynB2*/pET30a plasmid as PCR template. Primer sets of LynB2_MT-R (5'-TT<u>GGATCC</u>TCAGACGACATTGCTTTCTTC-3') and T7 promoter primer (5'-TAATACGACTCACTATAGGG-3') and LynB2_ACP-F (5'-AGAAAAG<u>CATATG</u>AACTTCCGTGAACAGTTT-3') and T7 terminator primer (5'-ATGCTAGTTATTGCTCAGCGG-3') were used to amplify the *lynB2-MT* gene and the *lynB2-ACP* gene, respectively. The introduced restriction sites are underlined. PCR was conducted with PrimeSTAR GXL DNA Polymerase (Takara Bio) according to the manufacturer's instructions. Each PCR solution consisted of 2 μl of 5 x PrimeSTAR GXL buffer, 0.8 μl of dNTP mix (2.5 mM), 0.3 μl of each primer (10 μM), 0.3 μl of *lynB2*/pET30a, 0.2 μl of PrimeSTAR GXL DNA Polymerase, and sterilized water to 10 μl. The PCR

conditions were 95 ºC, 10 sec; 30 cycles of 98 ºC, 10 sec; 60 ºC, 30 sec; 68 ºC, 60 sec. The amplified DNA solutions were separated with 0.7% agarose electrophoresis with 1 x TAE buffer. The DNA fragments of desired size were recovered with the gene gel/PCR extraction kit (NIPPON Genetics Co., Ltd.) according to the manufacturer's instructions. Typically, 20 µL of solution of each DNA fragment was obtained. The PCR products were digested with *Nde*I/*Bam*HI and *Nde*I/*Hin*dIII, respectively and the resultant DNA fragments were inserted into the corresponding pET28a or pColdI to obtain *lynB2-MT/pET28a* and *lynB2-ACP/pColdI* for expression. The *lynB2-MT/pET28a* and *lynB2-ACP/pColdI* were introduced to *E. coli* BL21(DE3) to obtain the expression strains (*lynB2-MT*/pET28a/BL21 and *lynB2-ACP*/pColdI/BL21).

The *lynB2-MT*/pET28a/BL21 strain was grown in LB media containing 30 µg/mL of kanamycin at 37 ºC, 200 rpm until OD_{600} became approximately 0.6 and final 0.2 mM of isopropyl-β-*D*-1-thiogaractopyranoside (IPTG) was added into the culture to induce the expression. The culture was continued for 18 h at 15 ºC. The cells were collected by centrifugation and suspended in a buffer A (50 mM HEPES-NaOH (pH 7.5) containing 10% glycerol) with 300 mM NaCl. The cell suspension was homogenized by sonication on ice to yield the cell free extract containing the LynB2-OMT protein. After centrifugation at 10,000 rpm and 4 ºC for 15 min, the supernatant was loaded onto TARON® Metal Affinity Resin and washed with buffer A containing 20 mM imidazole and 300 mM NaCl. The adsorbed LynB2-OMT was eluted with buffer A containing 200 mM imidazole and 300 mM NaCl. The solution containing LynB2-OMT was passed through PD-10 (Cytiva) to remove imidazole. The LynB2-OMT containing solution was concentrated with centrifugal contractors (Amicon Ultra-15 and Amicon Ultra-4, 10 kDa MWCO, Merck) by centrifugation (4,900 × *g*, at 4 ºC, 20 min for several times). LynB2-OMT was fused with N-terminus His6 derived pET28 vector and the size of LynB2-MT-N-His (LynB2-OMT) is 381 aa and 43.7 kDa (without N-terminal Met).

The expression of LynB2-ACP was carried out according to the same method for LynB2-OMT except for antibiotics (50 µg/mL of ampicillin) in culture conditions. The purification of LynB2-ACP was carried out according to the same method for LynB2-OMT except for centrifugal contractors (Amicon Ultra-15 and Amicon Ultra-4, 10 kDa MWCO). LynB2-ACP with the pColdI vector was fused with N-terminus His6 and the size of LynB2-ACP-N-His (LynB2-ACP) is 142 aa and 16,382 Da (without N-terminal Met).

The codon-optimized artificial gene (**Table S4**) for LynB7 to express in *E. coli* was designed and synthesized by Eurofin Genetics (Japan). The synthesized *lynB7* gene in a plasmid pEX-A2J1 (*lynB7*/pEX-A2J1) was digested with *Nde*I and *Xho*I. The resultant DNA fragment of the *lynB7* gene was inserted into the corresponding restriction site of pColdI to yield *lynB7*/pColdI. The *lynB7/pColdI* was introduced to *E. coli* BL21(DE3) to obtain the expression strains (*lynB7*/pColdI/BL21). The expression and purification of LynB7 was carried out according to the same method for LynB2-ACP. For purification of LynB7, buffer B (50 mM HEPES-NaOH (pH 8.0) containing 10% glycerol) and His60 Ni Superflow Resin were used. The LynB7 containing solution was concentrated with centrifugal contractors (Amicon Ultra-15 and Amicon Ultra-4, 10 kDa MWCO) by centrifugation (4,900 × *g*, at 4 ºC, 20 min for several times). LynB7 with the pColdI vector

was fused with N-terminus His6 and the size of LynB7-N-His (LynB7) is 378 aa and 44.3 kDa (without N-terminal Met).

Enzymatic analysis of LynB2-OMT

LynB2-OMT reaction with acetoacetyl-LynB2-ACP (3-oxobutanoyl-LynB2-ACP)

A reaction mixture consisting of 100 μM LynB2-ACP, 500 μM acetoacetyl-CoA (Sigma-Aldrich), 2.5 μM Sfp,[2, 3] 22.5 mM $MgCl_2$ in the 50 mM HEPES-NaOH (pH 7.5) buffer containing 10% glycerol and 300 mM NaCl were incubated at 28 ºC for 1 h. The solution was then mixed with 1 mM SAM and 50 μM LynB2-OMT (total 100 μL) and further incubated at 28 ºC for 3 h.

HPLC analysis was performed with a HPLC system (Hitachi ELITE LaChrom L-2130 Pump, L-2455 Diode Array Detector, SSC-2300 Column Oven, and SSC-3215 Degasser) with a COMOSIL $5C_4$-AR300 (4.6 ø × 250 mm; Nacalai Tesque Inc., Japan); 10 μL of solution was injected into the HPLC system. The mobile phase was a two-step linear gradient of 0.1% TFA and acetonitrile with 0.1% (v/v) TFA (35–60% (v/v) acetonitrile for 10 min and 60–62.5% (v/v) acetonitrile for 20 min) with a flow-rate of 1.0 mL/min at 28 °C. For the next analytical cycle, the column was washed with 90% (v/v) acetonitrile for 10 min. Elution was monitored at 280 nm. To isolate the methylated product, a linear gradient of 0.1% TFA and acetonitrile with 0.1% (v/v) TFA (50–60% (v/v) acetonitrile for 10 min) was used. The fractionated solution containing the methylated product was evaporated to remove acetonitrile and then lyophilized to obtain the sample for LC-ESI-MS analysis.

LC-ESI-MS analysis was performed with an LC-MS system (Shimadzu LCMS-2020, LC-20AD pump, and SPD-M20A detector) with a COMOSIL Protein-R (2.0 ø × 150 mm; Nacalai Tesque Inc., Japan); 3 μL of solution was injected into the LC-MS system. The mobile phase was a two-step linear gradient of 0.1% formic acid and acetonitrile with 0.1% (v/v) formic acid (35–50% (v/v) acetonitrile for 10 min and 50–60% (v/v) acetonitrile for 30 min) with a flow-rate of 0.1 mL/min for acetoacetyl-ACP and 0.2 mL/min for methylated product at 40 °C. For the next analytical cycle, the column was washed with 90% (v/v) acetonitrile for 10 min. Elution was monitored at 280 nm.

Off-load of 3-methoxy-2-butenoate from methylated acetoacetyl-LynB2-ACP by LynB2-OMT

A reaction mixture consisting of 300 μM LynB2-ACP, 1.5 mM acetoacetyl-CoA, 7.5 μM Sfp, 22.5 mM $MgCl_2$ in the 50 mM HEPES-NaOH (pH 7.5) buffer containing 10% glycerol and 300 mM NaCl were incubated at 28 ºC for 1 h. The solution was then mixed with 3 mM SAM and 150 μM LynB2-OMT (total 1.5 mL) and further incubated at 28 ºC for 3 h. The solution was passed through PD-10 to remove glycerol using with 50 mM HEPES-NaOH (pH 7.5) containing 300 mM NaCl. The solution was concentrated with centrifugal contractors (Amicon Ultra-4, 10 kDa MWCO) by centrifugation (4,900 × g, at 4 ºC, 20 min). The solution was mixed with 0.5 M KOH and incubated at 60 ºC for 30 min. The resultant solution was neutralized with 2 M HCl and concentrated in vacuo to give the crude residue, which was dissolved in 100 μL of a solution of

CH_2Cl_2/CH_3OH (7:3). The solution was reacted with 2 M TMS diazomethane in hexane at 28 ºC for 30 min to yield methyl 3-methoxy-2-butenoate.

HPLC analysis was performed with a HPLC system (Hitachi Chromastar 5110 Pump, 5310 Column Oven, 5430 Diode Array Detector, and SSC-3215 Degasser) with a ODS 100Z (4.6 ø × 250 mm; TOSOH, Japan); 10 µL of the solution was injected into the HPLC system. The mobile phase was a linear gradient of water and CH_3OH (40–70% (v/v) CH_3OH for 20 min) with a flow-rate of 1.0 mL/min at 40 °C. Elution was monitored at 240 nm. For the next analytical cycle, the column was washed with 90% (v/v) CH_3OH for 10 min. LC-APCI-MS analysis was performed with the above-mentioned LC-MS system with an ODS-100Z (2.0 ø × 150 mm); An aliquot (2 µL) of solution was injected into the system. The mobile phase was a linear gradient of water and CH_3OH (40–70% (v/v) CH_3OH for 20 min) with a flow-rate of 0.5 mL/min at 40 °C. Elution was monitored at 240 nm.

Synthesis of methyl (*E*)- and (*Z*)-3-methoxy-2-butenoate as authentic compound

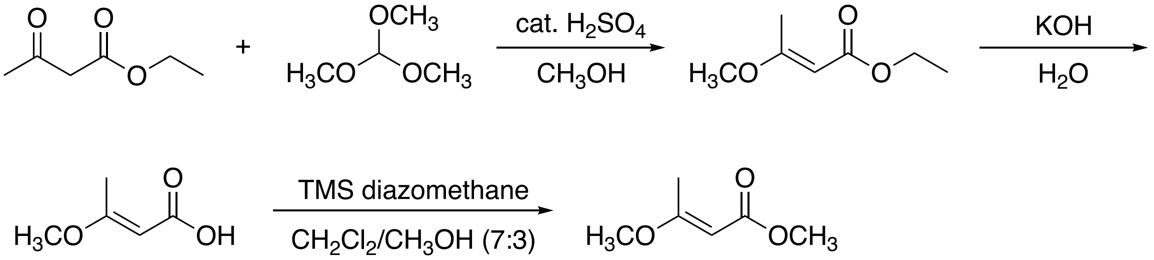

Under an argon atmosphere, two drops (approximately 100 µL) of conc. H_2SO_4 were added to a solution of ethyl acetoacetate (2.5 mL, 20 mmol) and trimethyl orthoformate (2.1 mL, 20 mmol) in super dehydrated methanol (2 mL). The mixture was stirred at room temperature overnight. The solution was neutralized with sat. $NaHCO_3$ and concentrated in vacuo to give the crude residue, which was purified by silica-gel chromatography (hexane:ethyl acetate = 9:1) to yield ethyl (*E*)-3-methoxy-2-butenoate (615 mg, 21%). ^{1}H NMR (400 MHz, $CDCl_3$): δ 5.02 (s, 1H), 4.14 (q, J = 7.2 Hz, 2H), 3.63 (s, 3H), 2.29 (s, 3H), 1.27 (t, J = 7.2 Hz, 3H); ^{13}C NMR (100 MHz, $CDCl_3$): δ 173.2, 168.1, 91.0, 59.5, 55.5, 19.0, 14.5.

Ethyl (*E*)-3-methoxy-2-butenoate (615 mg, 4.3 mmol) was dissolved in water (6.0 mL). To the solution, KOH (1.1 g, 20 mmol) was added and the mixture was stirred at room temperature overnight. The reaction was quenched with 6 M HCl up to pH 2 and extracted with diethyl ether. The combined organic layer was dried over magnesium sulfate and concentrated in vacuo to give the crude residue, which was recrystallized with diethyl ether and hexane to yield (*E*)-3-methoxy-2-butenoic acid (425 mg, 3.7 mmol, 86%). ^{1}H-NMR (400 MHz, $CDCl_3$): δ 5.04 (s, 1H), 3.67 (s, 3H), 2.30 (s, 3H); ^{13}C-NMR (125 MHz, $CDCl_3$): δ 175.3, 173.2, 90.3, 55.7, 19.3.

(*E*)-3-Methoxy-2-butenoic acid (50 mg, 0.4 mmol) was dissolved in a solution of CH_3OH/CH_2Cl_2 (3:7, 1.0 mL). To the solution, 2 M TMS diazomethane in hexane (300 µL, 0.6 mmol) was added and the mixture was stirred at room temperature for 1 h. The reaction was concentrated in vacuo to yield methyl (*E*)-3-methoxy-2-

butenoate (17.2 mg, 26%). ^{1}H-NMR (400 MHz, $CDCl_3$): δ 5.03 (s, 1H), 3.68 (s, 3H), 3.63 (s, 3H), 2.30 (s, 3H); ^{13}C-NMR (100 MHz, $CDCl_3$): δ 173.4, 168.5, 90.6, 55.5, 50.9, 19.0.

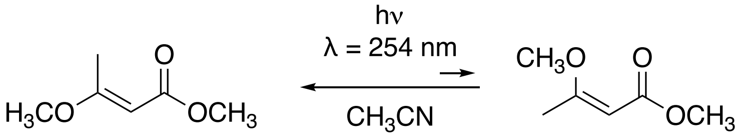

Methyl (*E*)-3-methoxy-2-butenoate (17 mg, 0.1 mmol) was dissolved in CH_3CN (5.0 mL) in a quartz test tube. The solution was stirred and irradiated at 254 nm with a xenon light source MAX-303 (Asahi Spectra Co., Ltd.) for 30 min. The resultant solution was concentrated in vacuo to give a mixture of methyl (*E*)-3-methoxy-2-butenoate and methyl (*Z*)-3-methoxy-2-butenoate, which was purified by silica-gel chromatography (hexane:ethyl acetate = 2:1) to yield methyl (*Z*)-3-methoxy-2-butenoate (approximately 1 mg). ^{1}H-NMR (500 MHz, $CDCl_3$): δ 4.91 (s, 1H), 3.84 (s, 3H), 3.65 (s, 3H), 2.03 (s, 3H); ^{13}C-NMR (125 MHz, $CDCl_3$): δ 168.3, 166.1, 95.3, 56.3, 50.8, 19.2.

Spartan 18 (Wavefunction, Inc.) was used to estimate the thermodynamic stability of the most stable conformers of (*E*)- and (*Z*)-3-methoxy-2-butenoate.

Synthesis of acetoacetyl-*N*-acetylcysteamine thioester (acetoacetyl-SNAC)[4]

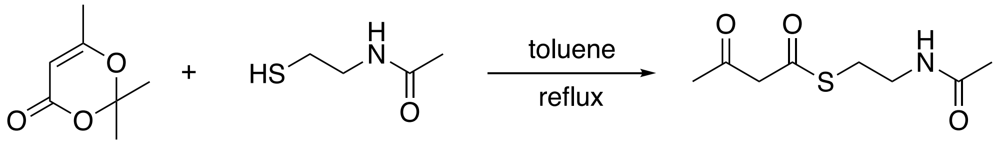

Under an argon atmosphere, a solution of *N*-acetylcysteamine (203 μL, 1.9 mmol) in toluene (4 mL) was added to a solution of 2,2,6-trimethyl-1,3-dioxin-4-one (380 μL, 2.9 mmol) in toluene (16 mL). The mixture was refluxed for 7 h and concentrated in vacuo to give the crude residue, which was purified by silica-gel chromatography and Yamazen Smart Flash EPCLC AI-580S ($CHCl_3$:CH_3OH = 15:1) to yield the *N*-acetylcysteamine thioester of acetoacetic acid (acetoacetyl-SNAC, 32 mg, 8%).

Enzymatic analysis of LynB7

Enzyme reaction with LynB7

A reaction mixture consisting of 2 mM 2-alkyl-2-thiazoline-4-carboxylic acids [or (*R*)-2-phenyl-2-thiazoline-4-carboxamide, or 2-phenyl-1,3-thiazole-4-carboxylic acid (Fujifilm Wako)], 1 mM $(NH_4)_2Fe(SO_4)_2$ (or $ZnCl_2$, $CuCl_2$, $CoCl_2$, or $MnCl_2$), 5 mM ascorbic acid, 50 µM LynB7 in the 50 mM HEPES-NaOH (pH 8.0) buffer containing 10% glycerol (total 100 µL or 20 µL) were incubated at 28 ºC for overnight.

HPLC analysis was performed with a HPLC system [Hitachi Chromastar (5110 Pump, 5310 Column Oven, 5430 Diode Array Detector, and SSC-3215 Degasser) or Hitachi ELITE LaChrom (L-2130 Pump, L-2455 Diode Array Detector, SSC-2300 Column Oven, and SSC-3215 Degasser)] with an ODS-100V (4.6 ø × 250 mm); 10 µL of solution was injected into the HPLC system.

To analyze the LynB7 reaction with 2-methyl-2-thiazoline-4-carboxylic acids, the mobile phase was a linear gradient of water and acetonitrile (20–40% (v/v) acetonitrile for 20 min) with a flow-rate of 1.0 mL/min at 40 °C. Elution was monitored at 240 nm.

To analyze the LynB7 reaction with (*R*)-2-phenyl-2-thiazoline-4-carboxylic acid, the mobile phase was a two-step linear gradient of 20 mM NaH_2PO_4/Na_2HPO_4 (pH 6.8) and methanol (45% (v/v) methanol for 5 min and 45–80% (v/v) methanol for 20 min) with a flow-rate of 1.0 mL/min at 40 °C. For the next analytical cycle, the column was washed with 80% (v/v) methanol for 5 min and equilibrated with 45% (v/v) methanol for 5 min. Elution was monitored at 300 nm.

LC-ESI-MS analysis of the LynB7 reaction with (*R*)- and (*S*)-2-methyl-2-thiazoline-4-carboxylic acids was performed with the above-mentioned LC-MS system with an ODZ-100V (2.0 ø × 150 mm); 10 µL of solution was injected into the LC-ESI-MS system. The mobile phase was 2% acetonitrile-water with 0.1% (v/v) formic acid with a flow-rate of 0.2 mL/min at 40 °C. For the next analytical cycle, the column was washed with 90% (v/v) acetonitrile with 0.1% (v/v) formic acid for 10 min. Elution was monitored at 240 nm.

For kinetic analysis of LynB7 with (*R*)- and (*S*)-2-methyl-2-thiazoline-4-carboxylic acids, and (*R*)-2-phenyl-2-thiazoline-4-carboxylic acid, a reaction mixture consisting of a variable concentration of 2-methyl-2-thiazoline-4-carboxylic acids or (*R*)-2-phenyl-2-thiazoline-4-carboxylic acid (1.0, 2.0, 4.0, 8.0, and 16.0 mM) 1 mM Fe^{2+}, 5 mM ascorbic acid, 50 µM LynB7 in the 50 mM HEPES-NaOH (pH 8.0) buffer containing 10% glycerol (total 50 µL) were incubated at 28 ºC for 10, 20, and 30 min. The reactions were quenched with 50 µL of CH_3CN and the resultant solution was analyzed by the above-mentioned HPLC. The amounts of the products were estimated from standard curves, which were made with the authentic 2-methylthiazole (TCI) or 2-phenylthiazole (Fujifilm Wako). Data were fitted to the Michaelis-Menten equation using KaleidaGraph 4.5 (HULINKS Inc., Japan) to estimate kinetic constants (**Figure S13**).

To estimate the primary deuterium kinetic isotope effect (KIE) upon the reaction of LynB7 with (4*R*)-[5,5-$^2H_2$]-2-phenyl-2-thiazoline-4-carboxylic acid against non-labelled (4*R*)-2-phenyl-2-thiazoline-4-carboxylic acid, a reaction mixture consisting of 2 mM each substrate, 1 mM $(NH_4)_2Fe(SO_4)_2$, 5 mM ascorbic acid, 50 µM LynB7 in the 50 mM HEPES-NaOH (pH 8.0) buffer (total 20 µL) were incubated at 28 ºC for 5, 10, 15,

30, and 45 min. The reaction was quenched with 20 μL of methanol and analyzed by the above-mentioned HPLC. An average of three replicates at each time point was plotted to estimate the reaction rates, which were compared to obtain the KIE value (**Figure S19**).

Preparation substrate analogs

Synthesis of (4*R*)- and (4*S*)-2-methyl-2-thiazoline-4-carboxylic acids (Figure S11)

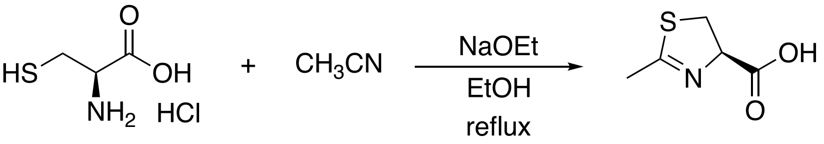

Under an argon atmosphere, 20% sodium ethoxide solution in ethanol (800 μL, 2 mmol) was added to a solution of L-cysteine HCl salt (175 mg, 1 mmol) in super dehydrated ethanol (10 mL). To the mixture, CH_3CN (60 μL, 1.2 mmol) was added and the solution was refluxed for 8 h. After solid material was filtered, the filtrate was concentrated in vacuo to give (4*R*)-2-methyl-2-thiazoline-4-carboxylic acid (57 mg, 0.39 mmol, 39%). ^{1}H-NMR (400 MHz, D_2O): δ 4.90 (m, 1H), 3.65 (dd, *J* = 11.2 and 9.8 Hz, 1H), 3.42 (dd, *J* = 11.2 and 7.9 Hz, 1H), 2.24 (s, 3H); ^{13}C-NMR (400 MHz, D_2O): δ 179.0, 172.8, 79.4, 37.3, 19.2; HRMS (FAB, glycerol matrix, positive mode): $[M–H+Na_2]^+$ ion at *m*/z 189.9909 (calcd $[M–H+Na_2]^+$ ion for $C_5H_6NO_2SNa_2$ at *m*/*z* 189.9909).

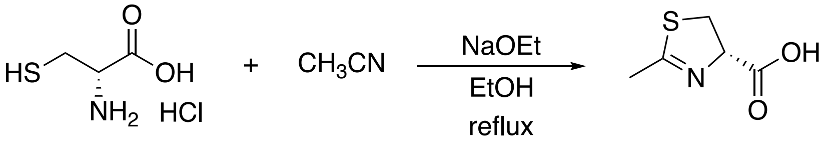

(4*S*)-2-methyl-2-thiazoline-4-carboxylic acid was synthesized by the same method for the synthesis of (4*R*)-isomer from D-cysteine HCl salt (175 mg, 1 mmol). The yield was 51 mg, 35%. ^{1}H-NMR (400 MHz, D_2O): δ 4.90 (m, 1H), 3.65 (dd, *J* = 11.2 and 9.8 Hz, 1H), 3.41 (dd, *J* = 11.2 and 7.9 Hz, 1H), 2.24 (d, *J* = 1.6 Hz, 3H); ^{13}C-NMR (400 MHz, D_2O): δ 179.1, 172.9, 79.5, 37.3, 19.2; HRMS (FAB, glycerol matrix, positive mode): $[M–H+Na_2]^+$ ion at *m*/z 189.9920 (calcd $[M–H+Na_2]^+$ ion for $C_5H_6NO_2SNa_2$ at *m*/*z* 189.9909).

Synthesis of (4*R*)-2-phenyl-2-thiazoline-4-carboxylic acid

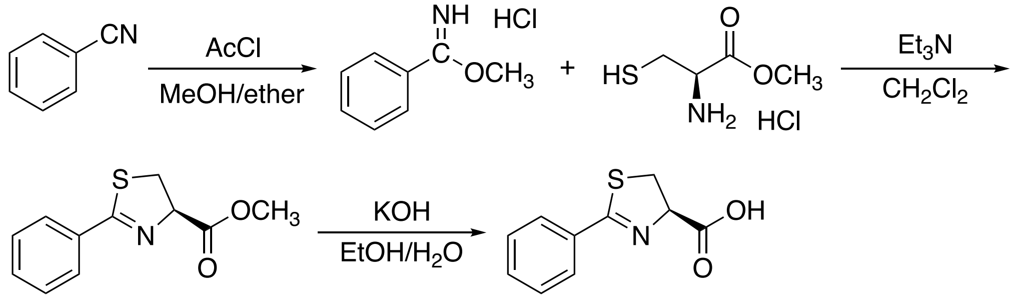

Under an argon atmosphere in an ice-water bath, acetyl chloride (4.3 mL, 60 mmol) was added to a solution of CH_3OH (5 mL) and diethyl ether (30 mL), and stirred for 15 min. To the mixture, benzonitrile (5.1 mL, 50 mmol) was added and stirred at room temperature for 3 days. The resultant precipitate was recovered by filtration, washed with cold diethyl ether, and dried in vacuo to give methyl benzimidate hydrochloride (2.0 g).

Under an argon atmosphere, L-cysteine methyl ester hydrochloride (3.3 g, 19 mmol) was added to a solution of methyl benzimidate hydrochloride (2.0 g, 19 mmol) in CH_2Cl_2 (25 mL). After the reaction apparatus was placed in an ice-water bath, triethylamine (2.7 mL, 19 mmol) was added and the mixture was stirred at room temperature for 24 h. The reaction was quenched with water. The mixture was extracted with CH_2Cl_2 and the extract was washed with brine. The combined organic layer was dried over sodium sulfate and concentrated in vacuo to yield a crude residue, which was purified by silica-gel chromatography (hexane:ethyl acetate = 5:1) to yield methyl (4*R*)-2-phenyl-2-thiazoline-4-carboxylate (1.36 g, 6.2 mmol, 32%). ^{1}H-NMR (600 MHz, $CDCl_3$): δ 7.86 (d, *J* = 7.0 Hz, 2H), 7.48 (t, *J* = 7.4 Hz, 1H), 7.41 (t, *J* = 7.8 Hz, 2H), 5.29 (t, *J* = 9.1 Hz, 1H), 3.84 (s, 3H), 3.71 (dd, *J* = 11.1 and 8.9 Hz, 1H), 3.64 (dd, *J* = 11.1 and 9.4 Hz, 1H); ^{13}C-NMR (150 MHz, $CDCl_3$): δ 171.4, 171.0, 132.7, 131.7, 128.6, 128.5, 78.5, 52.8, 35.4; HRMS (FAB, NBA matrix, positive mode): $[M+H]^+$ ion at *m*/z 222.0595 (calcd $[M+H]^+$ ion for $C_{11}H_{12}NO_2S^+$ at *m*/*z* 222.0583).

Methyl (4*R*)-2-phenyl-2-thiazoline-4-carboxylate (452 mg, 2.04 mmol) was dissolved in a solution of ethanol/water (2:1, 18 mL). To the solution, 2 M KOH (2.0 mL) was added and the mixture was stirred at room temperature for 2 h. The solution was acidified to pH 2 with 1 M HCl and the mixture was extracted with ethyl acetate. The combined organic layer was dried over sodium sulfate and concentrated in vacuo to yield (4*R*)-2-phenyl-2-thiazoline-4-carboxylic acid (393 mg, 1.89 mmol, 92%). ^{1}H-NMR (600 MHz, $CDCl_3$): δ 7.77 (d, *J* = 7.2 Hz, 2H), 7.42 (t, *J* = 7.4 Hz, 1H), 7.33 (t, *J* = 7.8 Hz, 2H), 5.22 (t, *J* = 9.6 Hz, 1H), 3.57 (m, 2H); ^{13}C-NMR (150 MHz, $CDCl_3$): δ 174.5, 172.5, 132.2, 131.9, 128.58, 128.56, 78.4, 35.2; HRMS (FAB, NBA matrix, positive mode): $[M+H]^+$ ion at *m*/z 208.0439 (calcd $[M+H]^+$ ion for $C_{10}H_{10}NO_2S$ at *m*/*z* 208.0468).

Synthesis of (4*R*)-[5,5-$^{2}H_2$]-2-phenyl-2-thiazoline-4-carboxylic acid

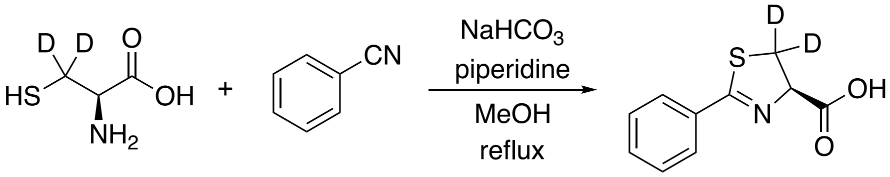

Under an argon atmosphere, L-[3,3-$^{2}H_2$]cysteine (20 mg, 0.13 mmol; 98% purity, Cambridge Isotope Laboratories, Inc.), sodium bicarbonate (11 mg, 0.13 mmol), and benzonitrile (13 μL, 0.13 mmol) were mixed in methanol (2 mL). The solution was heated at 80 ºC for 30 min. After cooling, piperidine (13 μL, 0.13 mmol) was added to the solution and was stirred at 80 ºC for overnight. The solution was concentrated by evaporator and the residue was dissolved in water, adjusted pH 10 with 2 M KOH. The mixture was extracted with diethyl ether. The resultant water layer was acidified to pH 2 with 1 M HCl and extracted with $CHCl_3$. The combined organic layer was dried over sodium sulfate and concentrated in vacuo to yield (4*R*)-[5,5-$^{2}H_2$]-2-phenyl-2-thiazoline-4-carboxylic acid (12 mg, 0.071 mmol, 44%). ^{1}H-NMR (600 MHz, $CDCl_3$): δ 7.83 (d, J = 7.6 Hz, 2H), 7.49 (t, J = 7.5 Hz, 1H), 7.41 (t, J = 7.6 Hz, 2H), 5.31 (s, 1H); ^{13}C-NMR (150 MHz, $CDCl_3$): δ 173.4, 173.0, 132.2, 132.0, 128.7, 128.6, 77.9, 34.5 (m); HRMS (FAB, NBA matrix, positive mode): $[M+H]^+$ ion at *m*/z 210.0560 (calcd $[M+H]^+$ ion for $C_{10}H_8D_2NO_2S$ at *m*/*z* 210.0552).

Synthesis of (4*R*)-2-phenyl-2-thiazoline-4-carboxamide

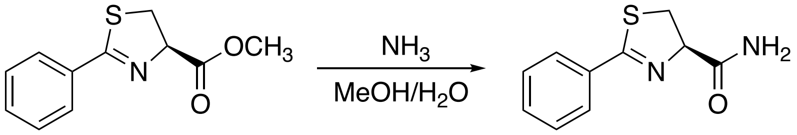

Methyl (4*R*)-2-phenyl-2-thiazoline-4-carboxylate (109 mg, 0.49 mmol) was dissolved in a solution of methanol (2 mL) and 30% ammonia solution (0.3 mL) and the mixture was stirred at room temperature overnight. The solution was diluted with water and extracted with $CHCl_3$. The combined organic layer was washed with brine, dried over sodium sulfate, and concentrated in vacuo to yield a crude residue, which was purified by silica-gel chromatography ($CHCl_3$:CH_3OH = 50:1) to yield (4*R*)-2-phenyl-2-thiazoline-4-carboxamide (53 mg, 0.26 mmol, 53%). ^{1}H-NMR (600 MHz, $CDCl_3$): δ 7.87 (d, J = 7.3 Hz, 2H), 7.51 (t, J = 7.4 Hz, 1H), 7.44 (t, J = 7.8 Hz, 2H), 6.79 (s, 1H), 5.49 (s, 1H), 5.21 (t, J = 9.5 Hz, 1H), 3.73 (m, 2H); ^{13}C-NMR (150 MHz, $CDCl_3$): δ 173.7, 171.1, 132.6, 131.9, 128.6, 128.4, 79.1, 35.3; HRMS (FAB, NBA matrix, positive mode): $[M+H]^+$ ion at *m*/z 207.0594 (calcd $[M+H]^+$ ion for $C_{10}H_{11}N_2OS^+$ at *m*/*z* 207.0587).

Table S1. Summary of draft genome sequence analysis of *Moorena bouilloni* strain 1509-15.

**Number of total contig**	547
**Number of total base pairs of contig**	16,522,888
**Number of GC base pairs**	7,194,642
**GC contents (%)**	43.5
**Average length of contig**	30,206
**Maximum length of contig**	545,168
**N50 length of contig**	57,386

Table S2. Lyngbyapeptin B (*lynB*) biosynthetic genes and their putative functions.

Gene Designation	Size (AA)	Encoded Protein Homologues (AA, GenBank Accession Numbers, source strain) % Identity/%Positives	Putative function
lynB1	1535	CurA GNAT family *N*-acetyltransferase (1448 (in 2311), AEE88289,[5] *Moorea producens* 3L, AAT70096,[6] *Lyngbya majuscula*), 77/87	PKS (AR-GNAT-ACP-KS-AT(Mal))
lynB2	485	JamN, methyltransferase (1-454 (in 488), AAS98785,[7] *Lyngbya majuscula*), 69/81<br>BarF, methyltransferase (8-469 (in 504), AAN32980,[8] *Lyngbya majuscula*), 64/77<br>MtaF, methyltransferase (892-1340 (in 1360), ADO71804,[9] *Stigmatella aurantiaca* DW4/3-1), 39/62	PKS (OMT-ACP)
lynB3	1894	VatN, NRPS (C-A-MT-MT-PCP-TE), (1863 (in 2153), QCP68969,[10] *Moorea producens* ASI16Jul14-2), 87/92	NRPS (C-A(Tyr)-OMT-NMT-PCP)
lynB4	1551	NRPS, (1-1529 (in 1958), AOW98528,[11] *Moorea producens* PAL-8-15-08-1), 90/94	NRPS (C-A(Val)-NMT-PCP)
lynB5	1868	VatN, NRPS (C-A-MT-MT-PCP-TE), (60-1867 (in 2153), QCP68969,[10] *Moorea producens* ASI16Jul14-2), 78/85<br>NRPS, (43-1878 (in 2303), EGJ29402,[5] *Moorea producens* 3L), 88/93	NRPS (C-A(Tyr)-OMT-NMT-PCP)
lynB6	2474	BarG, NRPS (1359-2887 (in 2887), AAN32981,[8] *Lyngbya majuscula*), 70/83; (1-993 (in 2887), AAN32981,[8] *Lyngbya majuscula*), 44/61	NRPS (C-A(Ala)-PCP- Cy-A(Cys)-PCP-TE)
lynB7	363	BarH, putative amidohydrolase (360, AAN32982,[8] *Lyngbya majuscula*), 77/88	Nonheme diiron oxygenase
lynB8	297	NRPS, (1068-1338 (in 1360), AOX04306,[11] *Moorea producens* PAL-8-15-08-1), 82/91	Type II thioesterase
lynB9	137	DUF433 domain-containing protein, (124, EGJ31130,[5] *Moorea producens* 3L), 95/96	Hypothetical protein

Table S3. Non-ribosomal codes of adenylation enzymes (LynB3-A, LynB4-A, LynB5-A, LynB6-A1, and LynB6-A2) in this manuscript.

Adenylation enzyme	Substrate Amino acid	A1	A2	A3	A4	A5	A6	A7	A8	A9	A10
PheA	L-Phe	D	A	W	T	I	A	A	I	C	K
LynB3-A	L-Tyr	D	A	S	T	V	A	A	V	C	–*
LynB4-A	L-Val	D	A	L	W	L	G	G	T	F	–*
LynB5-A	L-Tyr	D	A	S	T	V	A	A	V	C	–*
LynB6-A1	L-Ala	D	L	F	N	V	A	L	T	Y	K
LynB6-A2	L-Cys	D	L	Y	N	L	S	L	I	W	K

*The catalytically important Lys residues at the C-terminal of adenylation enzymes in LynB3, LynB4, and LynB5 are located in the front of the PCP domains, because the MT domains are stuffed between the N-terminus adenylation (A_{core}) domain and the C-terminus adenylation (A_{sub}) domain of NRPSs.[12, 13]

Table S4. Artificial genes for *lynB2* and *lynB7* to express in *E. coli*.

Artificial *lynB2* gene for expression in *E. coli*. The red sequence was designed for the expression of LynB2-OMT and the magenta sequence was designed for the expression of LynB2-ACP. Restriction enzyme sites are underlined.

5’-
CATATGTCTGACATTGAAAATCGTCTTGCAAAACTCTCTCCGGAACAGCGCAAAGTACTGGAGA
AAAAAATCCTACAGAGCGACAAATACACCGTTGATGTTCCGCAGAATAGCAACGAAGAGAAAC
TGAATTCTTACTATAAGTCGTTAGGTGTTAAGAATGAAACCGCCAAAGACTATGTACGCTTTGC
TCCTTTTCCGCAGACCATTTCAGGGTTTTCCTGGATTGAGATCATGCTTGAGCCTGGTAACTCAC
CCGAACAGAATGAACTGTCTCTGAAATACCAAGAGGAGATGAAACGGGTTCTGTTTCGAGGGA
TCGAATTTTCGTCGATTGATAAGGTCATGGATATTGGTTGCGGCTATAGCTATGACCTGATCGA
TCTGGCGGAAAAACACCCGCATCTGCAGCTGAATGGTTACAACATTAGCCCAGAACAGGTGAA
AATCGGCAAAGAAAAAGTGGAGTCTTTAGGCTATTCCCAGCGTATCAACATCTACAACCGTGA
CAGTGCGAAACAACCATTTCCGGATGAATACAATCTGATTTATAGCTGTCAGGTGATCCACCAC
ATGAAAAAGAAGGAAGATGTGTTCCTGAACATGTCCAAGCATCTCTCAAATGGCGGCTTTTTCG
TAGCTGCCGAAATCATCTCCAACCTGCCGTTAACTCCGATTGAAGATCCGAAAAGTACCGCGTA
TTTTGCCACTCGTAGTAAATGGGCTGAACTTCTGGCCCAAAACAACTTACGGGTCGTTGAAGCG
GTGGATGCATCATTCGAGATTGGCAATTATCTGAACGATGTTAACTTCACGGAAAATTTCACGC
GTTTGACCCAGAACTATGACAAAATTGCGAAAGAGCATCTGAAAGGCCCTCATGAACTGGGTG
AACTGCTCCGCAAAAAGTTGGCAGTGTATATGCTGATAACCGTGCAGAAAGATAACTTCATCG
AAAAAGACACAATTCTGCGCTTGAACCAGGAAAAACTAAGCGATTTGGTACCGTATGCCAAAA
TCATTGAAGCATCCTCTGATGGTAAAATGCTGCTGTTACCGCAACTGAGTCGCGAAGAAAGCAA
TGTCGTCAAAGAAAAGGCGAAAAACTTCCGTGAACAGTTTACCTCACAAGAACCAAGCCAGAT
TATCGATTGGCTGAAGTACTACTTCAAAACACAAGTTGCGAATATCCTGCAAACTTCGCAAGCT
CAGGTGGAATTGGAAAAGAGCCTCAATTTGATGGGCTTTGACTCGCTTATGTTCATGGAACTGA
GAAATCGCATTCAGAACGACTTAATCATTGATATAGGAGTCGCGGATTTAATGGGAGGGATCA
CGATAAGTAAACTGGCCAGCAAAGTGAGCGAACTTGCATCCAAAATTAACAGCAACTTGGAGC
CCAAAGAGGAGTTTATTAGTATTCATTCGCAAGAGACGACGACCATTGAGGGTACACTCAAGC
TT-3’

Artificial *lynB7* gene for expression in *E. coli*. The green sequence was designed for the expression of LynB7. Restriction enzyme sites are underlined.

5’-
CATATGGTCAAAACGTATGAAATCATTGACGCCGATAGCCATGTACTGGAACCTCTTGATCTGT
GGGAGAAGTATCTCGAACCGGAGTTTAAACACTTTGCCCCAAAAGAGGACTACAAATTAGAGG
GAGAGCCGATCCTGTATAAATTCAACCCATCCGATTTTGTGGAATCGGAAGCTGCCAAACGTGC
ACAGGAGGATCCGGATATCTACAAAGGCGGTTATGACCCAAAAGCGCGTGTCGAATTGATGGA
ACAAATGGGTAGTGACATGACCTTTCTGTACCCTACCATTGGACTGTGGATGTGGAGCGTTGAC
ACCATGGATAGCAAACTTGCTGGCGCATTTGTTCGTTCCTACAATAACTGGCTGCATGACTTCT
GCAACTATGCCCCACAGAAACTGAAGGGTATTGGCGCGATTAATCAGCATGATCCTGAAGAAA
TGGTTCCTGAACTGCAGCGGATTGTGGAATTCGGCTGGAAAGCCGTATTCATTCGCCCGAATCC
CGTGAAAGGGCGCATTCTCTCAGATCCAGTTTACGAACCGTTTTGGTCGGAATGTGAACGCCTC
AATGTCGCGGTGACAGTGCACGAAGGCACCCATTCACGCTTACCGACGACTGGTGCAGATCGG
TTCGATTCGCGCTTTGCACTGCATGCGTGCAGTCATCCGATGGAACAGATGATGGCGTTGTTAG
CTCTGATCGAAGGTGGGGTACTTGAACGTCATCCGCAACTTCGCGTTGGGTTCTTCGAATCTGG
CTGTGGCTGGCTGCCCTATTGGCTCTGGCGTTTAGATGAGGAGTATGAGAACTTGTACTATGAA
GTCAAAGACAACGTGAAGATGAAACCGTCTGAGTACTTTCACCGTCAATGCTACACTGGCTTTG
AAGTGGATGAACCCTATCTGGCCGAACTGATTGAGTACATTGGTGCGGATAAACTGCTGTTCGG
TAGCGACTTTCCGCACATGGATCACCCGAACTTTATCGAGAGCGTCGATGAAACGCTGGCGTTA
CGCGAAAAGCTGCCGGAAGATGTGATCAAGAAAATCCTGCACGACAATCCGTTGCGCTATTAT
GGCCTGACCTAACTCGAG-3’

Figure S1. Expression and purification of (A) LynB2-OMT, (B) LynB2-ACP, and (C) LynB7.

Lane 1 marker; lane 2 precipitate; lane 3 supernatant; lane 4 flow through; lane 5 washed with 20 mM imidazole; lane 6 eluted with 200 mM imidazole; lane 7 concentrated.

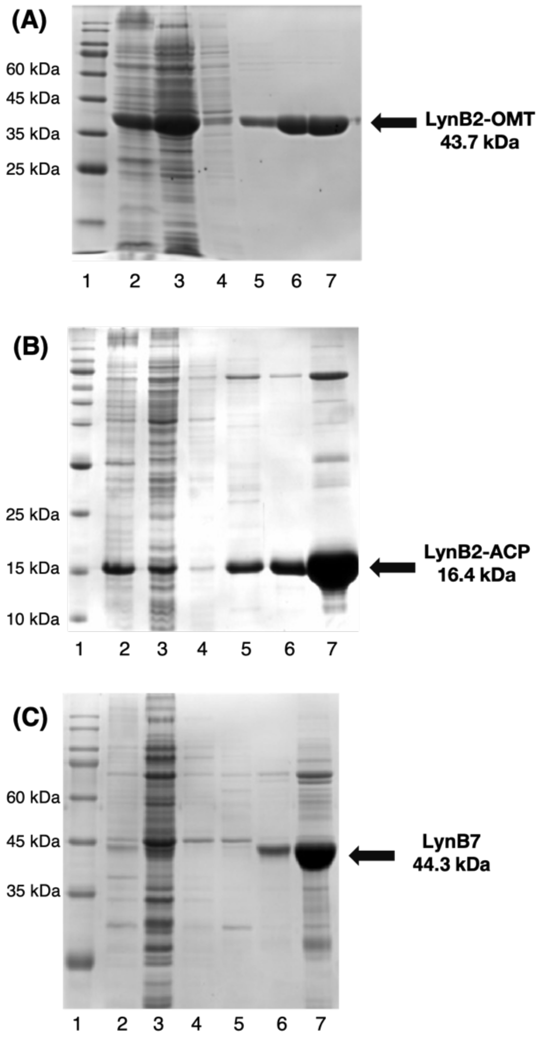

Figure S2. ESI-MS analysis of the LynB2-OMT reaction products. Mass spectra (left) and deconvoluted mass spectra (right). (1) Acetoacetyl-LynB2-ACP (estimated mass 16806 Da), (2) methylated product from acetoacetyl-LynB2-ACP (estimated mass 16820 Da), and (3) apo-LynB2-ACP (estimated mass 16382 Da).

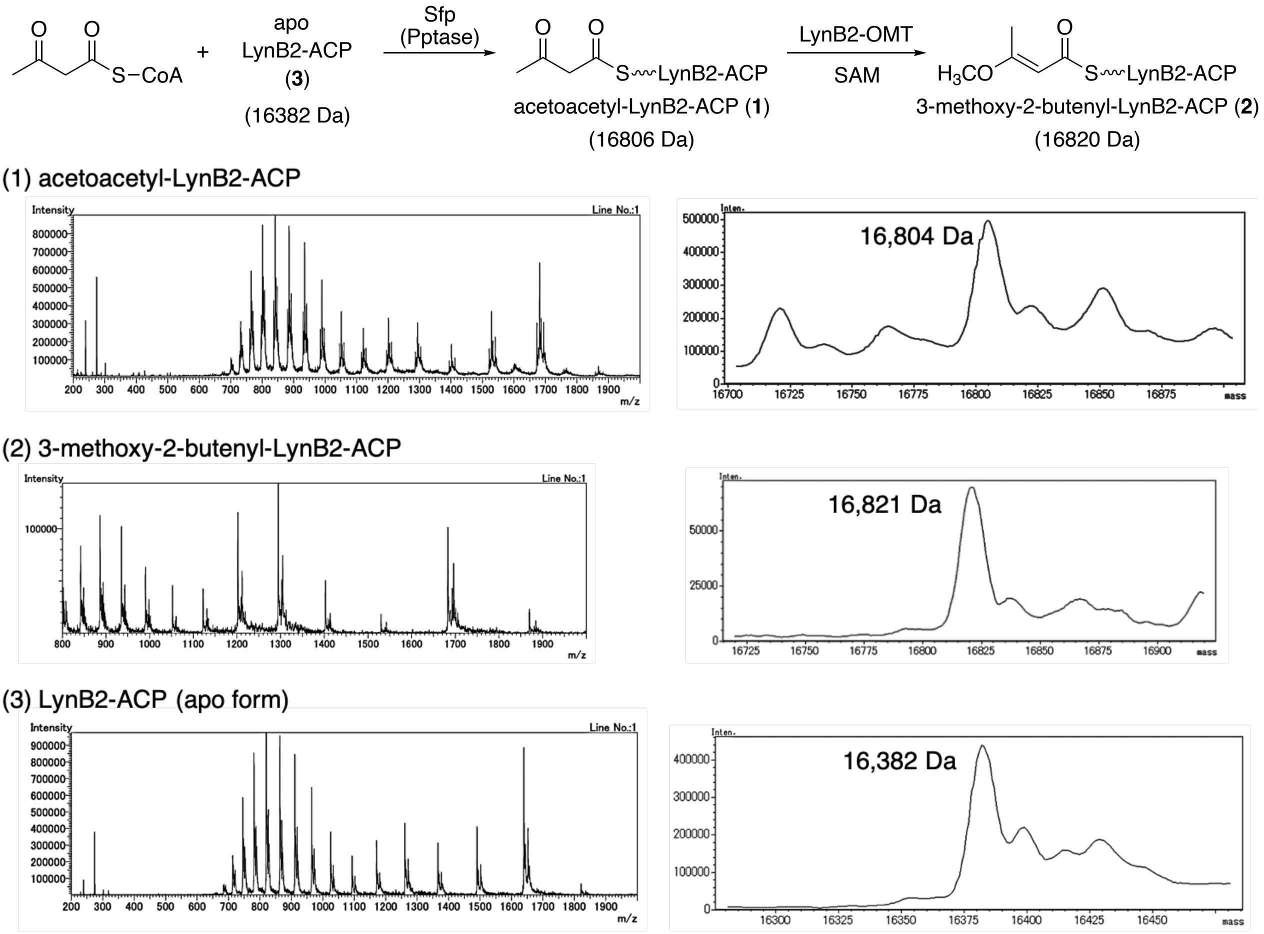

Figure S3. HPLC analysis of the LynB2-OMT reaction with acetoacetyl-SNAC (A) and acetoacetyl-CoA (B).

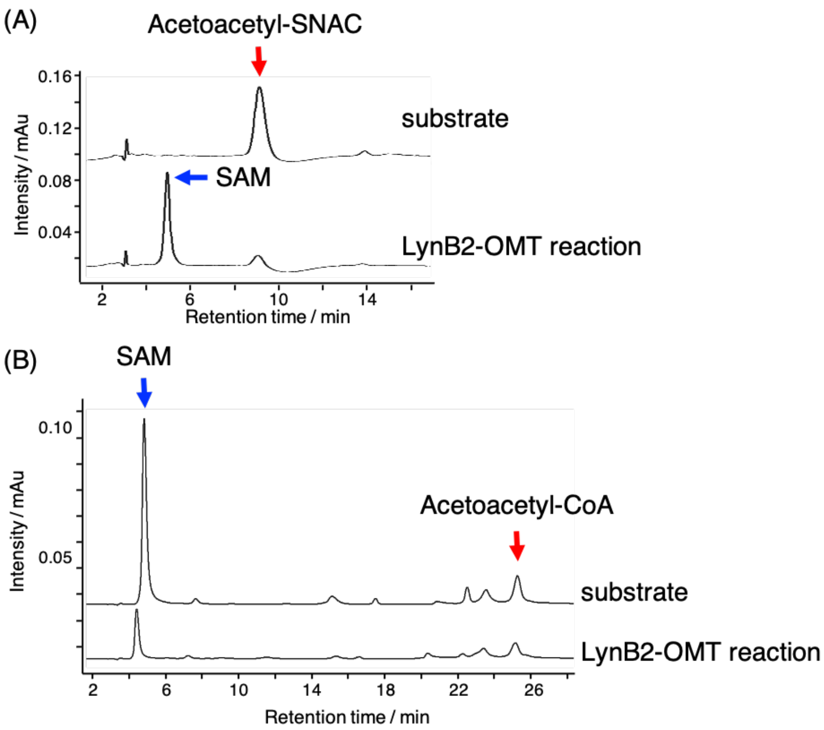

Figure S4. NMR spectra of methyl (*E*)-3-methoxy-2-butenoate for this study.

^{1}H-NMR (400 MHz, $CDCl_3$)

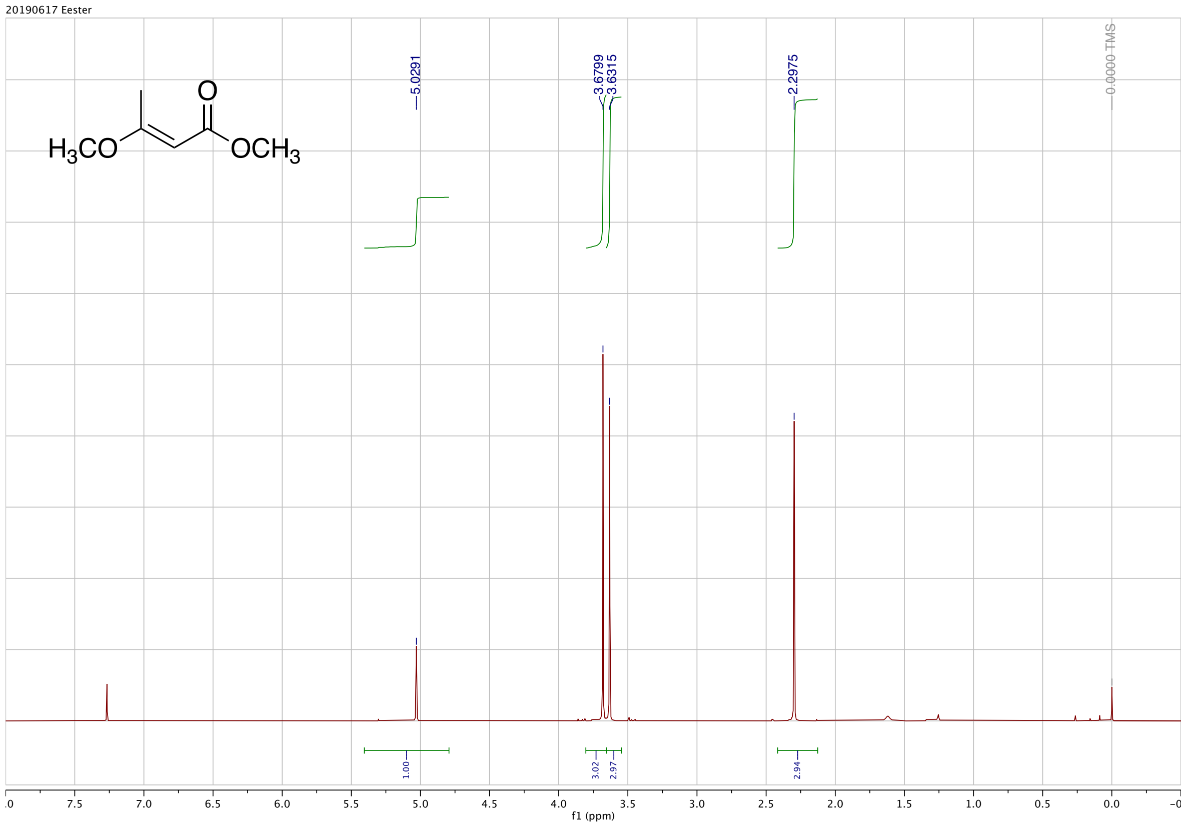

NOESY (500 MHz, $CDCl_3$)

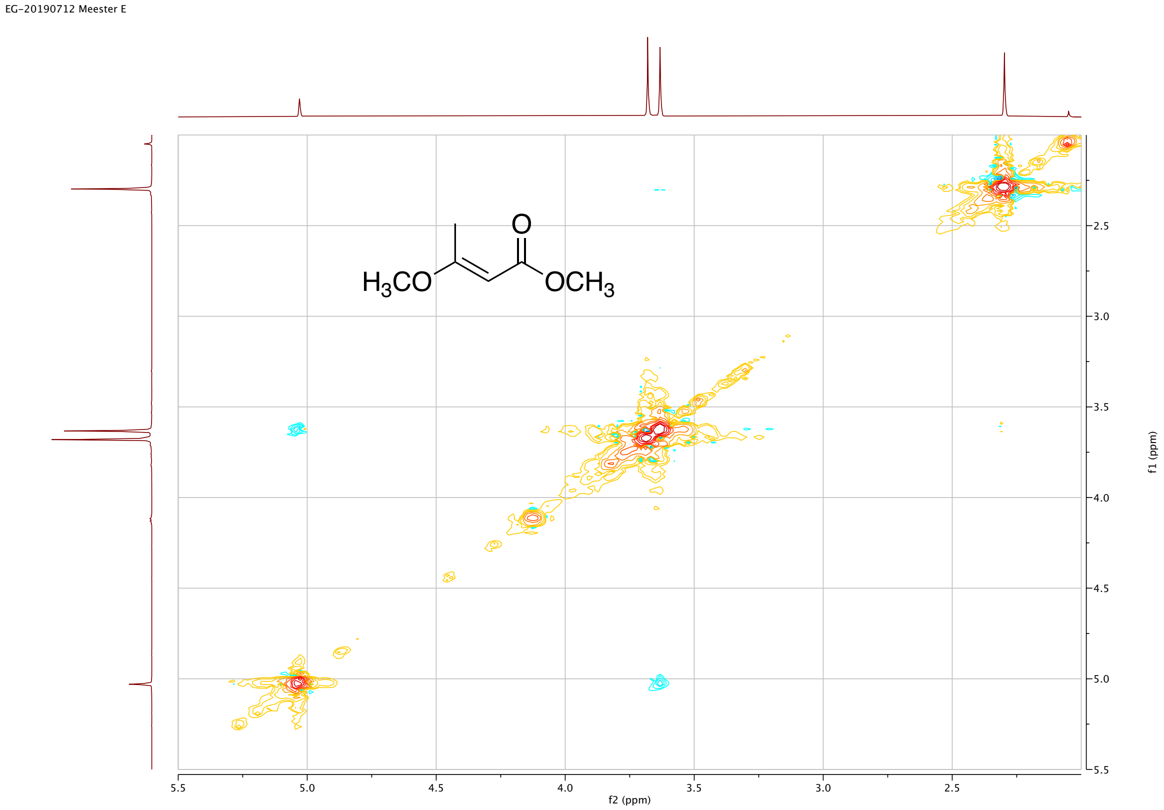

^{13}C-NMR (100 MHz, $CDCl_3$)

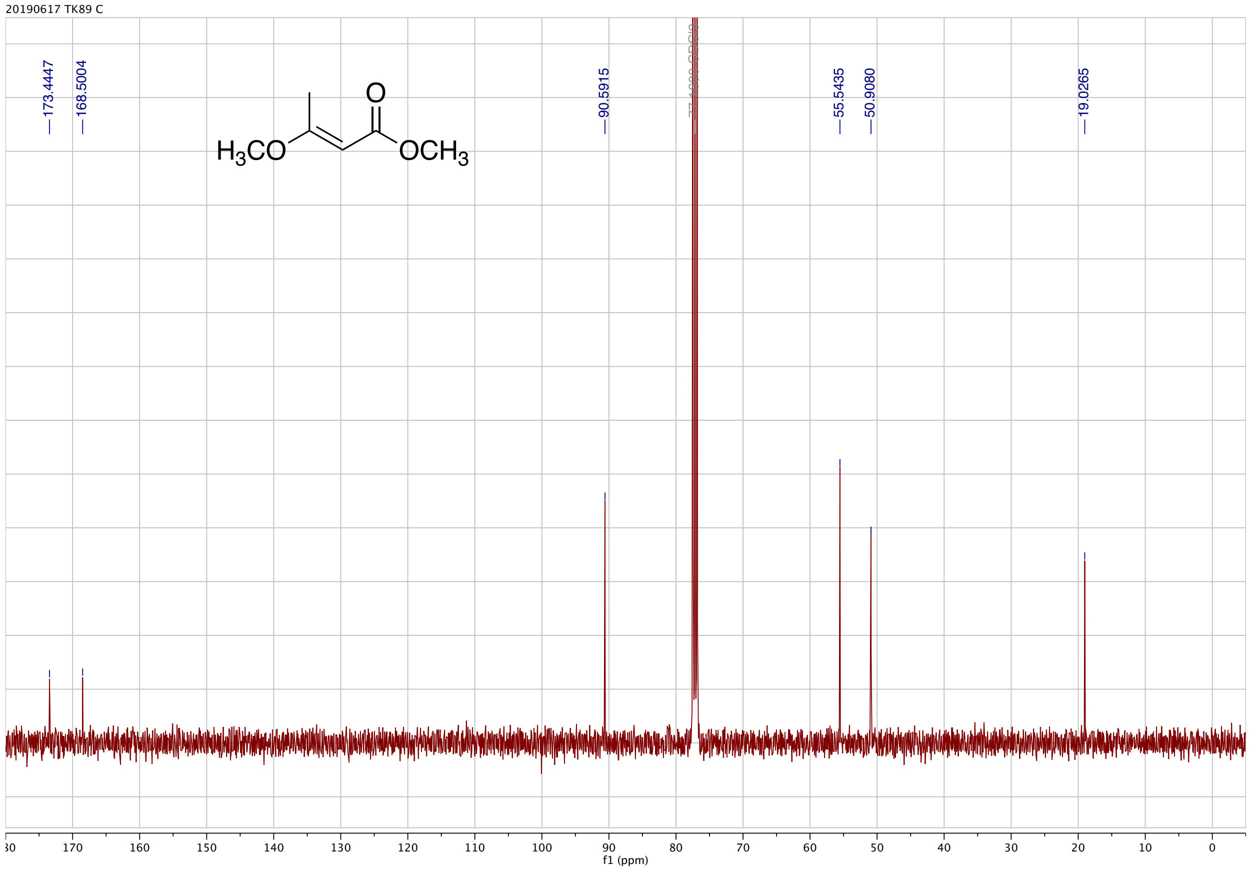

Figure S5. NMR spectra of methyl (*Z*)-3-methoxy-2-butenoate for this study.

^{1}H-NMR (500 MHz, $CDCl_3$)

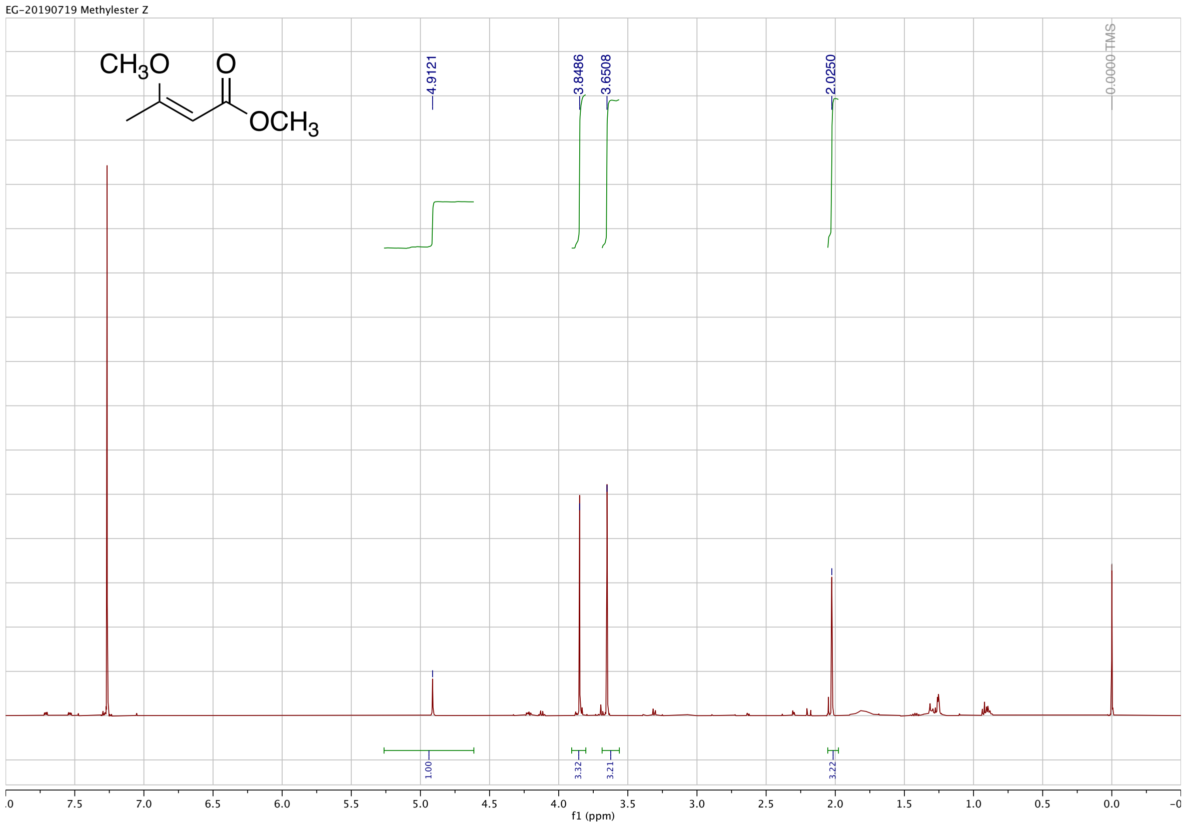

NOESY (500 MHz, $CDCl_3$)

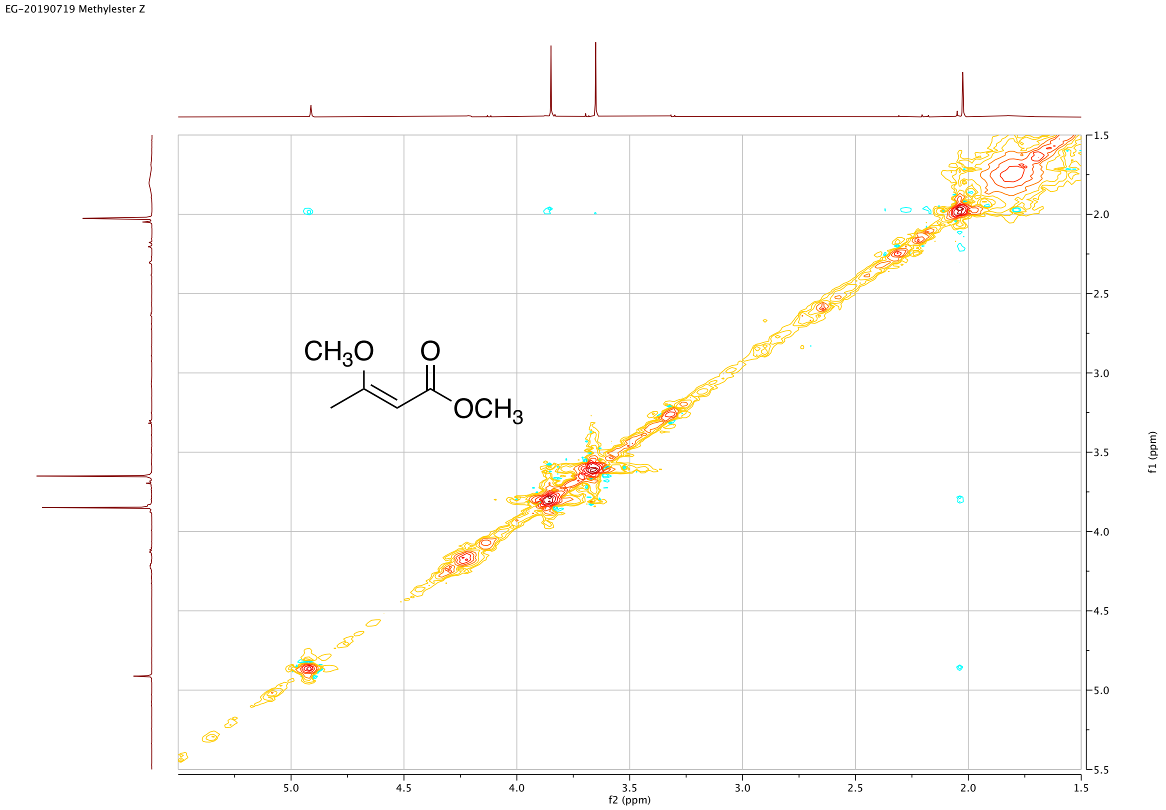

^{13}C-NMR (125 MHz, $CDCl_3$)

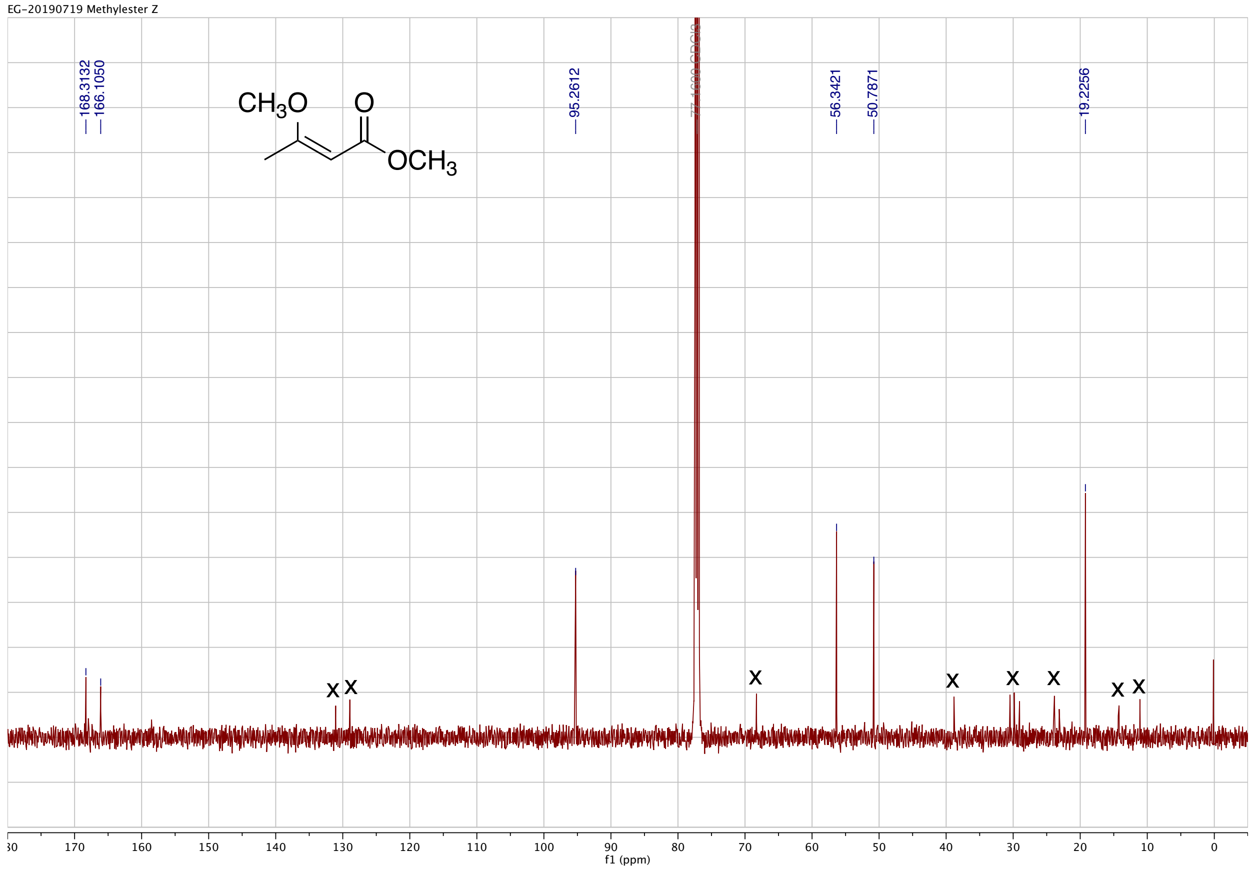

Figure S6. Comparison of ^{1}H-NMR spectra for methyl (*E*)-3-methoxy-2-butenoate, methyl (*Z*)-3-methoxy-2-butenoate, and methyl (*Z*)-3-methoxy-2-butenoate after 3 days in the NMR solvent ($CDCl_3$) at room temperature. Only a small amount of (*E*)-isomer was observed in the solution of (*Z*)-isomer after 3 days. X is residual ethyl acetate in the experiment.

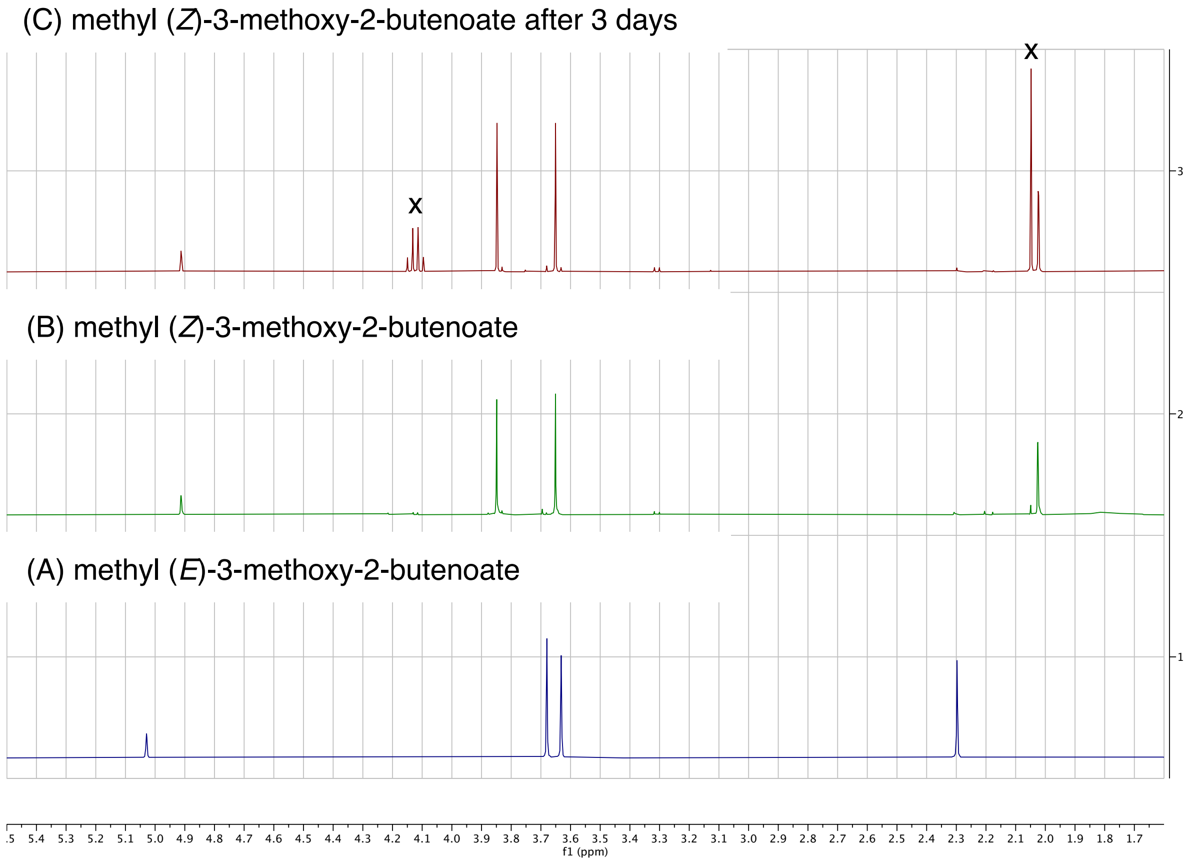

Figure S7. HPLC and LC-ESI-MS analysis of off-loaded products from the LynB2-OMT reaction products. (A) HPLC analysis of offloaded product formed by LynB2-OMT, and (B) Mass spectra of methyl (*Z*)-3-methoxy-2-butanoate (left), methyl (*E*)-3-methoxy-2-butanoate (middle) and offloaded product (right).

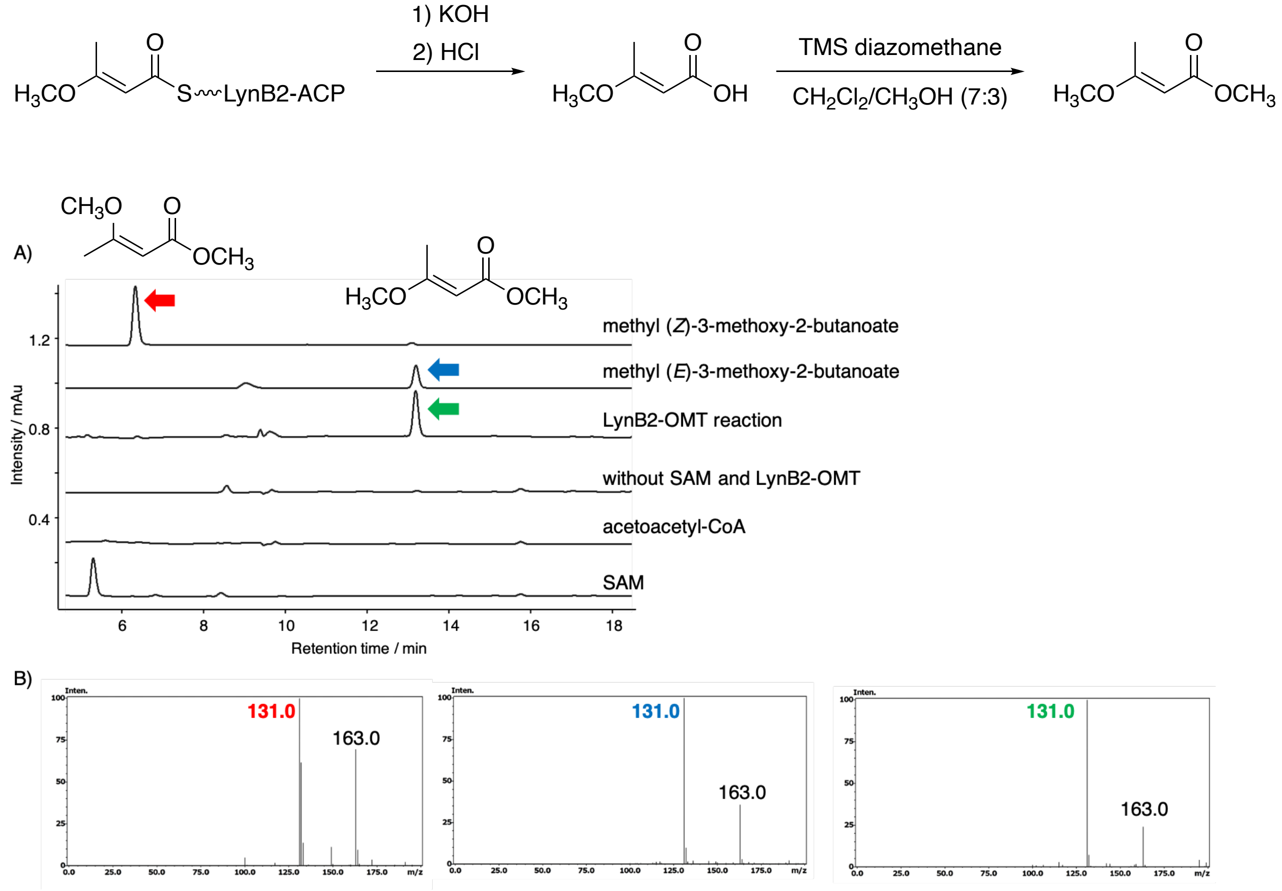

Figure S8. Predicted model structure (A) and active site (B) of LynB2-OMT (green) are superimposed on the structures of StiE_OMT (PDB entry 6ecx, RMSD = 1.0 Å, cyan) and StiD_OMT (PDB entry 6ecu, RMSD = 1.2 Å, purple). (C) Structures of stigmatellin A, myxothiazol and cystothiazole A, whose biosynthetic pathways use related *O*-methyltransferases and are discussed in the main text.

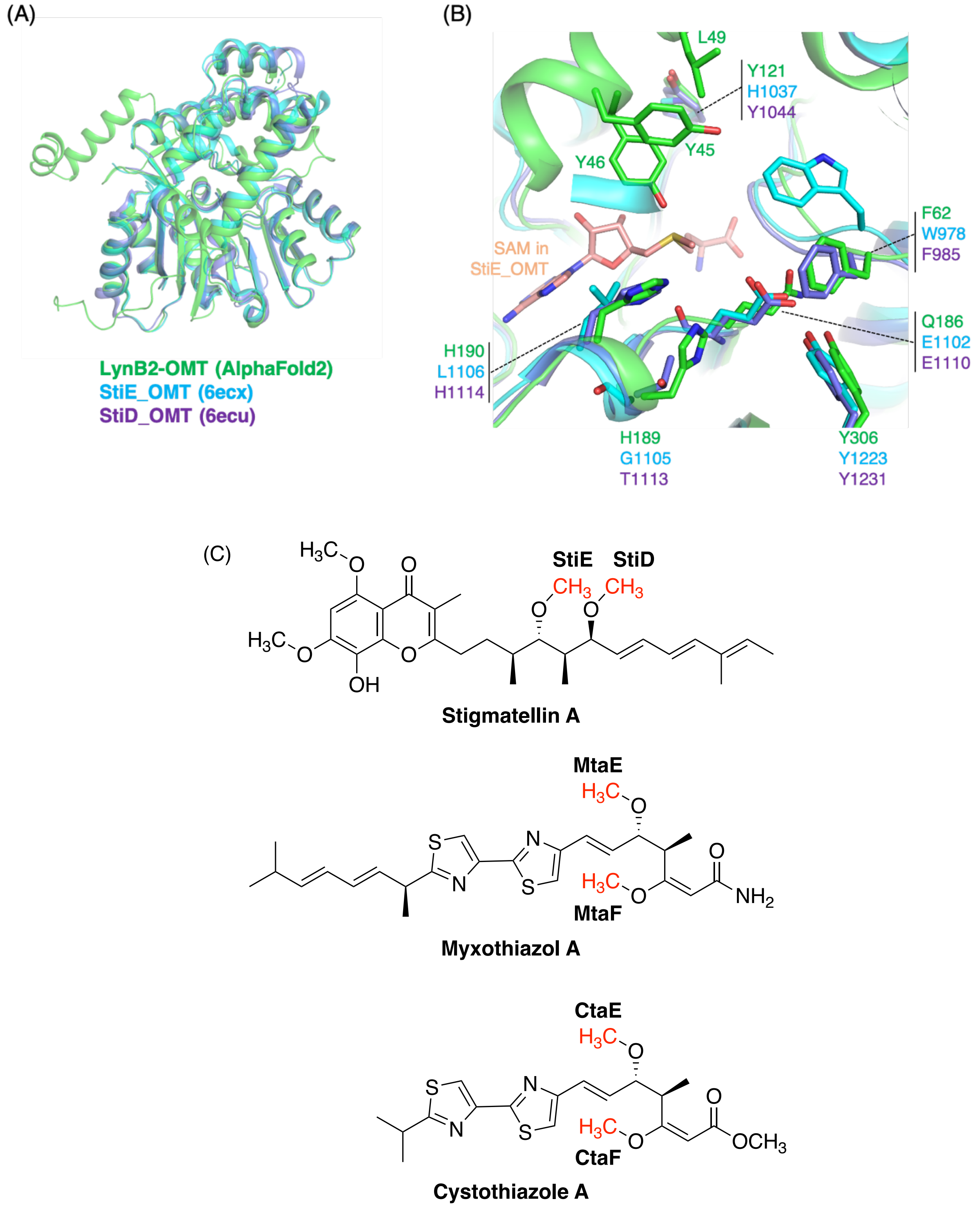

Figure S9. Sequence alignment of the LynB2-OMT family proteins.

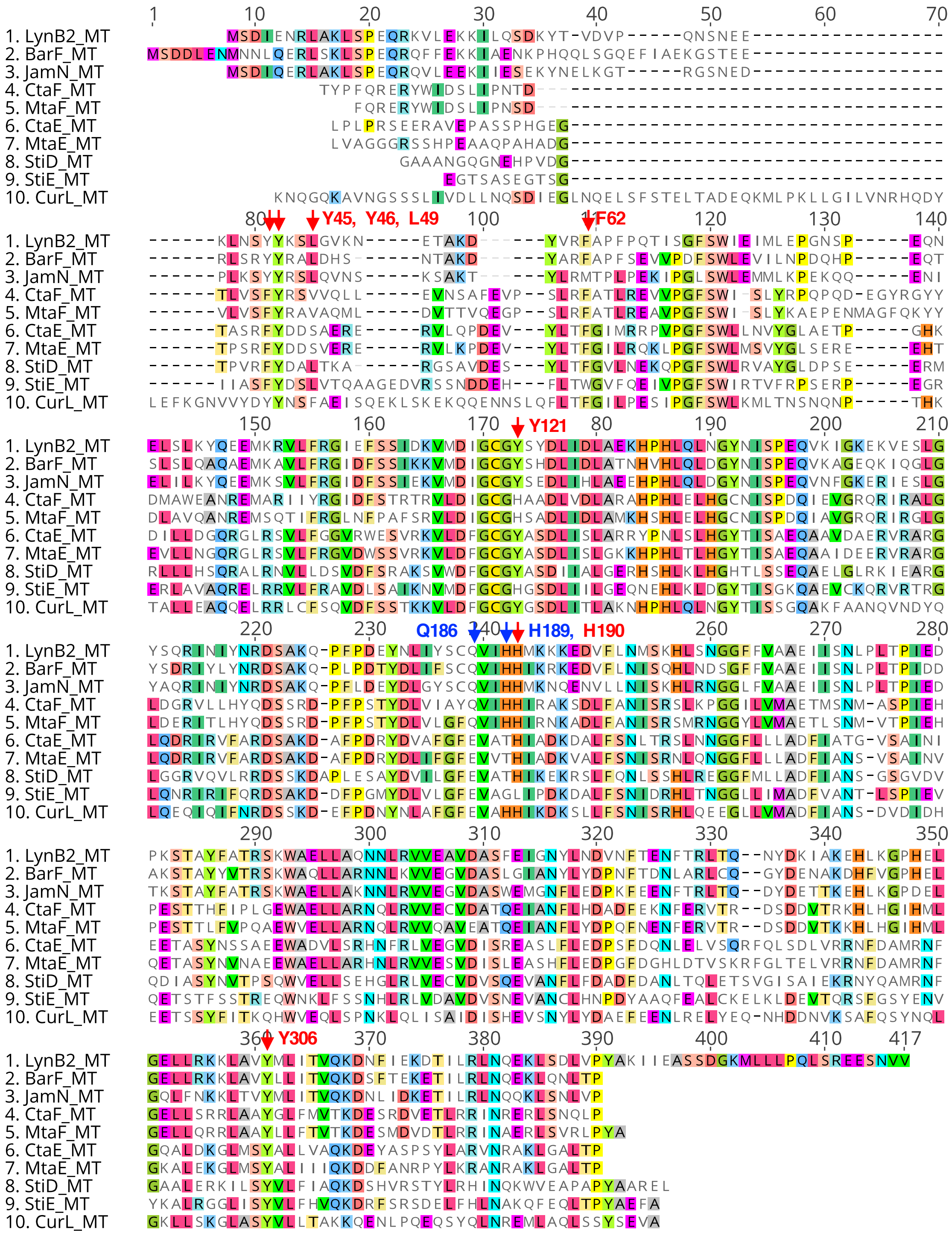

LynB2_MT (this study), BarF_MT (Q8GAQ4, LynB2_MT homolog in barbamide biosynthesis), JamN_MT (Q6E7J6, LynB2_MT homolog in jamaicamide biosynthesis), CtaF_MT (Q5MD32, PKS in cystothiazole A biosynthesis), MtaF_MT (Q9RFK6, PKS in myxothiazole A biosynthesis), CtaE_MT (Q5MD33, PKS in cystothiazole A biosynthesis), MtaE_MT (Q9RFK7, PKS in myxothiazole A biosynthesis), StiD_MT (Q8RJY3, PKS in stigmatetellin biosynthesis), StiE_MT (Q8RJY2, PKS in stigmatetellin biosynthesis), CurL_MT (Q6DNE1, PKS in curacin biosynthesis).

These were aligned via MUSCLE alignment method using the Geneious R11 software version 11.1.5 (Biomatters, Auckland, New Zealand). The amino acid residues that are supposed to involved in the binding of SAM and catalytic mechanism are labeled in red (for SAM binding) and blue (for possible catalytic residues).

Figure S10. A proposed mechanism of LynB2-OMT-catalyzed reaction.

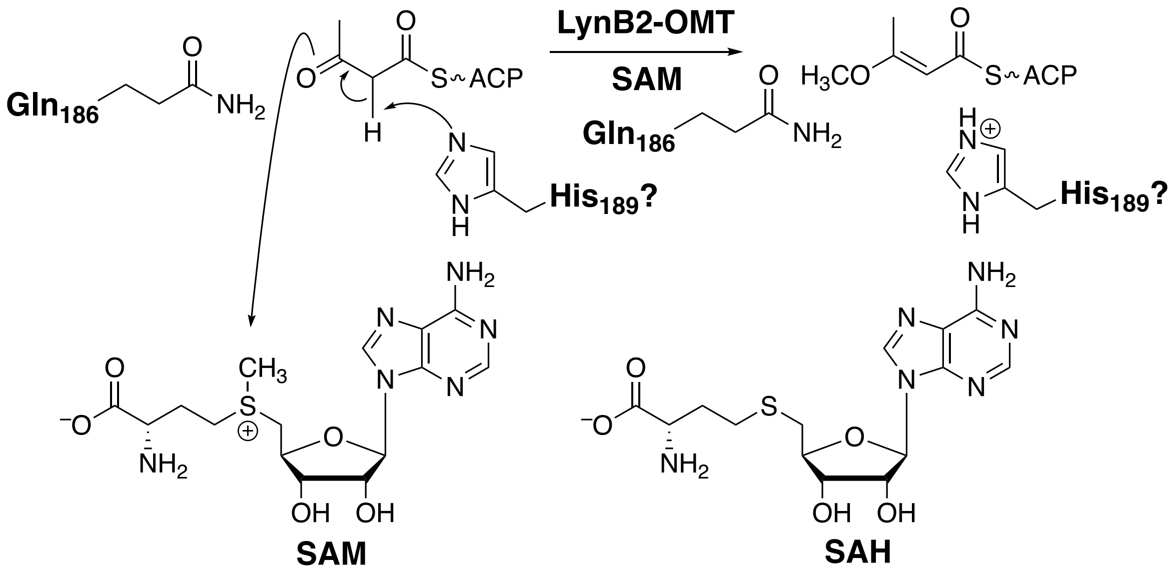

Figure S11. NMR of (4*R*)- and (4*S*)-2-methyl-2-thiazoline-4-carboxylic acids for this study.

^{1}H-NMR (400 MHz, D_2O) of (4*R*)-2-methyl-2-thiazoline-4-carboxylic acid

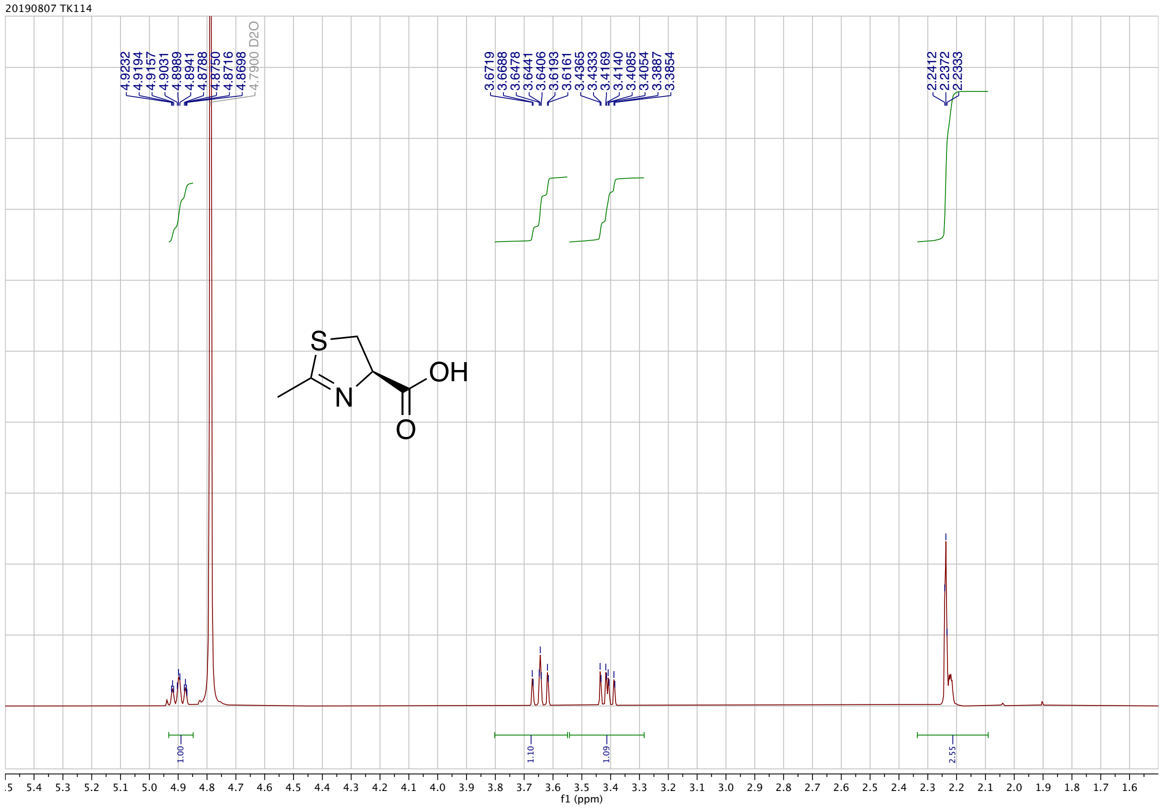

^{13}C-NMR (100 MHz, D_2O) of (4*R*)-2-methyl-2-thiazoline-4-carboxylic acid

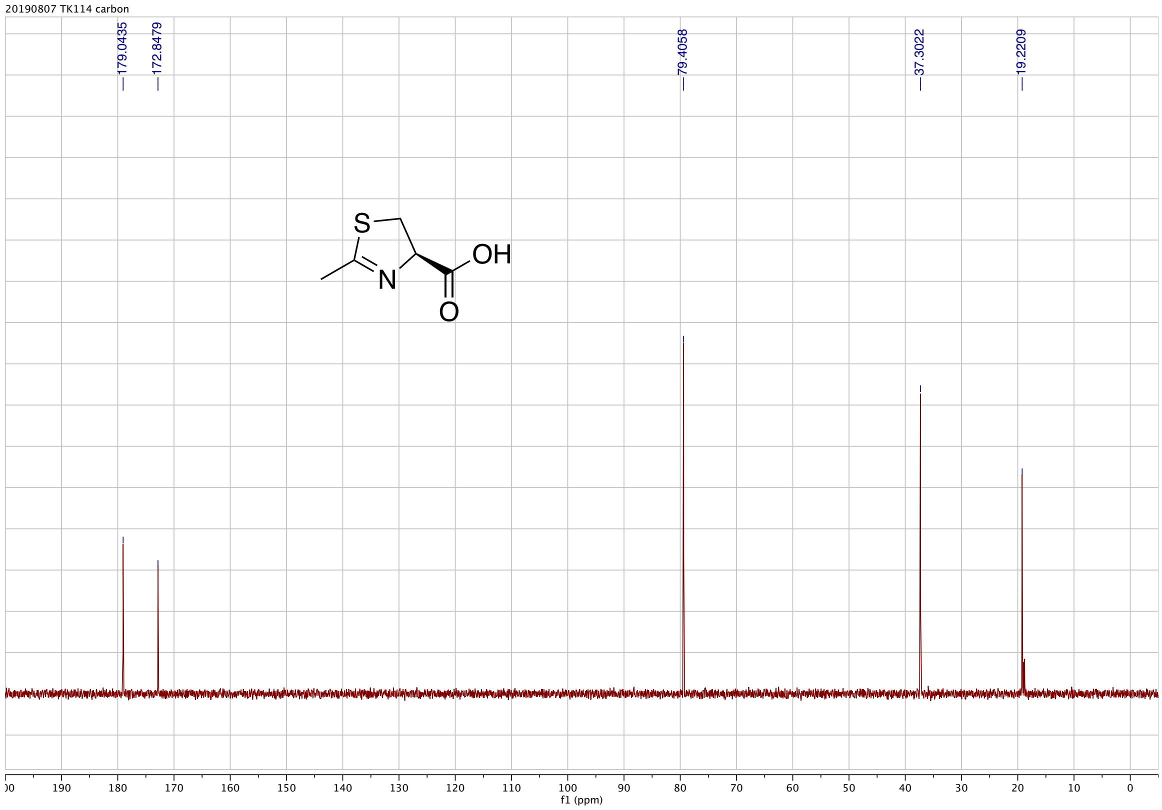

^{1}H-NMR (400 MHz, D_2O) of (4*S*)-2-methyl-2-thiazoline-4-carboxylic acid

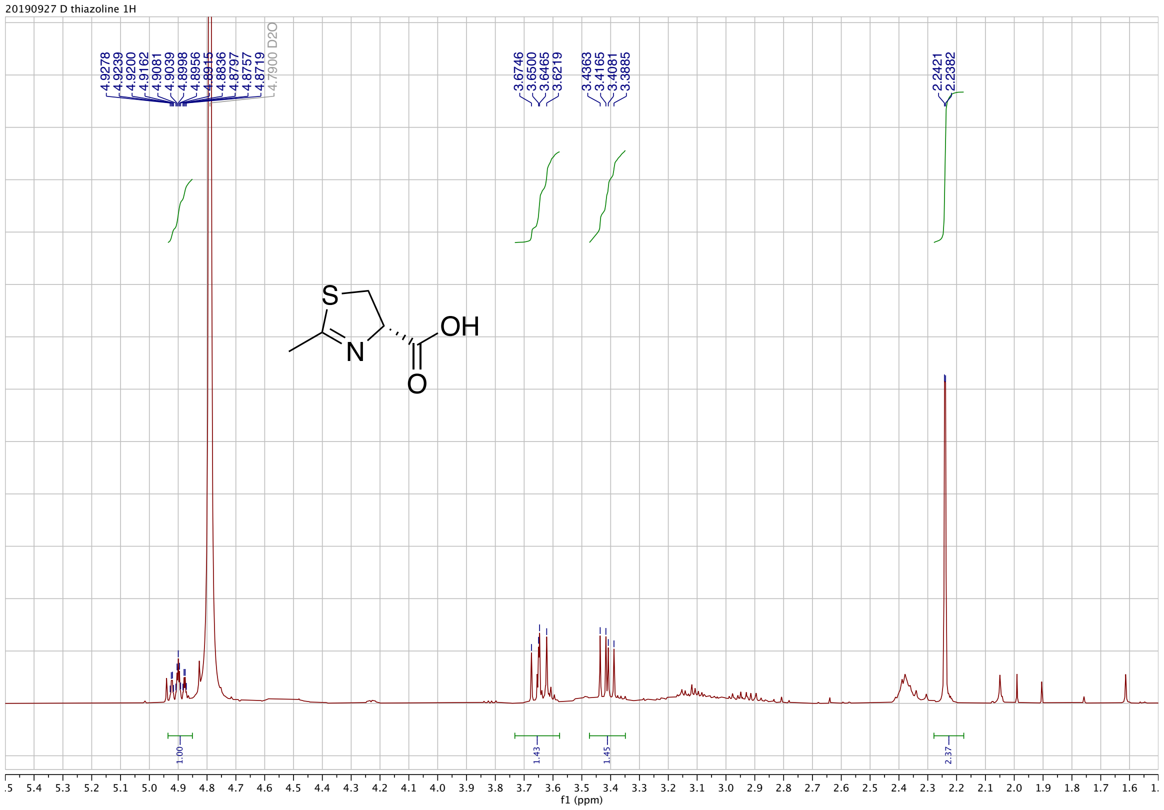

^{13}C-NMR (100 MHz, D_2O) of (4*S*)-2-methyl-2-thiazoline-4-carboxylic acid

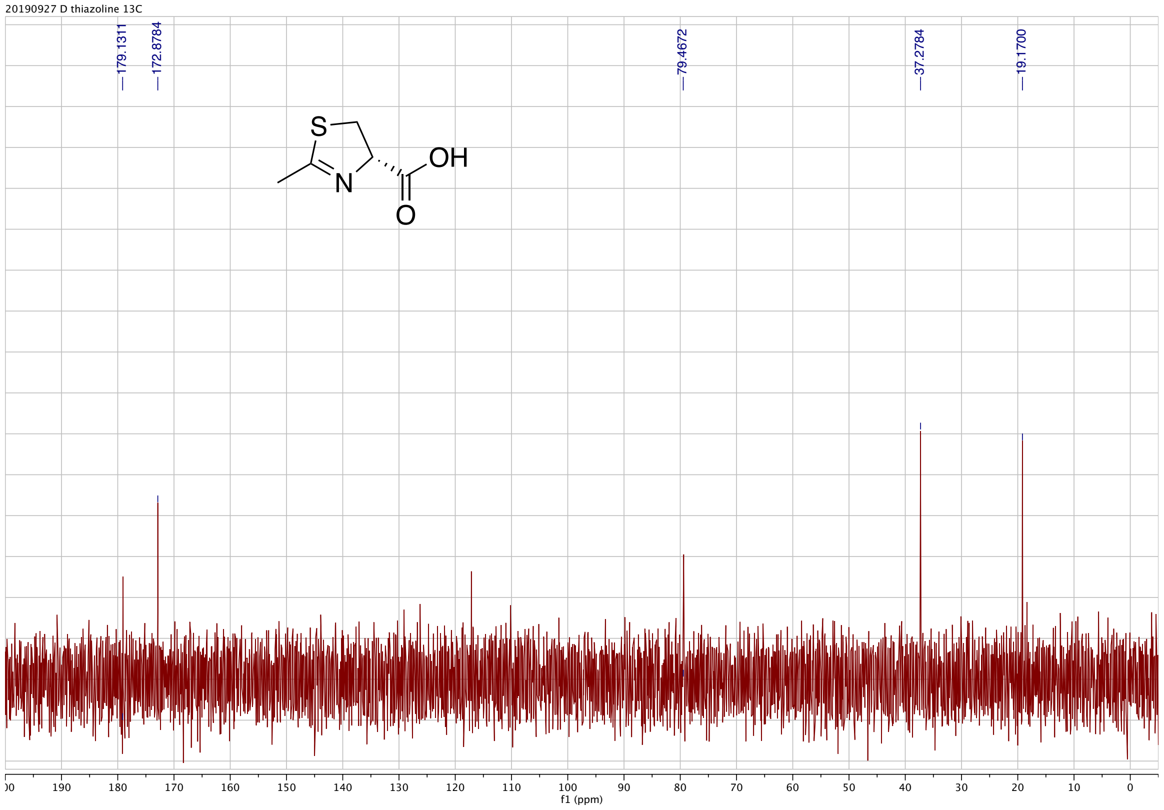

Figure S12. HPLC analysis of LynB7 reaction with (4*S*)-2-methyl-2-thiazoline-4-carboxylic acid.

(A) LynB7 reaction with 2 mM (4*S*)-2-methyl-2-thiazoline-4-carboxylic acid and 50 µM LynB7 in the presence of 1 mM Fe^{2+}, (B) mass spectrum of 2-methylthiazole produced by LynB7.

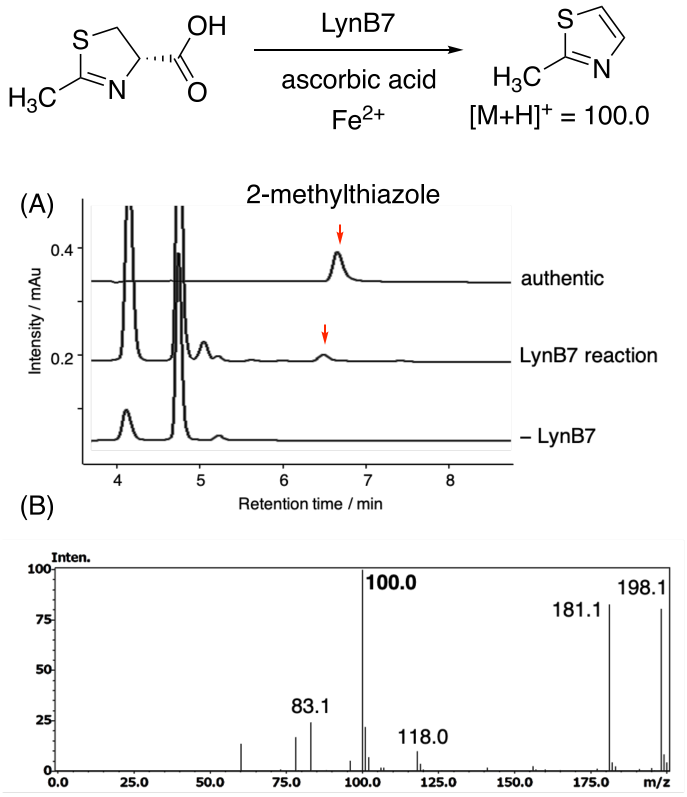

Figure S13. Kinetic analysis of LynB7 with (A) (4*R*)-2-methyl-2-thiazoline-4-carboxylic acid, (B) (4*S*)-2-methyl-2-thiazoline-4-carboxylic acid, and (C) (4*R*)-2-phenyl-2-thiazoline-4-carboxylic acid.

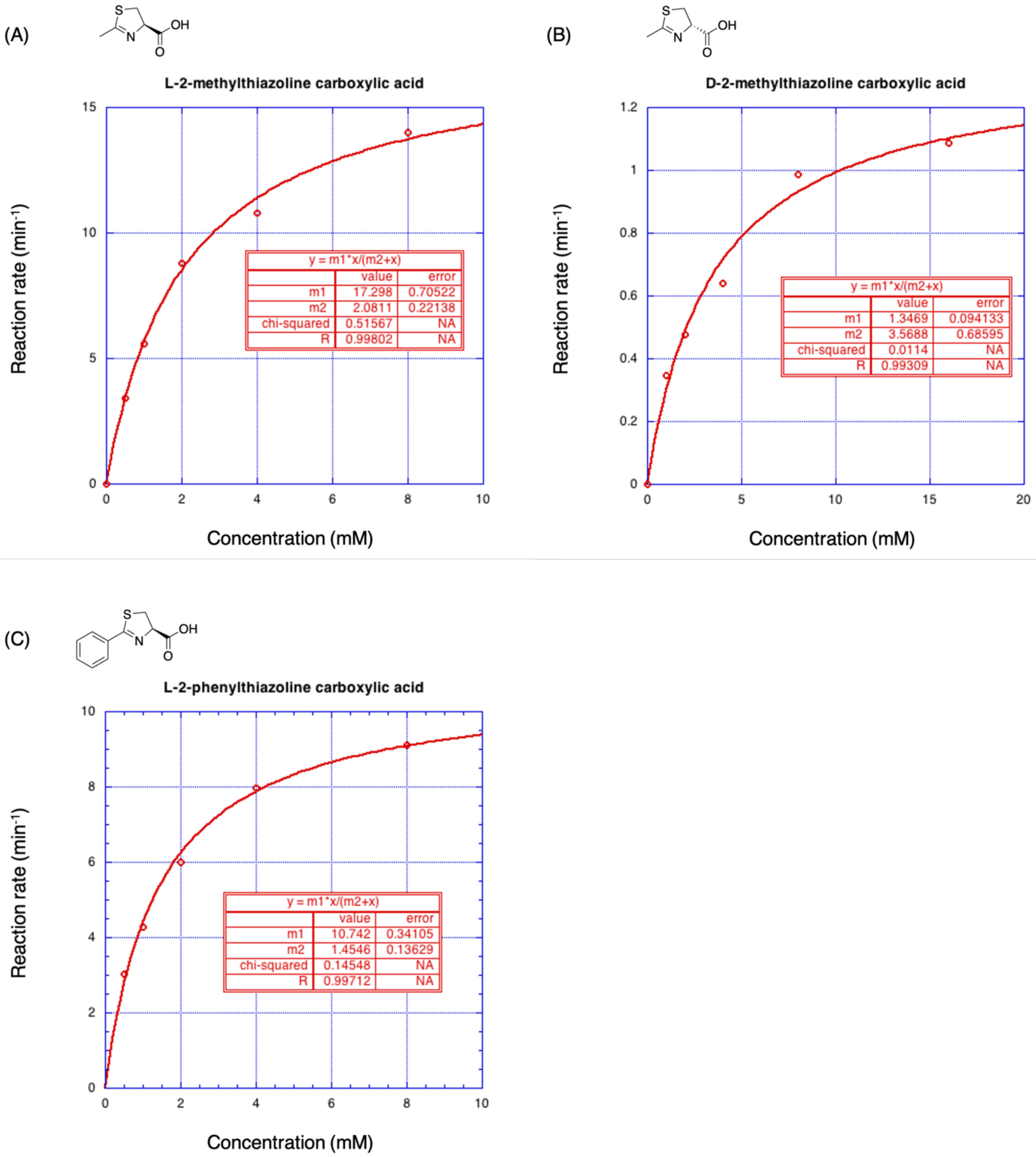

Figure S14. NMR of (4*R*)-2-phethyl-2-thiazoline-4-carboxylic acid for this study.

^{1}H-NMR (600 MHz, $CDCl_3$) of methyl (4*R*)-2-phethyl-2-thiazoline-4-carboxylate.

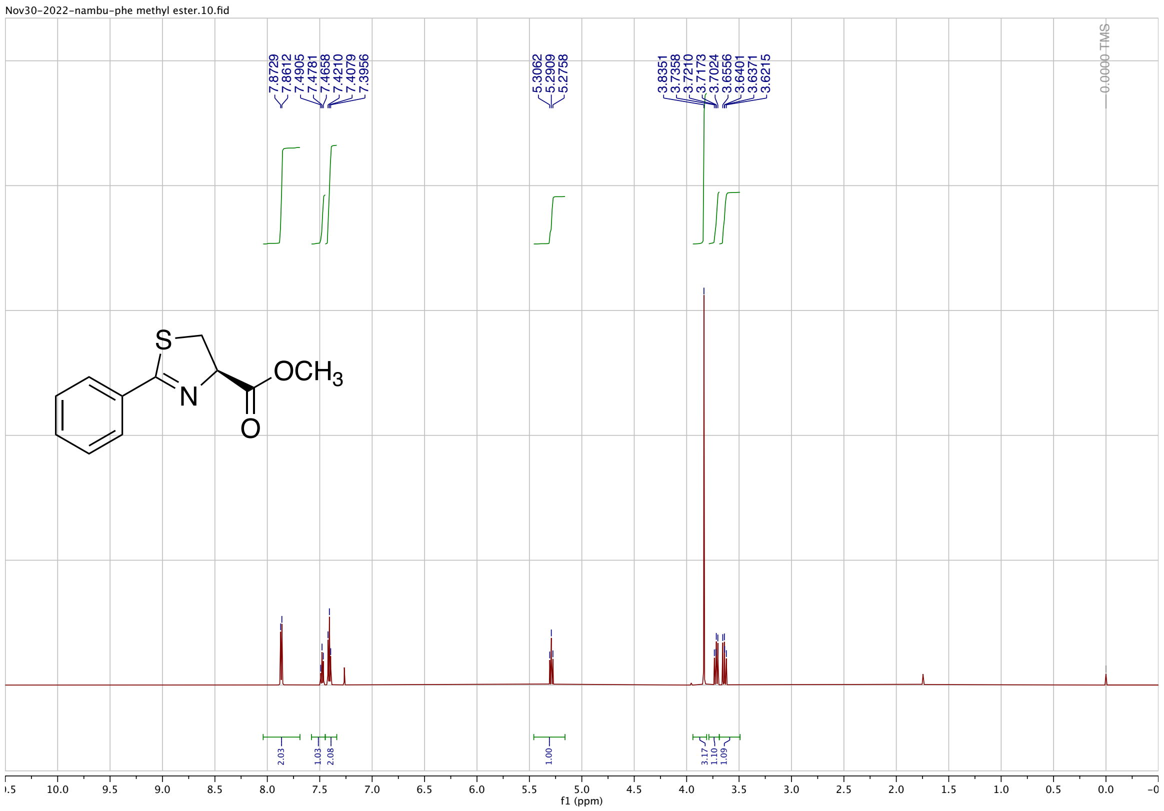

^{13}C-NMR (150 MHz, $CDCl_3$) of methyl (4*R*)-2-phethyl-2-thiazoline-4-carboxylate.

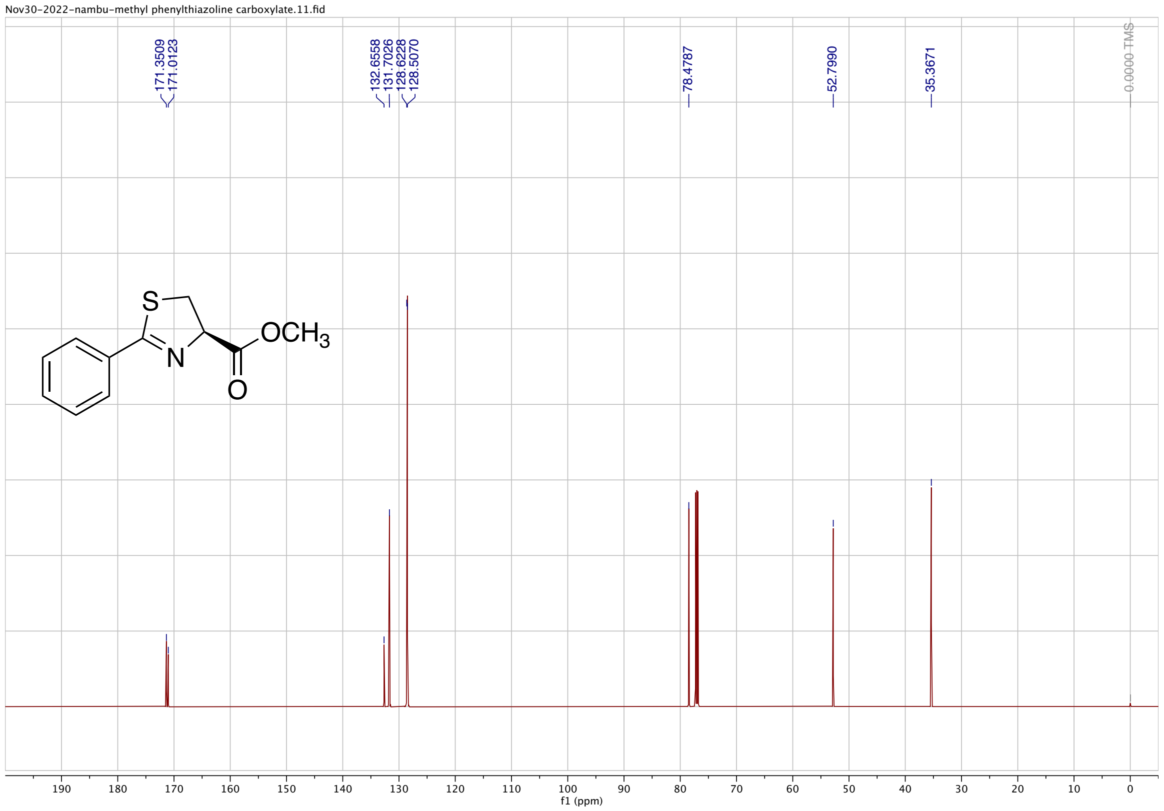

^{1}H-NMR (600 MHz, $CDCl_3$) of (4*R*)-2-phethyl-2-thiazoline-4-carboxylic acid.

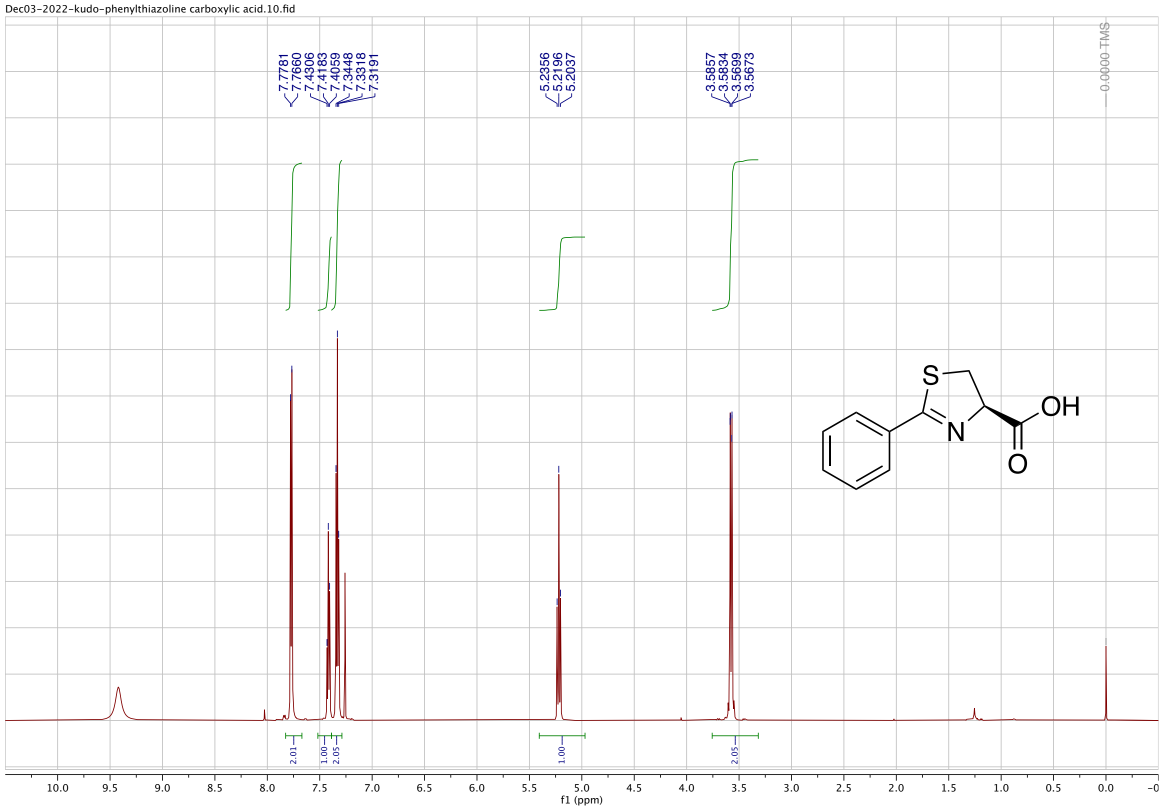

^{13}C-NMR (150 MHz, $CDCl_3$) of (4*R*)-2-phethyl-2-thiazoline-4-carboxylic acid.

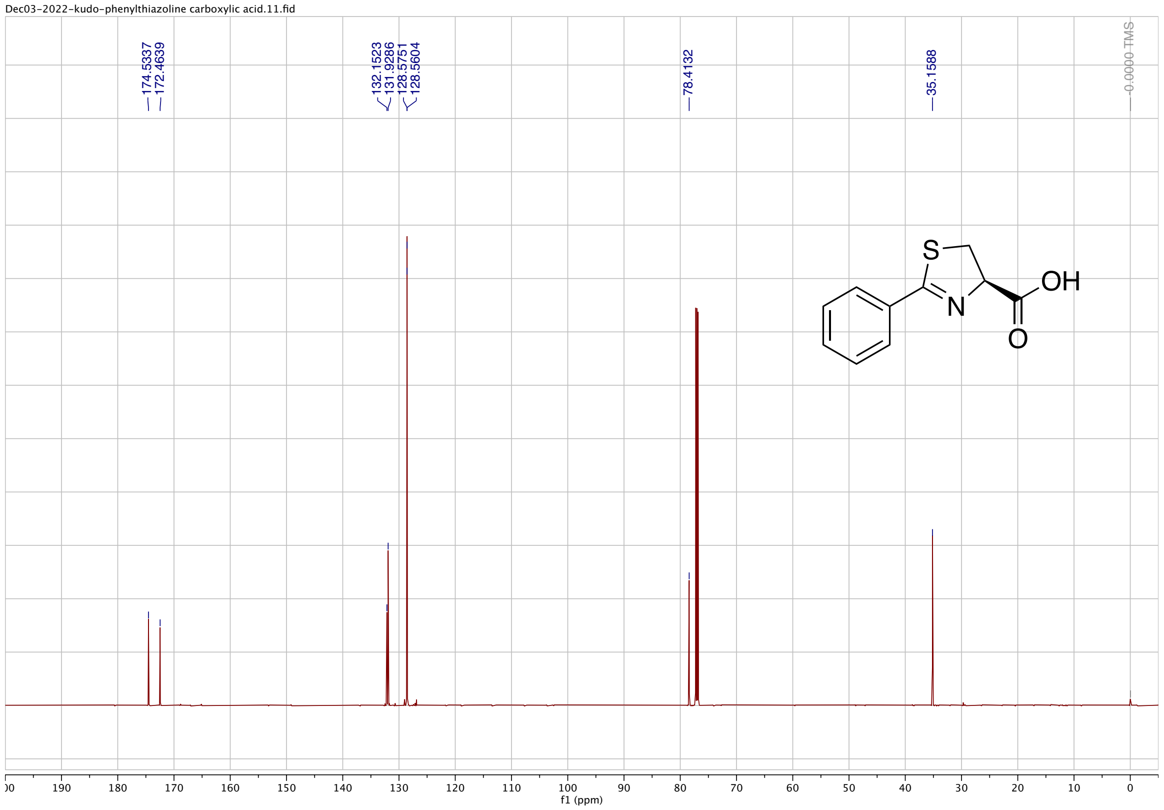

Figure S15. HPLC analysis of LynB7 reaction with (4*R*)-2-phenyl-2-thiazoline-4-carboxylic acid. (a) 2 mM (4*R*)-2-phenyl-2-thiazoline-4-carboxylic acid, 50 μM LynB7 in the presence of Fe^{2+}, (b) without LynB7, (c) 2-phenylthiazole (authentic).

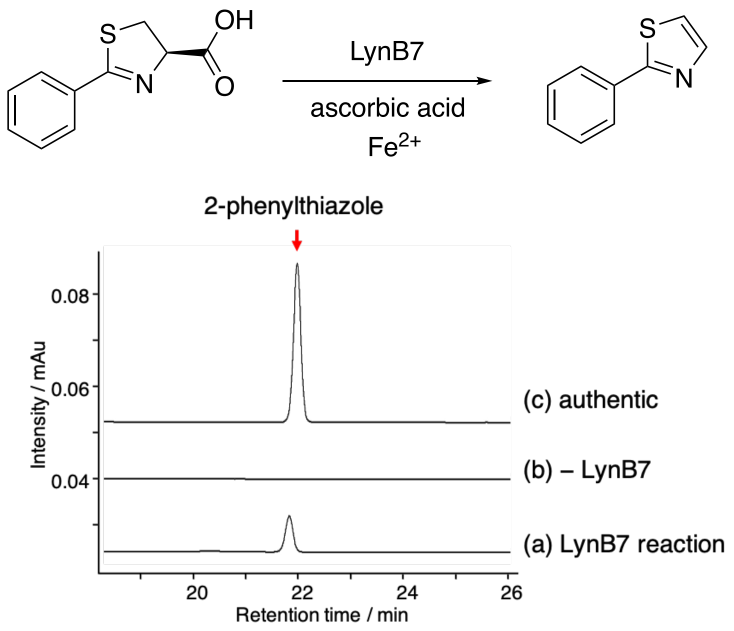

Figure S16. NMR of (4*R*)-2-phenyl-2-thiazoline-4-carboxamide for this study.

^{1}H-NMR (600 MHz, $CDCl_3$) of (4*R*)-2-phenyl-2-thiazoline-4-carboxamide.

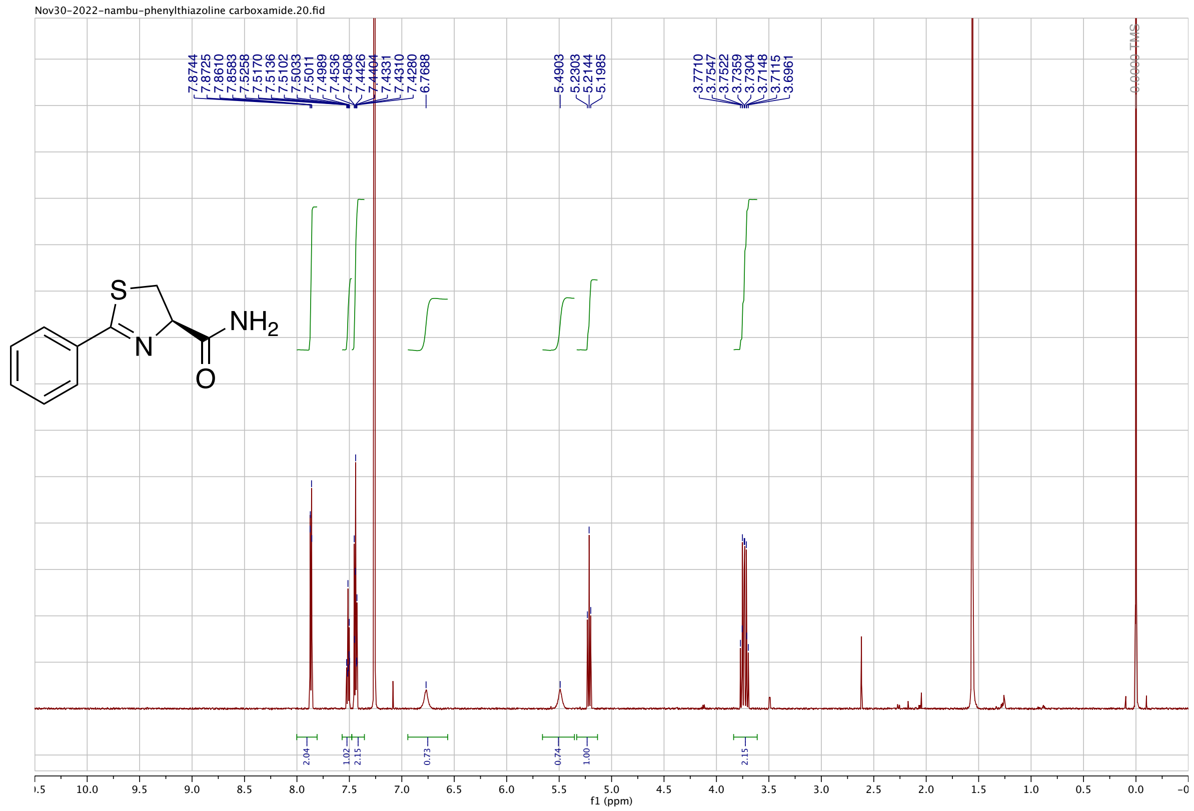

^{13}C-NMR (150 MHz, $CDCl_3$) of (4*R*)-2-phenyl-2-thiazoline-4-carboxamide.

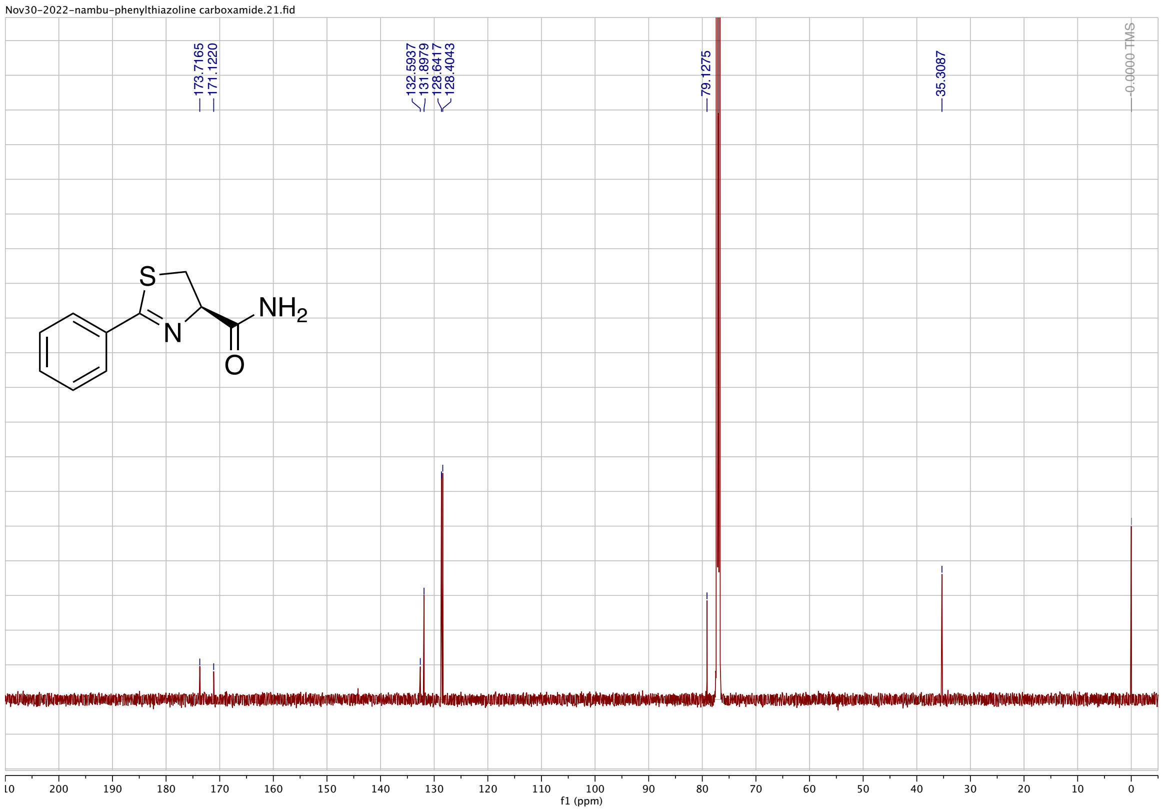

Figure S17. HPLC analysis of LynB7 reaction with (4*R*)-2-phenyl-2-thiazoline-4-carboxamide (A) and 2-phenyl-1,3-thiazole-4-carboxylic acid (B).

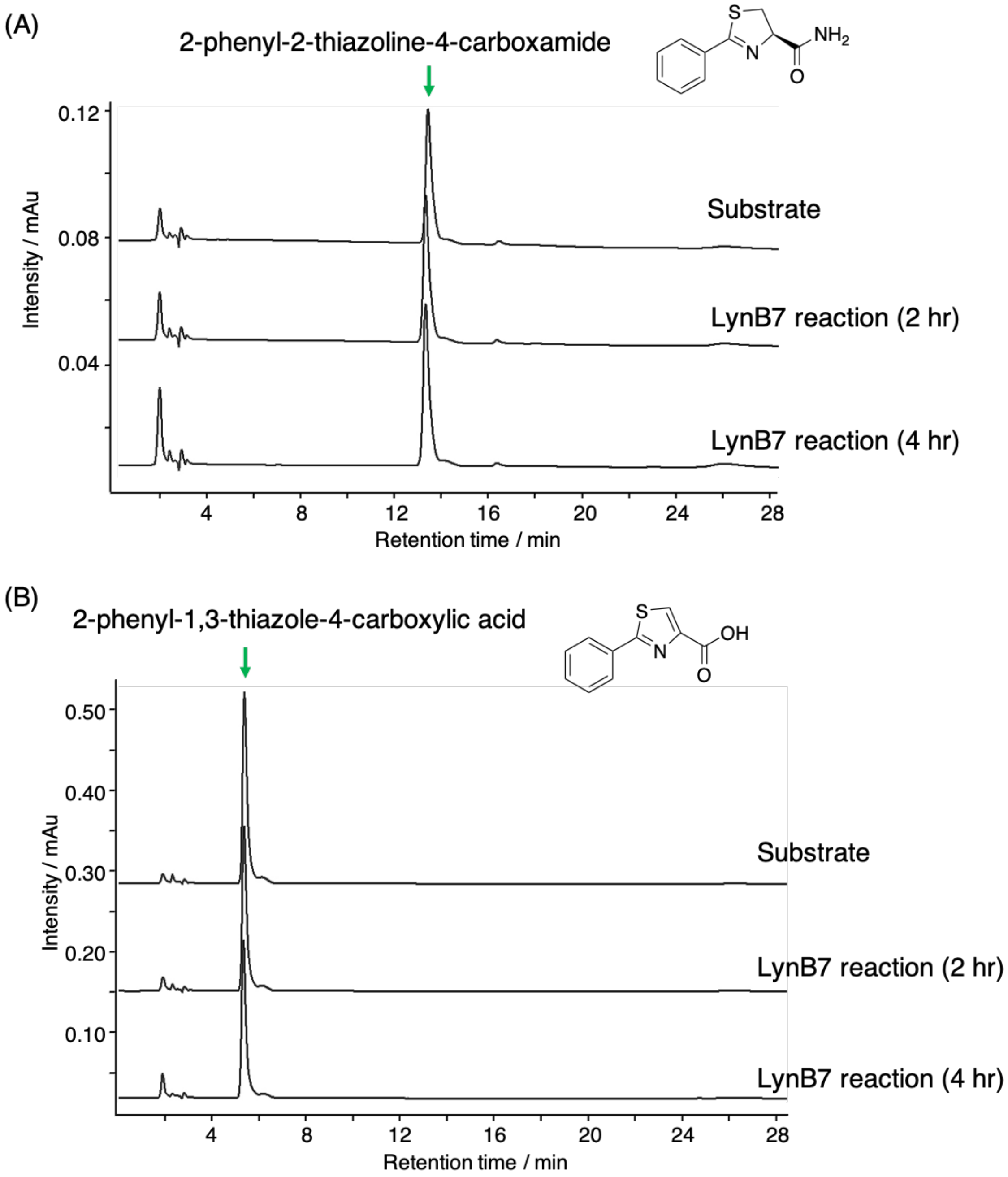

Figure S18. NMR of (*R*)-[5,5-$^2H_2$]-2-phenyl-2-thiazoline-4-carboxylic acid.

^{1}H-NMR (600 MHz, $CDCl_3$) of (*R*)-[5,5-$^2H_2$]-2-phenyl-2-thiazoline-4-carboxylic acid.

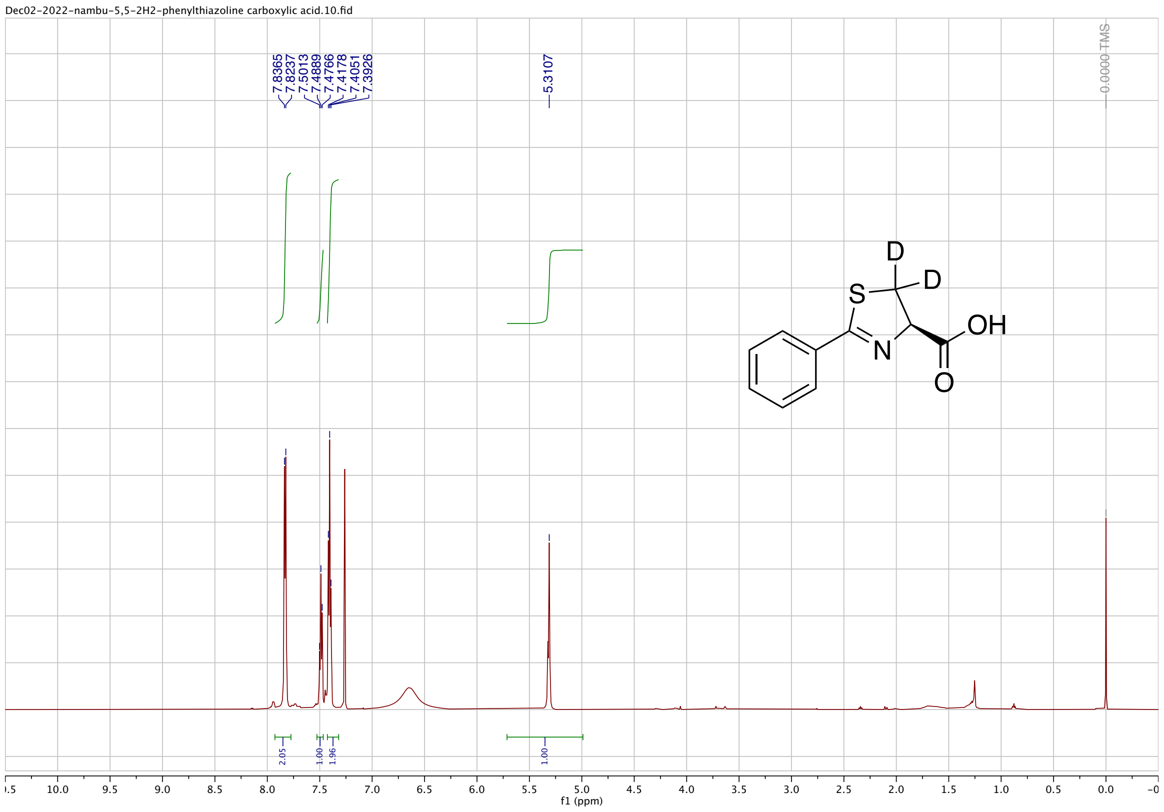

^{13}C-NMR (150 MHz, $CDCl_3$) of (*R*)-[5,5-$^2H_2$]-2-phenyl-2-thiazoline-4-carboxylic acid.

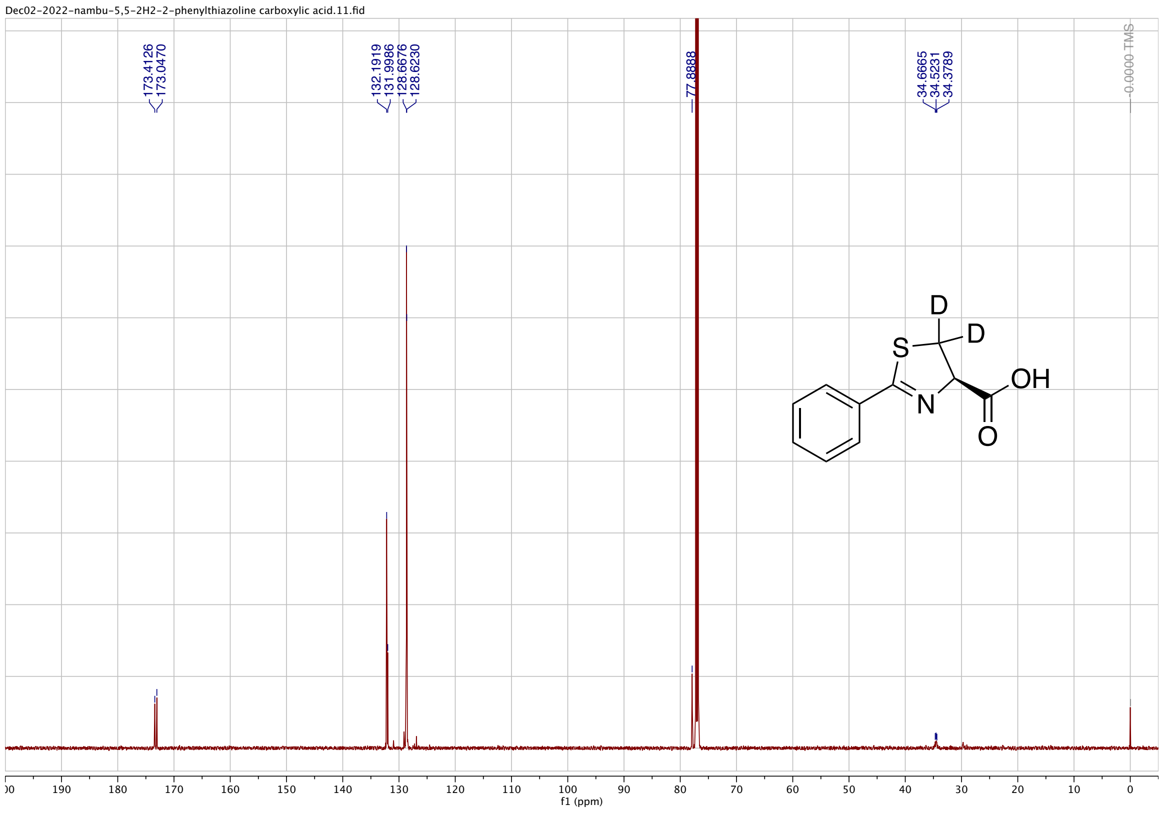

^{1}H-NMR (600 MHz, $CDCl_3$) of non-labelled and (*R*)-[5,5-$^{2}H_2$]-2-phenyl-2-thiazoline-4-carboxylic acid.

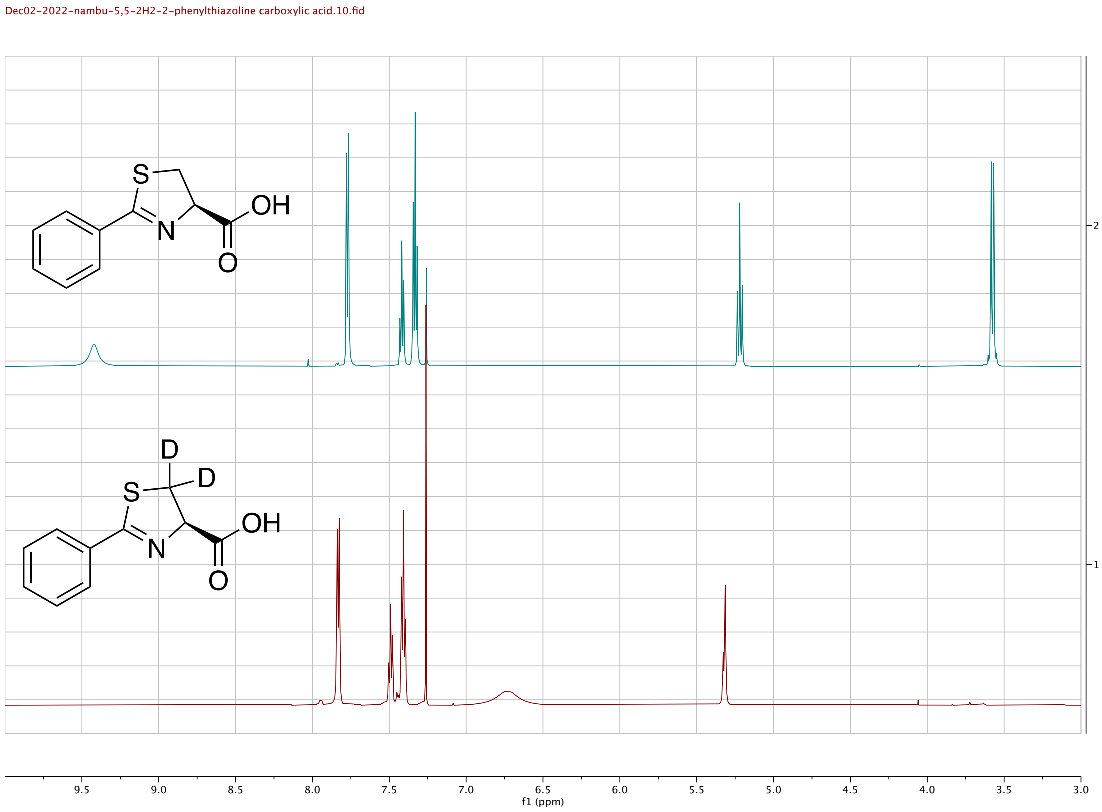

^{13}C-NMR (150 MHz, $CDCl_3$) of non-labelled and (*R*)-[5,5-$^{2}H_2$]-2-phenyl-2-thiazoline-4-carboxylic acid.

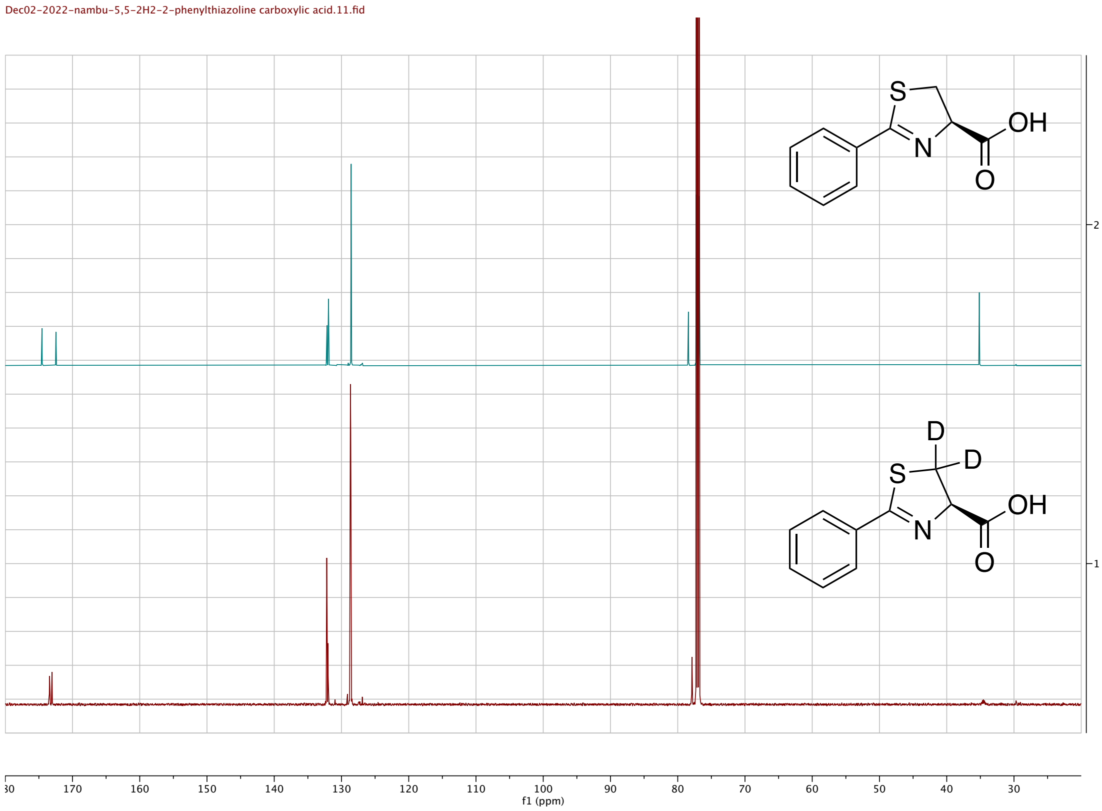

Figure S19. LynB7 reaction with (*R*)-2-phenyl-2-thiazoline-4-carboxylic acid and (*R*)-[5,5-$^2H_2$]-2-phenyl-2-thiazoline-4-carboxylic acid.

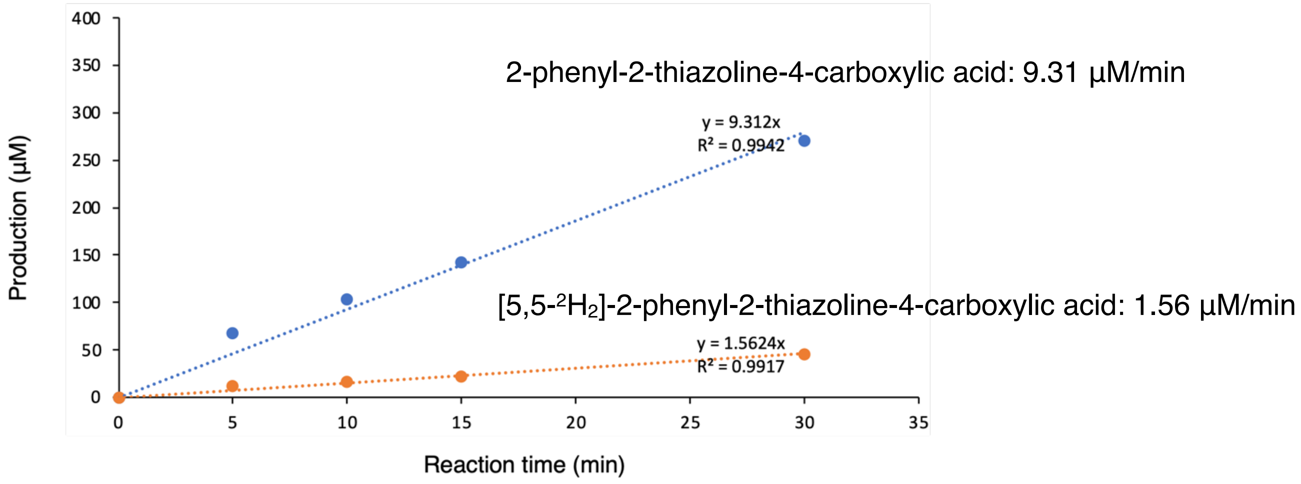

KIE was estimated to be 5.9 ± 0.2.

Figure S20. Proposed mechanisms of LynB7-catalyzed reaction.

(A) Through C5-hydroxylation of 2-thiazoline-4-carboxylic acid or (B) through β-cleavage that is initiated by the putative C5 radical.

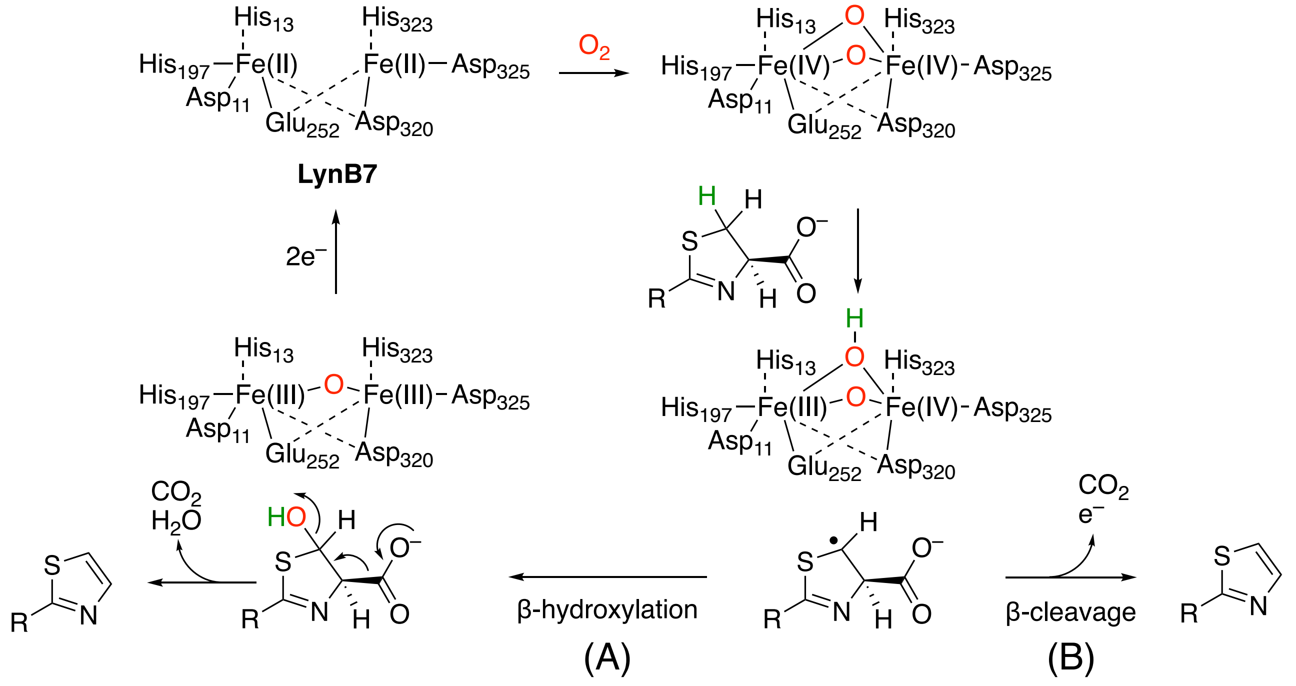

Figure S21. Model structure of LynB7. (A) Predicted model structure of LynB7 (green) is superimposed on the structure of PtmU3 (PDB entry 6omq, RMSD = 1.7 Å, cyan). (B) Putative active site of LynB7 (green) superimposed on the active site of PtmU3 (cyan). Mn atoms in the active site of PtmU3 are shown as gray spheres. The substrate of PtmU3 is shown as purple sticks. Putative residues binding to Fe atoms are shown as sticks. (C) Biosynthesis of platensimycin and platencin. PtmU3 catalyzes hydroxylation at C-5 of the CoA substrates in the biosynthetic pathways.

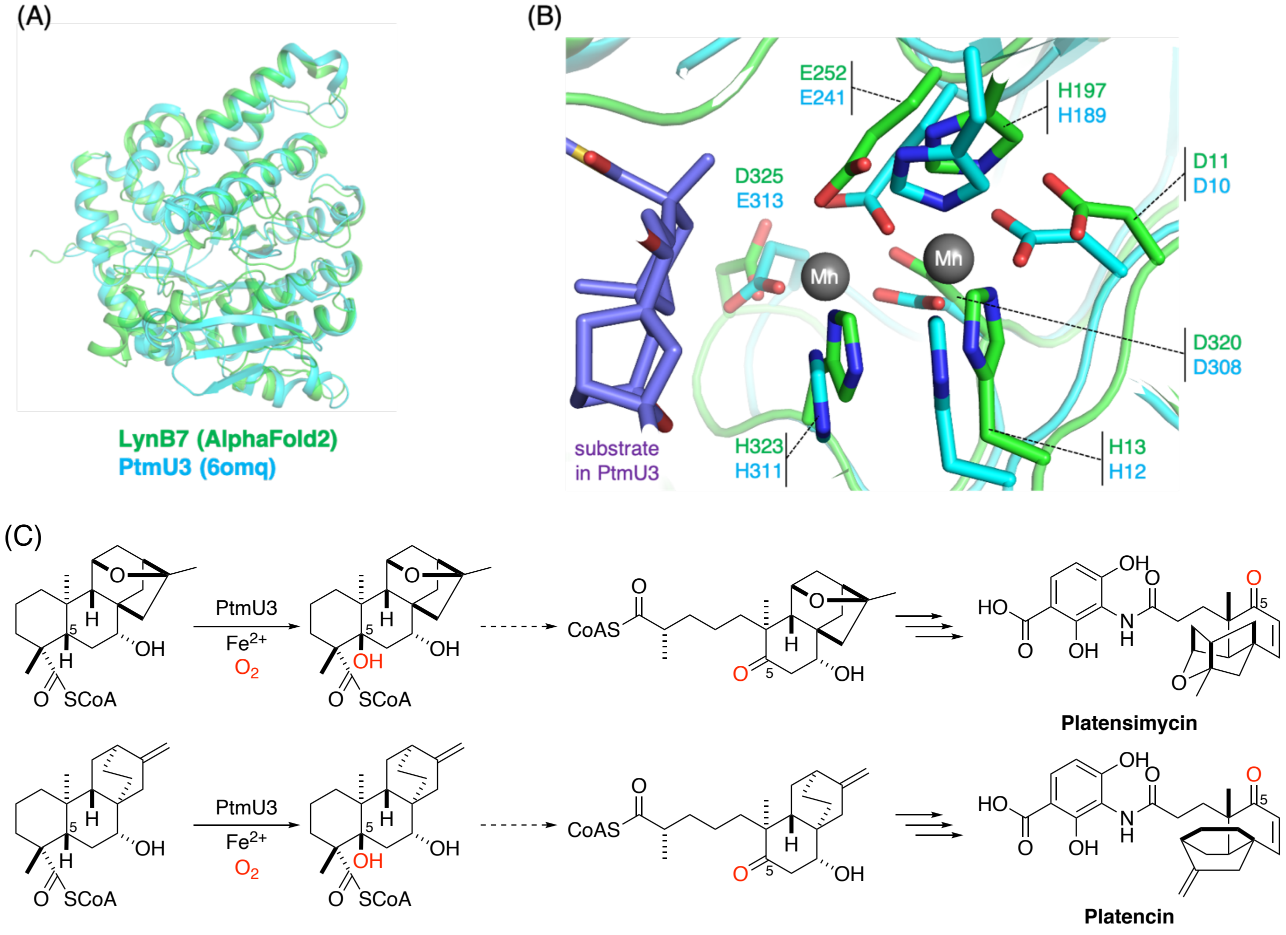

Figure S22. Sequence alignment of the LynB7 family proteins.

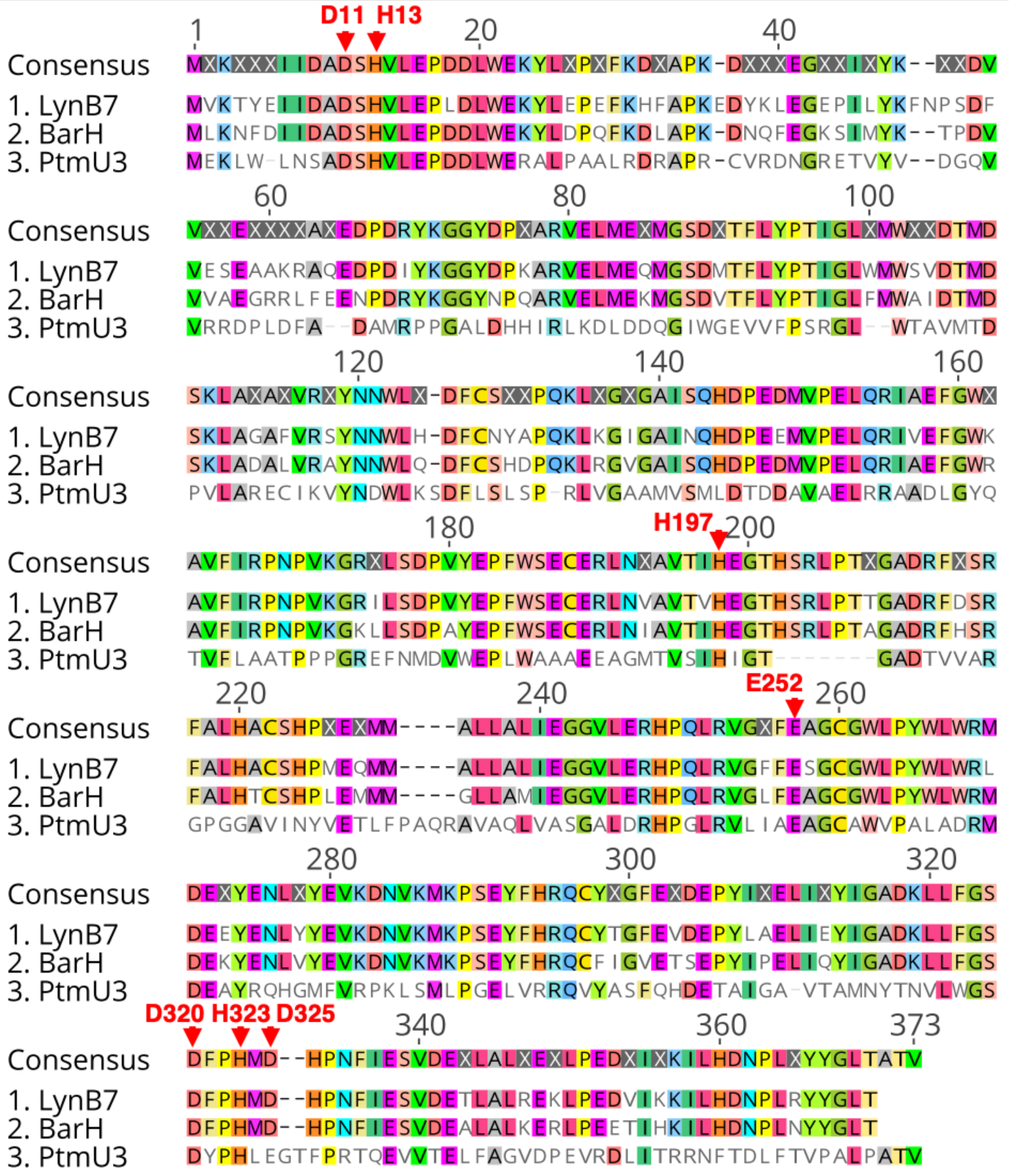

LynB7 (this study), BarH (Q8GAQ2, LynB7 homolog in barbamide biosynthesis), PtmU3 (D8L2U2, nonheme diiron monooxygenase in platensimycin biosynthesis).

These were aligned via MUSCLE alignment method using the Geneious R11 software version 11.1.5 (Biomatters, Auckland, New Zealand). The amino acid residues that are supposed to involved in the binding of bimetal cations are labeled in red.

Figure S23. Sequence alignment of (A) putative C-terminal docking domains (Cdd) and (B) N-terminal docking domains (Ndd) of LynB PKSs and NRPSs.

(A) Cdd alignment

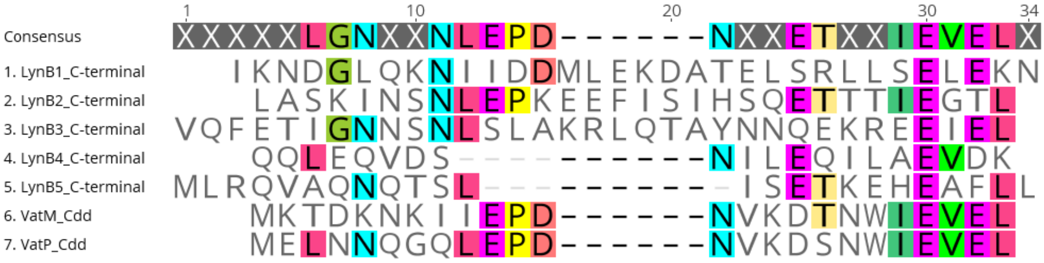

(B) Ndd alignment

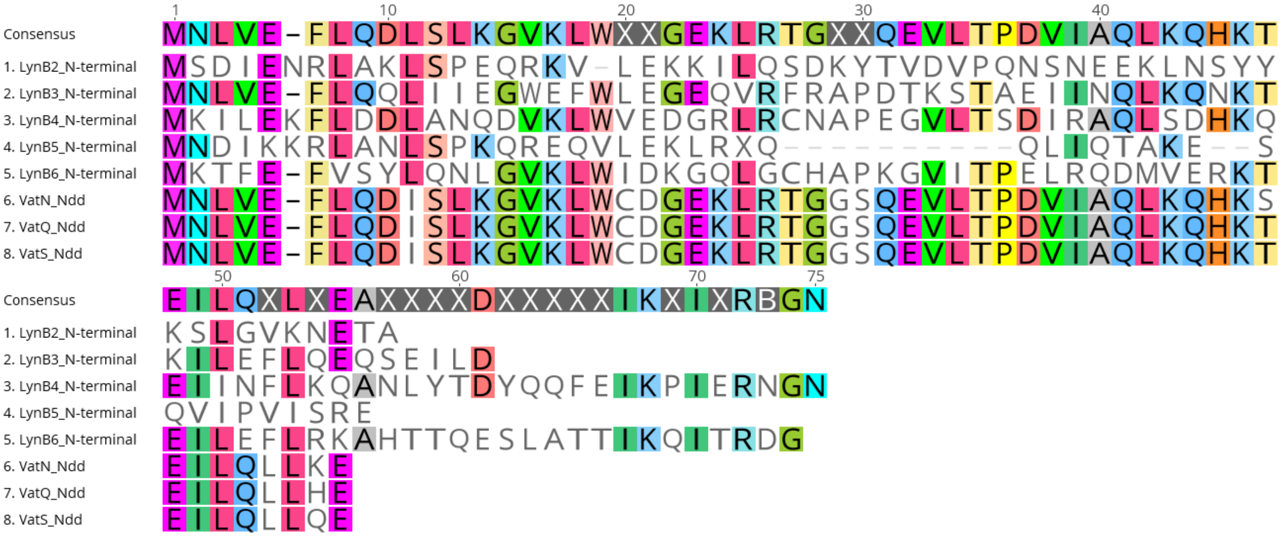

LynB1, B2, B3, B4, B5, and B6 (this study), VatM, VatP, VatN, VatQ, and VatS are PKS/NRPSs in vatiamides biosynthesis.[10]

These were aligned via MUSCLE alignment method using the Geneious R11 software version 11.1.5 (Biomatters, Auckland, New Zealand). The amino acid residues that are supposed to involved in the binding of bimetal cations are labeled in red.

References

(1) Pringsheim, E. G. (2016) *Pure Cultures of Algae*, Cambridge University Press.

(2) Quadri, L. E., Weinreb, P. H., Lei, M., Nakano, M. M., Zuber, P., and Walsh, C. T. (1998) Characterization of Sfp, a Bacillus subtilis phosphopantetheinyl transferase for peptidyl carrier protein domains in peptide synthetases, *Biochemistry 37*, 1585-1595.

(3) Chisuga, T., Nagai, A., Miyanaga, A., Goto, E., Kishikawa, K., Kudo, F., and Eguchi, T. (2022) Structural Insight into the Reaction Mechanism of Ketosynthase-Like Decarboxylase in a Loading Module of Modular Polyketide Synthases, *ACS Chem. Biol. 17*, 198-206.

(4) Gilbert, I. H., Ginty, M., Oneill, J. A., Simpson, T. J., Staunton, J., and Willis, C. L. (1995) Synthesis of Beta-Keto and Alpha,Beta-Unsaturated N-Acetylcysteamine Thioesters, *Bioorg. Med.Chem. Lett. 5*, 1587-1590.

(5) Jones, A. C., Monroe, E. A., Podell, S., Hess, W. R., Klages, S., Esquenazi, E., Niessen, S., Hoover, H., Rothmann, M., Lasken, R. S., Yates, J. R., 3rd, Reinhardt, R., Kube, M., Burkart, M. D., Allen, E. E., Dorrestein, P. C., Gerwick, W. H., and Gerwick, L. (2011) Genomic insights into the physiology and ecology of the marine filamentous cyanobacterium Lyngbya majuscula, *Proc. Natl. Acad. Sci. U.S.A. 108*, 8815-8820.

(6) Chang, Z., Sitachitta, N., Rossi, J. V., Roberts, M. A., Flatt, P. M., Jia, J., Sherman, D. H., and Gerwick, W. H. (2004) Biosynthetic pathway and gene cluster analysis of curacin A, an antitubulin natural product from the tropical marine cyanobacterium Lyngbya majuscula, *J. Nat. Prod. 67*, 1356-1367.

(7) Edwards, D. J., Marquez, B. L., Nogle, L. M., McPhail, K., Goeger, D. E., Roberts, M. A., and Gerwick, W. H. (2004) Structure and biosynthesis of the jamaicamides, new mixed polyketide-peptide neurotoxins from the marine cyanobacterium Lyngbya majuscula, *Chem. Biol. 11*, 817-833.

(8) Chang, Z., Flatt, P., Gerwick, W. H., Nguyen, V. A., Willis, C. L., and Sherman, D. H. (2002) The barbamide biosynthetic gene cluster: a novel marine cyanobacterial system of mixed polyketide synthase (PKS)-non-ribosomal peptide synthetase (NRPS) origin involving an unusual trichloroleucyl starter unit, *Gene 296*, 235-247.

(9) Huntley, S., Hamann, N., Wegener-Feldbrugge, S., Treuner-Lange, A., Kube, M., Reinhardt, R., Klages, S., Muller, R., Ronning, C. M., Nierman, W. C., and Sogaard-Andersen, L. (2011) Comparative genomic analysis of fruiting body formation in Myxococcales, *Mol. Biol. Evol. 28*, 1083-1097.

(10) Moss, N. A., Seiler, G., Leao, T. F., Castro-Falcon, G., Gerwick, L., Hughes, C. C., and Gerwick, W. H. (2019) Nature's Combinatorial Biosynthesis Produces Vatiamides A-F, *Angew. Chem. Int. Ed. 58*, 9027-9031.

(11) Leao, T., Castelao, G., Korobeynikov, A., Monroe, E. A., Podell, S., Glukhov, E., Allen, E. E., Gerwick, W. H., and Gerwick, L. (2017) Comparative genomics uncovers the prolific and distinctive metabolic potential of the cyanobacterial genus Moorea, *Proc. Natl. Acad. Sci. U.S.A. 114*, 3198-3203.

(12) Ronnebaum, T. A., McFarlane, J. S., Prisinzano, T. E., Booker, S. J., and Lamb, A. L. (2019) Stuffed Methyltransferase Catalyzes the Penultimate Step of Pyochelin Biosynthesis, *Biochemistry 58*, 665-678.

(13) Mori, S., Pang, A. H., Lundy, T. A., Garzan, A., Tsodikov, O. V., and Garneau-Tsodikova, S. (2018) Structural basis for backbone N-methylation by an interrupted adenylation domain, *Nat. Chem. Biol. 14*, 428-430.
